# Atomic-Shell Engineering of GeS_x_-Au Nanorods Synergistically Suppresses Phonon Scattering and Activates N_2_ for Near-Infrared-Driven Ammonia Synthesis

**DOI:** 10.34133/research.1169

**Published:** 2026-02-25

**Authors:** Jia-Xing Liu, Jin-Guan Liang, Xu-Hang Zhong, Bingzhe Wang, Shang-Fu Yuan, Zhiling Yu, Yu Han, Tao Wu, Dan Li

**Affiliations:** ^1^ College of Chemistry and Materials Science and Guangdong Provincial Key Laboratory of Supramolecular Coordination Chemistry, Jinan University, Guangzhou 510632, China.; ^2^ Center for Electron Microscopy, School of Emergent Soft Matter, and State Key Laboratory of Pulp and Paper Engineering, South China University of Technology, Guangzhou 511442, China.

## Abstract

The efficient utilization of near-infrared (NIR) light remains a paramount challenge for solar-driven photocatalytic nitrogen fixation, primarily due to rapid energy dissipation of hot carriers via phonon scattering. Here, we construct a well-defined core–shell heterostructure through the in situ anchoring of an ultrathin, sulfur-vacancy-rich GeS_x_ shell onto gold nanorods (AuNRs)—an atomic-scale configuration that fundamentally suppresses phonon-mediated energy loss. This suppression, directly evidenced by transient absorption spectroscopy through reduced phonon frequency and scattering rate, prolongs the lifetime of plasmon-derived hot electrons. Combined with the vacancy-enabled activation of the robust N≡N bond at exposed Ge sites, this catalyst achieves an exceptional NH_3_ production rate of 1,225.6 μmol g^−1^ h^−1^ under NIR irradiation and a remarkable apparent quantum efficiency of 1.23% at 880 nm—a performance that rivals many ultraviolet/visible-driven systems. Through in situ spectroscopy, transient absorption studies, and theoretical analysis, we elucidate the atomic-level origin of synergistic catalysis: the GeS_x_ shell not only suppresses phonon scattering by reducing phonon frequency and velocity, but also creates electron-deficient Ge sites that function as preferential centers for N_2_ adsorption and activation, thereby bridging hot-carrier dynamics with molecular reduction kinetics. This work provides a definitive strategy for overcoming fundamental energy loss limitations in plasmonic photocatalysis by concurrently engineering the energy dissipation pathway and the atomic catalytic environment.

## Introduction

**Fig. 1.**
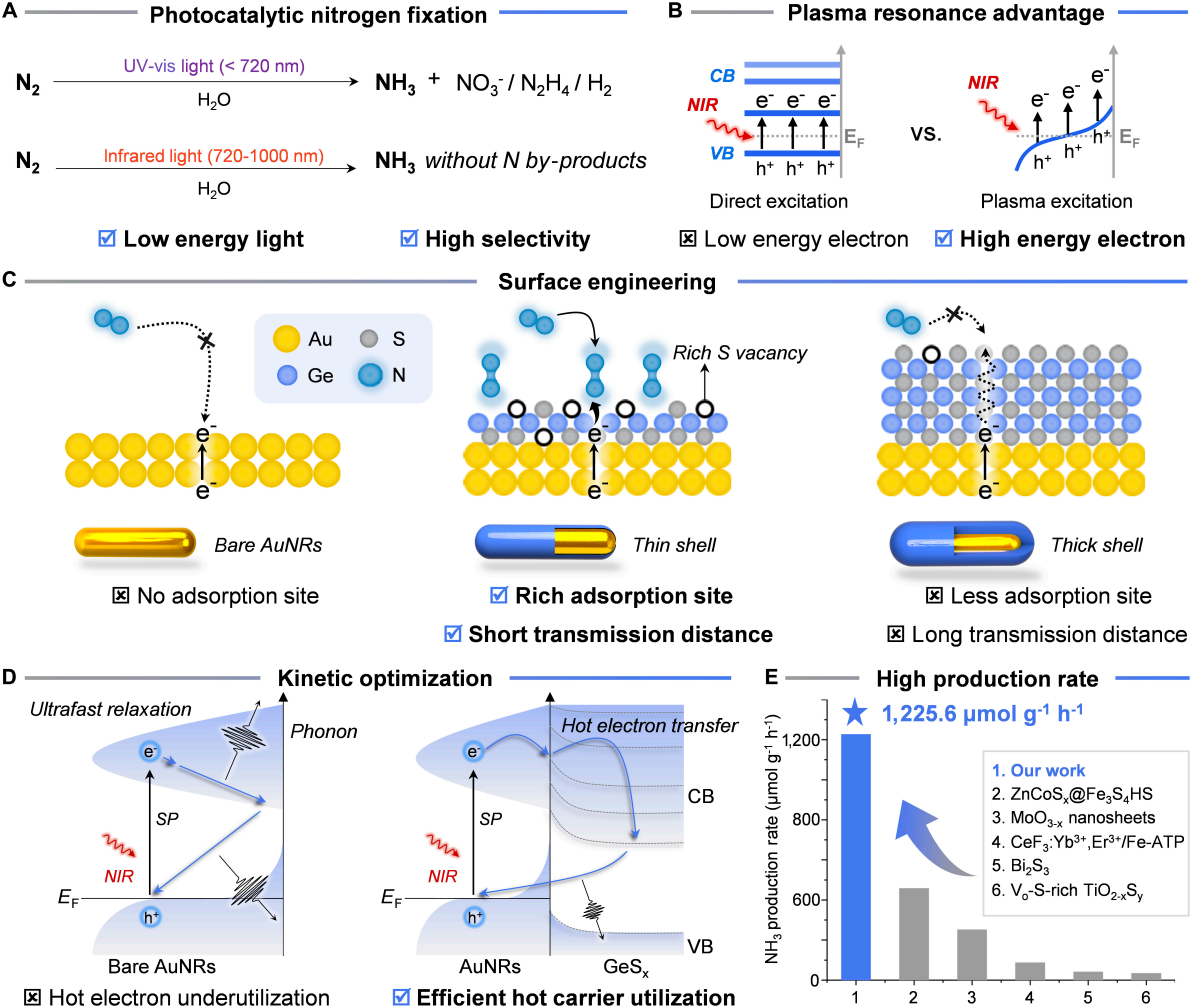
Advantages of thin-shell GeS_x_-Au in nitrogen fixation driven by NIR light. (A) Advantages of NIR photocatalytic nitrogen fixation. (B) Superiority of plasma excitation in hot electron generation over direct narrow bandgap semiconductor excitation. (C) Thin shell layer advantage of gold nanorods–semiconductors and surface/interface engineering. (D) Phonon interactions and hot electron kinetics: a comparison between bare AuNRs and AuNRs coated by a thin semiconductor layer. (E) Comparison of ammonia yield in nitrogen fixation driven by NIR light.

Ammonia (NH_3_), regarded as “fixed nitrogen”, is a pivotal precursor for synthetic nitrogen-based fertilizers and bulk chemicals, also gaining interest as a promising carbon-free energy carrier [1,2]. Industrially, NH_3_ is predominantly manufactured through the energy-intensive Haber–Bosch process, which accounts for over 1% of global energy production and results in substantial CO_2_ emissions [3,4]. Photocatalytic nitrogen fixation technology presents a revolutionary paradigm for green synthesis by enabling the direct solar-driven conversion of N_2_ and H_2_O into NH_3_. However, the practical implementation of this technology faces several fundamental challenges. A major obstacle is the prohibitively high dissociation energy barrier (946 kJ/mol) of the N≡N triple bond, which imposes stringent requirements on catalytic activation capacity. Moreover, conventional photocatalysts—such as semiconductor heterojunctions, single-atom catalysts, and noble metal clusters—depend mainly on high-energy ultraviolet/visible (UV/vis) excitation, thereby utilizing only ∼50% of the solar spectrum. Consequently, near-infrared (NIR) photons, which constitute nearly half of solar energy, remain severely underexploited [4–7]. Although NIR-driven photocatalytic systems demonstrate enhanced reaction selectivity by suppressing competing reactions such as hydrogen evolution and the formation of by-products (e.g., nitrate and hydrazine), their current efficiency in N_2_ activation remains limited due to the low energy of NIR photons. As a result, NIR-based systems typically exhibit NH_3_ production rates that are 1 to 2 orders of magnitude lower than those achieved under UV/vis illumination (Fig. 1A) [8].

Au nanorods (AuNRs), as one-dimensional anisotropic plasmonic nanomaterials, have gained prominence as exceptional NIR photosensitizers by virtue of their size/aspect ratio-dependent optical properties. Through aspect ratio modulation, AuNRs’ localized surface plasmon resonance (LSPR) absorption can be precisely tuned from visible (520 nm) to NIR II regions (1,300 nm) [9–12]. The LSPR phenomenon originates from collective coherent oscillations of surface free electrons upon photon excitation, leading to intense electromagnetic field enhancement at structural termini and the generation of high-density hot carriers (hot electrons and holes) under resonance conditions. Particularly in the NIR spectrum, AuNRs exhibit superior longitudinal LSPR modes with absorption cross-sections 50 to 100 times their geometric volume, substantially surpassing conventional semiconductor materials (Fig. 1B). However, the utility of these hot carriers is severely limited by their ultrafast relaxation (on the order of 100 fs to 1 ps) through electron–phonon scattering, which efficiently converts their excess energy into lattice vibrations (heat) on a timescale far shorter than that of typical catalytic turnover before they can be harnessed for chemistry [9,11].

The conventional heterojunction strategy of coupling AuNRs with semiconductors represents a widely adopted approach for leveraging hot-electron transfer in plasmonic systems [13–15]. For instance, Wu et al. [16] demonstrated enhanced photocatalytic hydrogen production by constructing dumbbell-shaped heterostructures with TiO_2_ and AuNRs. Wan et al. [17] achieved improved CO_2_ reduction efficiency through the simultaneous integration of n-type CdS and p-type PbS heterojunctions onto AuNRs. While coupling semiconductors to AuNRs can extract electrons, conventional synthetic methods often produce thick, polycrystalline shells. This introduces new problems: long charge migration paths, increased defect-mediated recombination, and the shielding of active sites, which collectively diminish the promised gains from plasmonic excitation. In conventional core@shell nanostructures, a thick semiconductor shell completely encapsulates the plasmonic metal core. This configuration introduces severe limitations: the extended migration path for hot electrons to reach the shell surface, along with the inability to efficiently extract hot holes from the metal core, results in substantial recombination and energy dissipation of hot carriers. Moreover, inadequate interfacial contact—both within AuNRs and at Au-semiconductor junctions—promotes charge recombination at structural defects [18,19]. The suboptimal density and insufficient exposure of surface active sites further restrict effective substrate activation at the reactive interface, a limitation well-documented in previous studies (Fig. 1C) [20,21]. Collectively, these factors synergistically impede molecular adsorption and activation at catalytic sites.

We hypothesize that a paradigm shift from thick, passive shells to atomically thin, defective semiconductor overlayers is required to address these intertwined challenges. Specifically, coating AuNRs with a rationally designed ultrathin semiconductor shell (such as metal sulfides) could fundamentally alter the electron–phonon coupling landscape. Such a configuration is anticipated to suppress electron–phonon interaction by reducing the phonon density of states and scattering cross-section, thereby prolonging the high-energy state lifetime of hot electrons while establishing spatiotemporally favorable conditions for their transfer to substrate molecules (Fig. 1D). Furthermore, the strong Au-S bonding interaction is anticipated to drive interface reconstruction, generating sulfur vacancies and exposed active metal sites that enhance N_2_ adsorption and subsequent N_2_ reduction. This dual-function design, targeting both the energy dissipation pathway and the electron-deficient site, represents a holistic strategy to overcome the fundamental bottlenecks in plasmonic N_2_ fixation. Although fabricating such a unique core–shell AuNRs-based heterostructure with an ultrathin semiconductor shell remains challenging, this strategy holds great potential for hot carriers’ effective utilization and N_2_ photofixation.

Herein, we present a rational design strategy to construct a unique core–shell heterostructure, termed GeS_x_-Au, which comprises an ultrathin semiconductor shell enriched with high-density sulfur vacancies. This tailored architecture is achieved through the atomic-scale reconstruction of molecular chalcogenide clusters (T2-Ge, (HPR)_4_[Ge_4_S_10_], PR = piperidine) as sacrificial precursors on the surface of Au nanorods. Experimental validation under NIR illumination demonstrates an unprecedented NH_3_ production rate of 1,225.6 μmol g^−1^ h^−1^ with an apparent quantum yield (AQY) of 1.23% at 880 nm for the GeS_x_-Au composite, surpassing all reported NIR-driven photocatalysts by 1 to 2 orders of magnitude and outperforming numerous UV/vis-light-activated systems. Through a combination of in situ spectroscopic techniques, transient absorption studies, and theoretical analysis, we decipher the synergistic mechanism: the GeS_x_ shell effectively suppresses phonon-mediated hot-carrier cooling, while the electron-deficient Ge sites lower the kinetic barrier for N_2_ activation via an associative distal pathway. This integration of long-lived energetic electrons with sulfur vacancy-mediated N_2_ anchoring establishes a synergistic catalytic mechanism for efficient N_2_ activation, markedly enhancing photocatalytic ammonia synthesis (Fig. 1E).

## Results

### Synthesis and structure

**Fig. 2.**
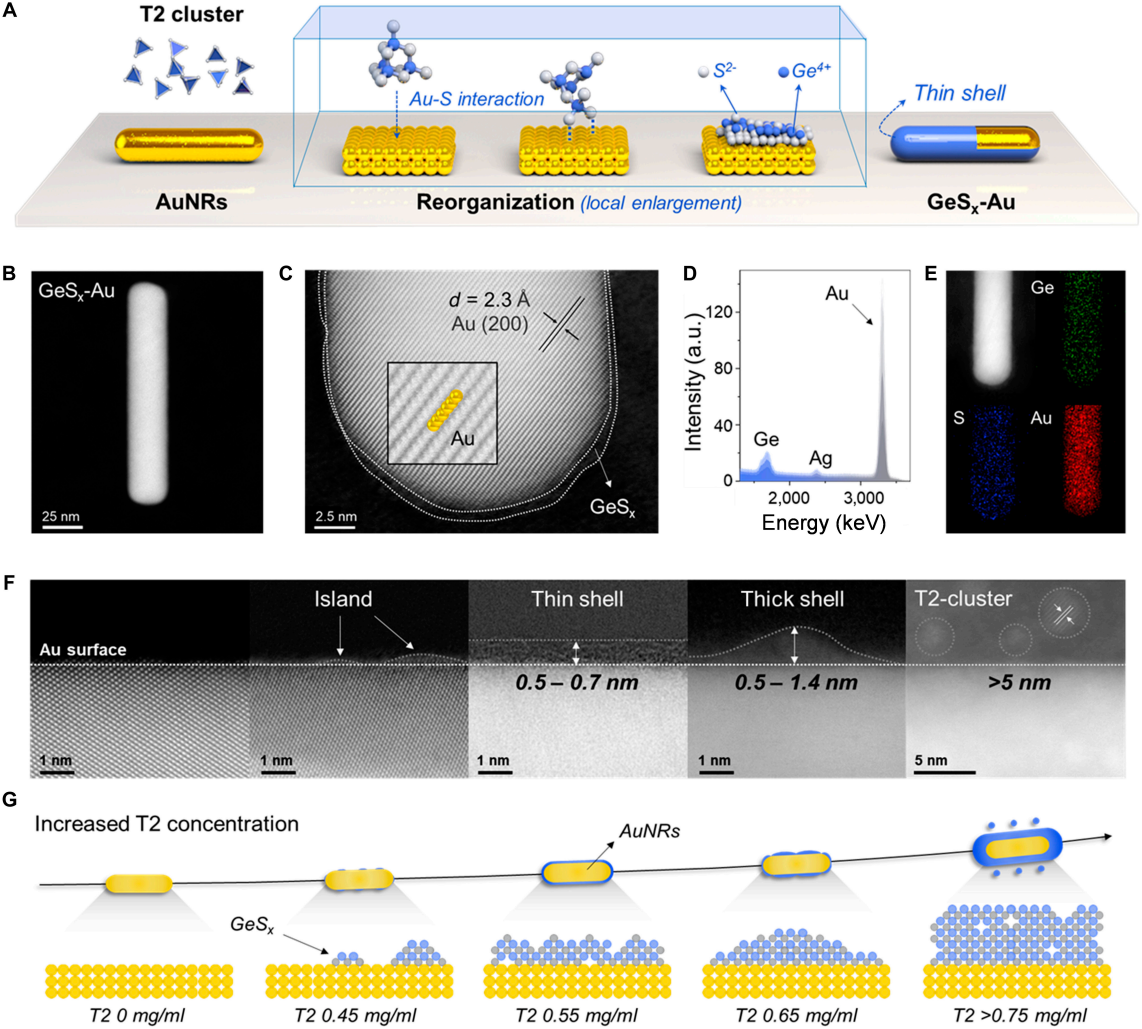
Synthesis and morphology of GeS_x_-Au nanostructures. (A) Schematic diagram of the synthesis process for GeS_x_-Au core–shell nanostructures. (B) AC-STEM image of the complete morphology for GeS_x_-Au and (C) localized magnification of the structure. (D) Low-energy ion scattering results. (E) Elemental distribution of GeS_x_-Au. (F) STEM images and (G) schematic illustration of the evolution of interfacial shell layers in GeS_x_-Au synthesized with different concentrations of T2-Ge precursors.

[Ge_4_S_10_]^4−^ clusters (denoted as T2-Ge, where “T” is short for tetrahedron and “2” indicates the number of metal layers along the tetrahedron edge) were synthesized via a facile and scalable method according to the previously reported protocol (Figs. S1 and S2) [22]. These clusters were subsequently prepared as aqueous sacrificial precursors at optimized concentrations. In parallel, AuNRs with a controllable size of approximately 20 × 80 nm were synthesized by a one-pot surfactant-directed growth approach (Fig. S3) [23]. Owing to the excellent aqueous dispersibility of both components, the GeS_x_-Au composite was efficiently fabricated by simply mixing the T2-Ge solution with AuNRs colloid under continuous stirring (Fig. 2A). Notably, the strong Au-S affinity may trigger partial sulfur depletion from T2-Ge during interfacial assembly, resulting in sulfur vacancies and the formation of an ultrathin amorphous GeS overlayer on AuNRs (vide infra). The monodisperse nature of T2-Ge cluster at low concentrations critically suppressed intermolecular aggregation, facilitating preferential Au-S bonding over cluster–cluster interactions.

Optical characterization revealed distinct spectral changes upon hybridization. Pristine AuNRs exhibited 2 characteristic LSPR absorption peaks: a transverse mode at 510 nm (visible range) and a stronger longitudinal mode at 810 nm (NIR region), consistent with their anisotropic morphology [5,10,11]. T2-Ge crystals displayed a colorless prismatic morphology with UV-exclusive absorption below 400 nm. The GeS_x_-Au nanocomposite inherits the plasmonic signature of AuNRs but showed a notable redshift of the longitudinal LSPR peak to 858 nm and a decrease in intensity, indicating an altered dielectric environment due to GeS_x_ coating (Fig. S4). The substantially broadened full width at half maximum highlights strong GeS_x_-Au interfacial coupling, which crucially governs subsequent catalytic processes [24,25].

The interfacial architecture and elemental distribution of the GeS_x_-Au composite were systematically investigated using aberration-corrected scanning transmission electron microscopy (AC-STEM) combined with spectroscopic mapping. Structural analysis showed that the GeS_x_-Au retained a rod-shaped morphology (aspect ratio ~4:1, Fig. 2B), analogous to pristine AuNRs. Atomic-resolution low-dose electron microscopy imaging resolved a continuous, ultrathin semiconductor shell uniformly encapsulating the AuNRs. Remarkably, single-atom-level imaging of Au atoms within the core was achieved, with lattice fringes corresponding to the intact Au (200) crystal planes (interplanar spacing: ~0.23 nm) [23]. The GeS_x_ shell thickness was precisely quantified as 0.5 to 0.7 nm (Fig. 2C), confirming conformal coating at the atomic scale. Complementary low-energy ion scattering (LEIS) analysis of the near-surface region detected pronounced Au and Ge spectral signatures, alongside trace Ag signals arising from residual Ag^+^ ions in the precursors of AuNRs [23]. The comparable intensity of Ag and Ge peaks substantially attenuated relative to the dominant Au signal-provides direct evidence for the ultralow-density attachment of GeS_x_ across the Au surface (Fig. 2D). Furthermore, spatially resolved elemental mapping demonstrates homogeneous dispersion of Au, Ge, and S across the hybrid architecture (Fig. 2E), unambiguously validating the successful bottom-up assembly of the composite with atomic-level interfacial precision.

To elucidate the influence of T2-Ge precursor concentration on the morphology of the GeS_x_ shell, systematic STEM analyses were conducted across a series of precursor loadings (0, 0.45, 0.55, 0.65, and 0.75 mg/ml; see the Supplementary Materials for details). In the absence of T2-Ge, AuNRs exhibit smooth surfaces and sharp edges (Fig. 2F). At 0.45 mg/ml, isolated island-like protrusions and surface roughening were observed, indicating incomplete and nonuniform GeS_x_ aggregation without full shell formation (Fig. 2G and Fig. S5). Uniform and continuous coverage was achieved at 0.55 mg/l, where an atomically thin GeS_x_ shell (0.5 to 0.7 nm) conformally coated the AuNRs (Fig. 2C and F). Increasing the precursor concentration to 0.65 mg/ml resulted in a thicker (0.5 to 1.4 nm) and increasingly heterogeneous shell (Fig. 2F and Fig. S6). At concentrations above 0.75 mg/ml, the thickness of the shell layer exceeded more than 5 nm and lattice streaks formed by aggregated T2-Ge were clearly observed (Fig. S7), at which time the T2 showed a stacking pattern on the outer surface rather than a reconfigure interface at the low concentration. These results underscore the critical role of precursor concentration in achieving an atomically precise core–shell architecture and confirm that the composite synthesized at 0.55 mg/ml T2-Ge—used as the reference material throughout this study—exhibits optimal structural characteristics.

### Surface structure information

**Fig. 3.**
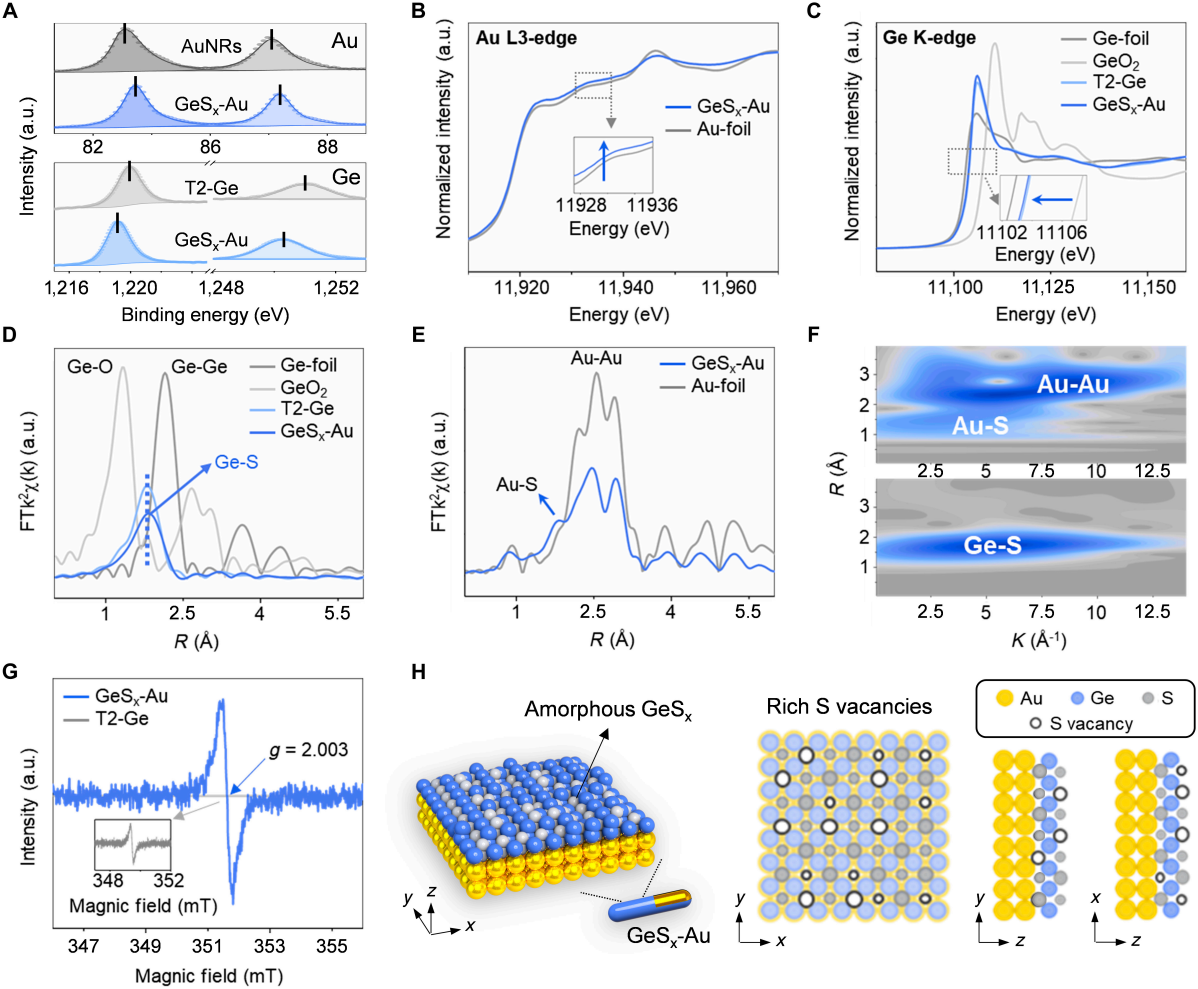
Structural characterization of GeS_x_-Au nanostructures. (A) XPS information of Au 4f and Ge 2p before and after composite formation. (B) Au L3-edge and (C) Ge K-edge XANES spectra of GeS_x_-Au and references. (D) FT-EXAFS spectra of Ge-foil, GeO_2_, T2-Ge, and GeS_x_-Au. (E) FT-EXAFS spectra of Au-foil versus GeS_x_-Au. (F) Wavelet transform of Au L3 edge and Ge K edge of GeS_x_-Au. (G) Comparison of EPR information of T2-Ge and GeS_x_-Au. (H) Schematic representation of the surface structure of GeS_x_-Au.

X-ray photoelectron spectroscopy (XPS) was conducted to investigate valence state evolution. In the Au 4f region (Fig. 3A), both AuNRs and GeS_x_-Au exhibited characteristic doublets corresponding to metallic Au (0), located at 83.8/87.6 eV and 84.1/87.8 eV, respectively. The slight positive shift (~0.2 eV) in the composite suggests modified electronic interaction at the interface. Similarly, the S 2p_3/2_ (162.0 eV) peaks in GeS_x_-Au demonstrated ~0.2 eV shifts toward higher binding energies compared to T2-Ge (161.8 eV, Fig. S8) [11,26,27], consistent with sulfur participation in Au-S bonds. The Ge 2p spectra maintained Ge^4+^ signatures in both T2-Ge (2p_1/2_ 1,219.9 eV, 2p_3/2_ 1,251.0 eV) and GeS_x_-Au (1,219.2 eV, 1,250.3 eV) (Fig. 3A), with a 0.7 eV negative shift indicating electron enrichment at Ge sites. These consistent binding energy shifts collectively support interfacial electron transfer from Au to Ge, mediated by sulfur bridges. Of particular note, the absence of lattice oxygen signals in O 1s spectra of GeS_x_-Au (Fig. S9) confirms the preservation of unoxidized Ge sites, where sulfur-depleted Ge centers serve as exposed active sites for N_2_ adsorption [28–30].

Furthermore, complementary synchrotron-based x-ray absorption fine structure (XAFS) studies provided atomic-level coordination insights. The Au L3-edge absorption edge of GeS_x_-Au shifted upward relative to Au foil (Fig. 3B), suggesting a slight positive charge on Au. Conversely, the Ge K-edge of GeS_x_-Au positioned between Ge foil and GeO_2_ (Fig. 3C), consistent with Ge^4+^ valence, corroborating the electron transfer from Au to Ge inferred from XPS [28,30]. Fourier-transform EXAFS (FT-EXAFS) spectra and wavelet transform analysis (Fig. 3D to F) further confirmed that the local coordination in GeS_x_-Au is dominated by Au-S and Ge-S bonds, with no discernible Ge-O or Ge-Ge contributions. Quantitative EXAFS fitting revealed an elongation of the average Ge-S bond from 2.25 Å (coordination number CN = 2.4) in T2-Ge to 2.32 Å (CN = 1.2) in GeS_x_-Au (Table S1), indicating a pronounced weakening of the Ge-S interactions due to competitive Au-S bonding. This structural rearrangement leads to the creation of coordinatively unsaturated, electron-deficient Ge sites, which synergistically enhance N_2_ activation through combined electronic and geometric effects [30] (detailed analysis and fitting results can be seen in Figs. S10 to S16 and Tables S1 and S2).

To further elucidate the structural evolution of T2-Ge on AuNR surfaces, electron paramagnetic resonance (EPR) spectroscopy was performed to probe sulfur vacancy defects in GeS_x_-Au. As depicted in Fig. 3G, GeS_x_-Au exhibited an intense EPR signal at *g* = 2.003, characteristic of sulfur vacancies [31,32], whereas pristine T2-Ge displayed negligible signals. Inductively coupled plasma (ICP) analysis revealed a marked reduction in the Ge/S ratio from ~2:5 in T2-Ge to ~1:1 in GeS_x_-Au (Table S3), confirming sulfur depletion during the interfacial restructuring process. This sulfur-deficient configuration generates abundant vacancy sites, which are well known to be critical for N_2_ chemisorption and activation, thereby implying the superior catalytic performance of GeS_x_-Au [26,27].

Based on the comprehensive evidence above, the proposed architecture for the unique core–shell heterostructure GeS_x_-Au catalyst has been developed (Fig. 3H): an ultrathin amorphous GeS_x_ layer with high sulfur vacancy density forms a close interface through Au-S bonds while retaining the electron-deficient state of the Ge sites. This architecture offers multiple synergistic advantages for photocatalytic N_2_ fixation: first, the electron-deficient and exposed Ge sites facilitate efficient N_2_ adsorption and activation; second, plasmon-derived hot electrons in AuNRs are rapidly extracted and channeled to these active surface sites, sustaining high catalytic reactivity; lastly, the abundant sulfur vacancies enhance the interfacial transfer kinetics of photogenerated hot carriers, thereby further augmenting catalytic efficiency. Collectively, these features of GeS_x_-Au catalyst spark our interest in NIR-driven N_2_ fixation.

### Reaction optimization

**Fig. 4.**
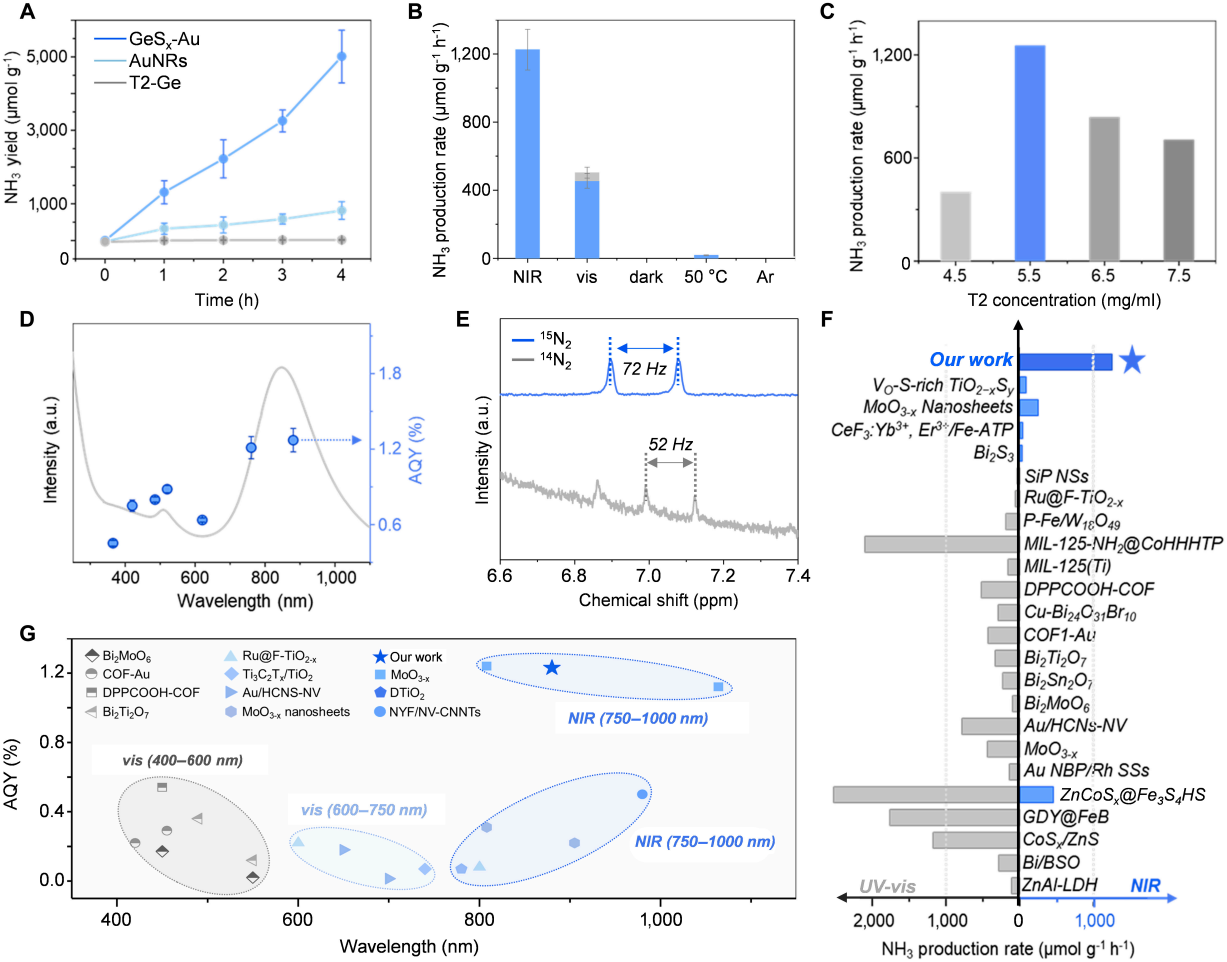
NIR-driven nitrogen fixation performance evaluation. (A) Catalytic performance in nitrogen fixation by AuNRs, T2-Ge, and GeS_x_-Au under NIR light (error bars represent standard error). (B) Nitrogen fixation by GeS_x_-Au under different conditions. (C) T2 concentration effect on NH_3_ production rates. (D) Photocatalytic quantum yield and absorption spectra of GeS_x_-Au. (E) ^1^H NMR spectra of the reaction mixture after photocatalysis with the GeS_x_-Au system using ^14^N_2_ or ^15^N_2_ as feeding gas. (F) Comparison of NH_3_ production rates of GeS_x_-Au with the reported photocatalysts. (G) AQY of GeS_x_-Au compared to reported photocatalysts under various excitation wavelengths.

The photocatalytic nitrogen fixation performance of GeS_x_-Au was systematically evaluated under NIR light irradiation (*λ* > 600 nm, 300 W Xenon lamp) in a water–acetonitrile (3:1 v/v) mixture, using methanol as a sacrificial agent at 25 °C for 4 h. Quantitative analyses employing the indophenol blue method (Figs. S17 and S18), Nessler’s reagent method (Fig. S19), ion chromatography (Fig. S20), and quantitative ^1^H NMR (Fig. S21) [29,30,33–37] revealed that the averaged NIR-driven ammonia production rate of GeS_x_-Au reached 1,225.6 μmol g^−1^ h^−1^ (Fig. 4A), representing a 6-fold enhancement compared with that of pristine AuNRs (Table S4). To rule out potential interference from cetyltrimethylammonium bromide (CTAB) and piperidine, ion chromatography was employed to analyze samples with different additives. Results showed that excess amounts of either additive led to a notable suppression in the measured ammonia production rate, likely due to site blocking or competitive adsorption. However, in our catalytic system, the actual ammonia yield exhibited only marginal deviation. This can be attributed to the repeated washing steps performed prior to the formation of the composite catalyst, which effectively minimized residual amounts of both substances (Fig. S20). No catalytic activity was detected for isolated T2-Ge clusters, consistent with their negligible NIR absorption. Comparative studies under visible-light irradiation (420 nm < *λ* < 600 nm) demonstrated diminished activity relative to NIR illumination (Fig. 4B and Table S5), aligning with the limited visible-light absorption of GeS_x_-Au. Intriguingly, while trace hydrazine hydrate was detected under full-spectrum/visible illumination (Figs. S22 and S23), no such by-product was observed under NIR excitation, highlighting the superior selectivity of NIR-driven catalysis. Although proton reduction is thermodynamically more favorable than N_2_ reduction, our highly sensitive detection methods, including isotope-labeling experiments with deuterated solvents and GC–MS analysis (Fig. S24), confirmed that H_2_ evolution in our photocatalytic system is extremely low (<0.1 μmol·g^−1^ h^−1^) and negligible compared to the NH_3_ production rate (Tables S6 and S7). This high selectivity for NH_3_ formation arises from several intertwined factors related to the catalyst’s active sites and the reaction medium. First, the engineered Ge sites adjacent to sulfur vacancies exhibit a strong preference for adsorbing and activating N_2_, which kinetically suppresses the competing adsorption and reduction of protons [38,39]. Second, the organic components in the solvent environment, along with the reaction pH, modulate proton availability and transport to the active surface, thereby further disfavoring the hydrogen evolution pathway [40]. Together, these effects reflect the successful design of a catalytic interface that is tailored to drive the more challenging nitrogen reduction reaction selectively under NIR illumination.

Reaction parameter optimization identified methanol as the optimal sacrificial reagent. Ion chromatography identified formate as the primary oxidation product, with no detectable nitrate or hydrogen evolution under any tested conditions (Fig. S25). Control experiments conducted under dark conditions or with N_2_ replaced by Ar yielded no product formation, confirming the photon-driven nature of the reaction and the inert role of acetonitrile (Table S5) [41,42]. At the same time, comparative experiments eliminated the interference of methanol, acetonitrile, and formate ions on the test results (Fig. S26). Furthermore, the reaction maintained considerable activity over 6 consecutive experimental cycles, demonstrating the operational stability of GeS_x_-Au (Fig. S27A). More importantly, post-reaction characterization revealed that the core–shell structure and elemental composition remained intact after cycling (Fig. S27B), confirming the catalyst’s structural robustness. In order to explore the origin of the excellent catalytic performance of GeS_x_-Au, analogous composites (P1-Zn-Au [43] and P1-Mn-Au [43]) derived from alternative chalcogenide cluster precursors were synthesized (Figs. S28 to S32). Precursor-to-AuNRs ratio optimization (0.55 mg/ml T2-Ge) disclosed a threshold for catalytic efficacy: excess T2-Ge induced particle aggregation and prolonged charge carrier transport distances, while insufficient loading limited active site density (Fig. 4C). To further explore the photocatalytic performance of GeS_x_-Au, wavelength-dependent photonic efficiency was systematically assessed across the UV to NIR spectrum. The pronounced correlation observed between photocatalytic quantum yield (AQY) and the characteristic absorption profile of GeS_x_-Au (Fig. 4D) underscores a direct photonic activation mechanism governed by intrinsic light-harvesting properties of the composite. Despite similarities in precursor size and elemental composition, these systems exhibited inferior activity, emphasizing the advantages of high Ge concentration and thin shell in N_2_ activation (Fig. S29).

Isotopic labeling experiments using ^15^N_2_ feedstock generated NH_3_ exhibiting ^1^H NMR doublet splitting (*J* = 72 Hz), distinctly different from the triplet splitting (*J* = 52 Hz) observed under ^14^N_2_, validating atmospheric N_2_ as the nitrogen source (Fig. 4E) [30,32]. Electrochemical impedance spectroscopy (EIS) analysis (Figs. S33 and S34 and Table S5) indicates that the GeS_x_-Au heterostructure features a highly uniform interface, with its double-layer capacitance soaring by more than 2 orders of magnitude relative to the T2-Ge precursor. This orders-of-magnitude improvement marks a qualitative leap in interfacial charge-storage capability, which constitutes the key electrochemical foundation for enhancing the efficiency of NIR photocatalytic nitrogen reduction—a process requiring the accumulation of multiple electrons [32]. Furthermore, under NIR irradiation, GeS_x_-Au generates a stable and stronger photocurrent response than T2-Ge (Fig. S35), directly reflecting its superior ability to produce and separate photo-generated charge carriers. Together, these electrochemical and photoelectrochemical characteristics corroborate more efficient charge separation and transfer in the heterostructure, which underpins its enhanced NIR-driven photocatalytic activity. Duty-cycle experiments with intermittent illumination (1 h light/dark intervals) reaffirmed that NH_3_ production is strictly light-dependent (Fig. S36). Despite differences in reaction conditions (notably the use of a CH_3_CN/H_2_O mixture with CH_3_OH as a sacrificial agent), the GeS_x_-Au catalyst exhibits exceptional NIR-driven performance. When benchmarked against leading NIR photocatalysts, it achieves a record-high NH_3_ production rate and an AQY that rivals the best reported values in this spectral regime. Notably, its efficiency is comparable to, or even surpasses, that of many systems operating under more energetic UV/vis (Fig. 4F and G and Tables S8 and S9). This remarkable performance highlights the effectiveness of our interface-engineering strategy in utilizing low-energy photons [31,44–49]. This work establishes a new paradigm for photocatalytic material design, demonstrating how GeS_x_-Au heterostructures synergistically integrate plasmonic field enhancement, defect-mediated N_2_ activation, and optimized charge carrier dynamics to achieve highly efficient nitrogen fixation under NIR illumination.

### Reaction mechanistic study

**Fig. 5.**
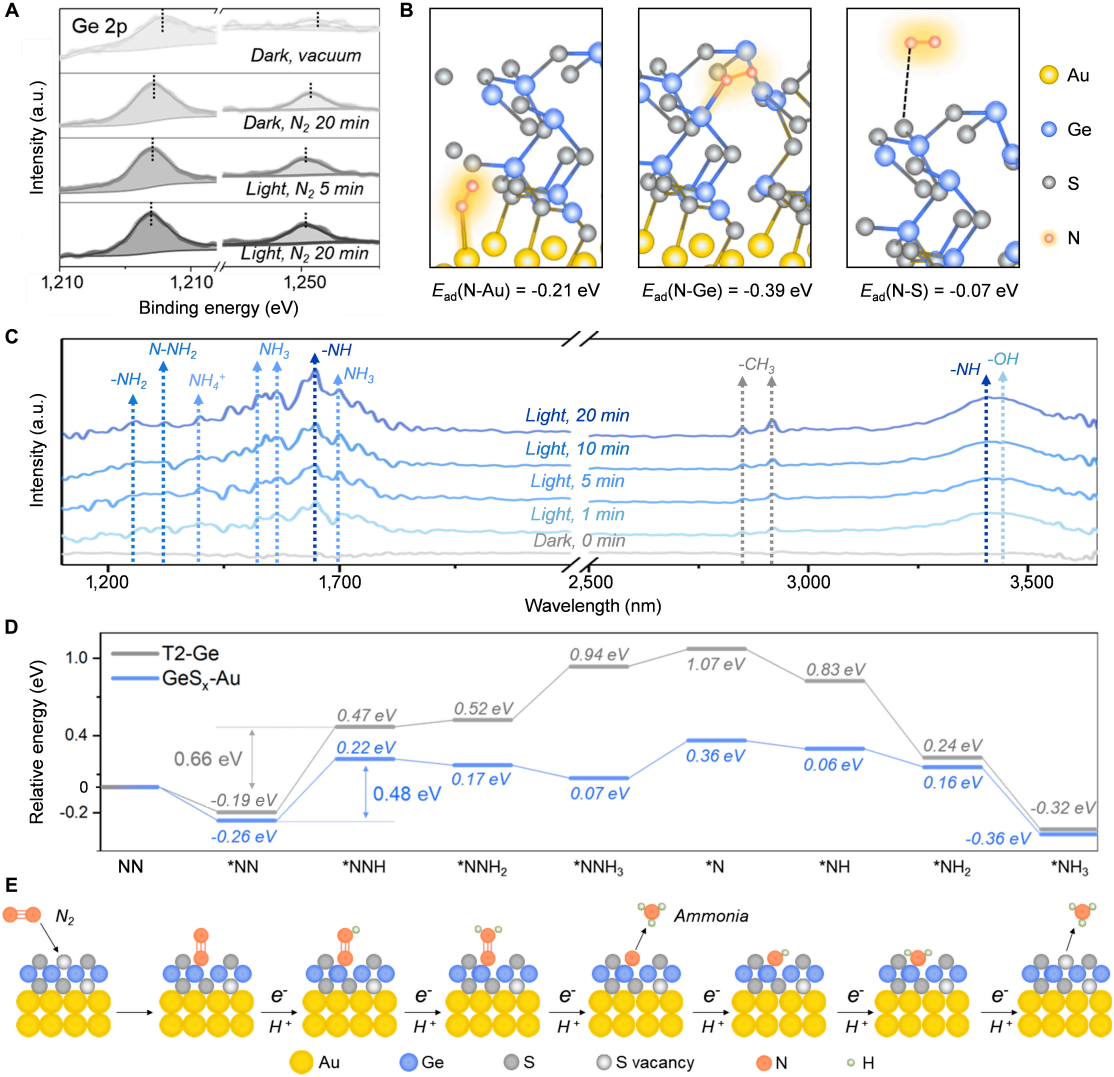
Mechanistic insights into photocatalytic nitrogen fixation. (A) In situ Ge 2p XPS spectra of GeS_x_-Au under dark vs. light conditions. (B) Calculation of the adsorption energy of N_2_ at different sites on the GeS_x_-Au surface. (C) In situ IR spectra of GeS_x_-Au with or without NIR light. (D) Gibbs free energy profiles of nitrogen reduction intermediates on T2-Ge and GeS_x_-Au. (E) Schematic diagram of the distal associative pathway of nitrogen fixation by GeS_x_-Au.

To quantitatively understand the enhanced N_2_ activation at the atomic level, we developed a theoretical model based on coordination environment and electronic structure considerations. The sulfur vacancies create under-coordinated Ge sites with reduced coordination number from 4 in pristine T2-Ge to approximately 2 to 3 in GeS_x_-Au, as evidenced by EXAFS fitting (CN = 1.2). These low-coordination sites exhibit higher surface energy and enhanced reactivity due to the presence of dangling bonds and localized electronic states near the Fermi level. Near-ambient pressure XPS under NIR illumination (*λ* > 700 nm) captured dynamic charge redistribution during N_2_ adsorption. Under dark conditions in an N_2_ condition, the Au 4f peaks (84.03 eV, 87.59 eV) remained nearly unchanged (Fig. S37 and Table S10), while the Ge 2p (2p_1/2_ 1,218.98 eV, 2p_3/2_ 1,249.85 eV) exhibited a concurrent 0.8-eV low-field shift (Fig. 5A and Table S9). This shift indicates interfacial electron donation from Ge to adsorbed N_2_, confirming preferential N_2_ anchoring at Ge sites near sulfur vacancies. Under NIR irradiation, the Ge 2p further negatively shifted (Δ = 0.3 eV) with no appreciable change in Au 4f, underscoring the role of GeS_x_ as the catalytic center for N_2_ activation and reduction [28]. Complementary theoretical calculations of N_2_ adsorption energies at various surface sites—including exposed Ge, S, and interfacial Au atoms—revealed markedly stronger adsorption on Ge sites (−0.39 eV) compared to Au (−0.21 eV) and S sites (−0.07 eV) (Fig. 5B and Fig. S38). This enhanced adsorption is attributed to the lower coordination number and higher surface energy of Ge atoms adjacent to sulfur vacancies, which facilitate enhanced interactions with N_2_ molecules. Together, the in situ XPS and theoretical adsorption energy provide consistent experimental and theoretical evidence identifying Ge as the primary site for N_2_ adsorption and activation [31].

Following the preliminary identification of activation sites, we pursued time-resolved in situ Fourier Transform infrared spectroscopy under NIR illumination (*λ* > 600 nm) to trace the evolution of intermediate species during the conversion of N_2_ to NH_3_ at Ge sites with enhanced temporal resolution (Fig. 5C). Post-illumination spectra exhibited progressive emergence of diagnostic vibrational modes: N–H stretching [ν(N-H)] at 1,646.94 and 3,409.58 cm^−1^, N–H bending [δ(NH_2_)] at 1,263.16 cm^−1^, N–N stretching [ν(N-NH_2_)] at 1,322.95 cm^−1^, and symmetric/asymmetric NH_3_ deformations at 1,562.08/1,525.44 cm^−1^. The 1,402.02 cm^−1^ signal confirmed the formation of NH_4_^+^, while features associated with methanol oxidation—C–H stretching vibrations [ν(C-H)] at 2,850.32 and 2,917.81 cm^−1^—were consistent with concurrent formate generation. Critically, the absence of N≡N stretching [ν(N≡N)] signal in the 1,900 to 2,300 cm^−1^ region rules out end-on N_2_ adsorption, providing strong evidence for a distal associative pathway. In contrast, the in situ FTIR spectrum recorded under an Ar atmosphere serves as a critical negative control, showing only a weak signal attributable to residual pyridinium salts (Fig. S39) and no detectable N–H or N–N vibrational modes. This result confirms that the rich set of nitrogenous intermediate signals observed under N_2_ atmosphere are exclusively derived from the activation and reduction of N_2_ gas, rather than from catalyst decomposition or residual contaminants. It provides direct spectroscopic evidence that N_2_ is essential for the observed reaction pathway and further validates the assignment of the reaction intermediates discussed above [28–31,41].

Gibbs free energy calculations were systematically performed to assess the activation energy barriers of intermediate species during the N_2_ reduction process on both T2-Ge and GeS_x_-Au surfaces, thereby elucidating kinetic distinctions before and after heterostructure formation. As depicted in Fig. 5D, the adsorption free energy of N_2_ on GeS_x_-Au was calculated as −0.26 eV, substantially more favorable than that on pristine T2-Ge (–0.19 eV), clearly demonstrating the enhanced reactivity of the GeS_x_-Au interface. Notably, the energy barrier required for the conversion of adsorbed N_2_ to the *NNH intermediate was reduced from 0.66 eV on T2-Ge to 0.48 eV on GeS_x_-Au, suggesting a more kinetically accessible pathway enabled by the hybrid architecture [29,31]. Moreover, the calculated free energy profiles for all key intermediates along the nitrogen fixation pathway were consistently lower on GeS_x_-Au compared to T2-Ge, providing theoretical evidence for the superiority of the GeS_x_-Au system in driving the nitrogen reduction reaction under kinetic control. Collectively, these results enable us to propose an integrated reaction mechanism (Fig. 5E) governed by a distal associative pathway on the GeS_x_-Au surface. Specifically, sulfur vacancy sites (associated with Ge centers) facilitate the sequential hydrogenation of adsorbed N_2_ molecules, driving their progressive transformation into NH_3_ intermediates that ultimately desorb from the active sites. The mechanistic trajectory underscores the critical role of vacancy-engineered coordination environments in stabilizing kinetically challenging reduction steps.

### Photophysical mechanistic study

**Fig. 6.**
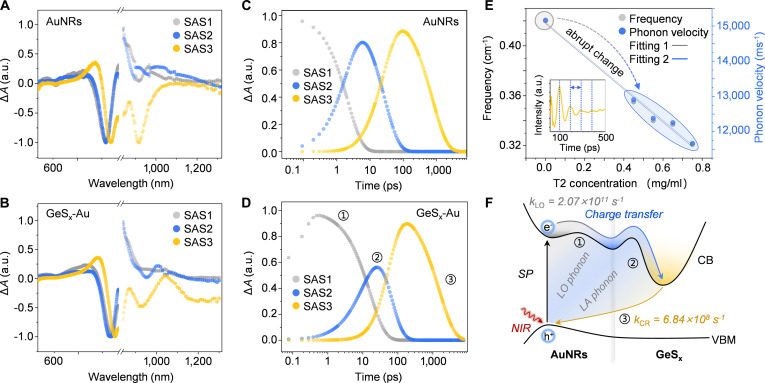
Charge transfer dynamics in pure AuNRs and GeS_x_-Au hybrids. Deconvoluted species associate spectra of (A) AuNRs and (B) GeS_x_-Au. The population dynamics of (C) AuNRs and (D) GeS_x_-Au. (E) Frequency and photon velocity trend of GeS_x_-Au with different T2 concentrations. The inset demonstrates the oscillation of the signal. (F) Schematic diagram demonstrating the hot-carrier cooling, charge separation, and charge recombination process.

Transient absorption (TA) spectroscopy was then performed on GeS_x_-Au hybrids and AuNRs under 800 nm pump excitation to clarify their photodynamics (Figs. S40A and S41B). The 2 systems exhibited closely analogous spectral evolution across the visible region. For AuNRs, positive excited-state absorption (ESA) signals were observed at 480, 600, 820, and 1,000 nm, accompanied by a photobleach (PB) band centered at 780 nm, consonant with their intrinsic absorption profiles. Progressive bathochromic shifting around the 600 nm ESA and 780 nm PB signals suggests hot-carrier sequential relaxation via excited-state energy dissipation pathways [25,47,48]. GeS_x_-Au displayed similar transient spectral evolution with a marginal bathochromic shift relative to AuNRs, in agreement with its steady-state absorption, confirming that the ultrathin GeS_x_ overlayer preserves the plasmonic excitation character of the AuNR core. To detail their kinetics, global analysis of transient spectra resolved a 3-species sequential model for both systems (Fig. 6A to D). As illustrated in Fig. 6A for the first species of AuNRs, denoted as SAS1 (species-associated spectra 1), the entire PB signal from visible to NIR is displayed, indicating that a fast cooling process with a rate constant exceeding 4.61 × 10^11^ s^−1^, likely attributable to longitudinal optical (LO) phonon scattering (Table S11). In contrast, for GeS_x_-Au, the evolution of the bleaching redshift process caused by the rapid relaxation of hot electrons can be clearly traced, proving the prolongation of the hot electron relaxation process (Fig. 6B), with a reduced LO phonon scattering rate of 2.07 × 10^11^ s^−1^. The observed reduction in the LO phonon scattering rate (from 4.61 × 10^11^ to 2.07 × 10^11^ s^−1^) directly evidences the suppression of high-frequency phonon modes, which are the primary channel for hot-carrier energy dissipation in plasmonic metals. This phenomenon can be understood within the framework of electron–phonon coupling: the amorphous, ultrathin GeS_x_ shell introduces structural disorder that reduces the phonon density of states and acts as a kinetic bottleneck for energy relaxation, thereby favoring the charge transfer pathway over thermalization.

Meanwhile, the SAS2 exhibits a slower rate to dissolve in GeS_x_-Au (1.03 × 10^10^ s^−1^) relative to bare AuNRs (3.70 × 10^10^ s^−1^), maintaining a similar longitudinal acoustic (LA) phonon scattering pattern (Fig. 6C and D and Table S11). We proposed that the first 2 species in GeS_x_-Au likely involve 2 competing pathways: phonon scattering of hot electrons versus interfacial charge transfer. Upon anchoring GeS_x_ to the Au surface, the pronounced S-Au bonding interaction renders the transfer pathway predominant over the phonon scattering channel. Consequently, although SAS2 manifests a comparable LA phonon scattering signature, a substantial fraction of hot electrons migrates to the GeS_x_ surface for subsequent catalytic reactions, thereby mitigating energy dissipation via phonon scattering in the transfer channel. Eventually, AuNRs recover to the ground state with a derived kinetic of 1.30 × 10^9^ s^−1^ (Table S11). In contrast, SAS3 of GeS_x_-Au not only demonstrates bathochromic shift as we stated, but also presents a 2 times slower kinetics due to the charge recombination pathway channel emerged [24,25,49], reaching *k*_CR_ = 6.84 × 10^8^ s^−1^. In short, the GeS_x_ deposition strategy greatly prolongs the hot electron lifetime, thereby improving the utilization efficiency of hot electrons via charge transfer pathway (Fig. 6F). Beyond the electron dynamics, phonon dynamics can also reflect the excited-state energy deactivation. We extracted the frequency from the oscillation period (*T*) of transient absorption spectra and calculated their phonon velocity (*v*) using Eq. (1), where *λ* is the probe wavelength and *n*(*λ*) is the corresponding refractive index:

$$T\left(\lambda \right)=\frac{\lambda }{2vn^{\prime }{\left(\lambda \right)}^{}}$$ (1)

Transient absorption spectroscopy with GeS_x_-Au enabled mapping of phonon velocity and frequency (Fig. 6E) [50–52]. The GeS_x_ deposition reduces phonon velocity, slowing heat dissipation and thereby promoting the participation of hot electrons in the charge transfer channel. Furthermore, since the hot-electron cooling rate $\tau_{\text{cooling}}^{-1}$ is proportional to the square of the phonon frequency (*ω*), as described by Eq. (2):

$$\tau_{\text{cooling}}^{-1}\propto \omega^{2}$$ (2)

the decreased phonon frequency in GeS_x_-Au results in a longer hot-electron lifetime within the phonon scattering channel, consistent with the global fitting analysis. In essence, in the GeS_x_-Au sample, both the thermal transport and phonon scattering channels are suppressed, allowing the charge transfer channel to dominate (Fig. 6F) [50,53–55]*.* These synergistic effects collectively extend hot electron lifetimes, elevating the spatiotemporal probability for electron transfer to nitrogen adsorbates at sulfur vacancy anchoring sites. This prolonged retention of electrons in high-energy states provides a rational explanation for the orders-of-magnitude enhancement in catalytic activity observed in the GeS_x_-Au system.

## Discussion

**Fig. 7.**
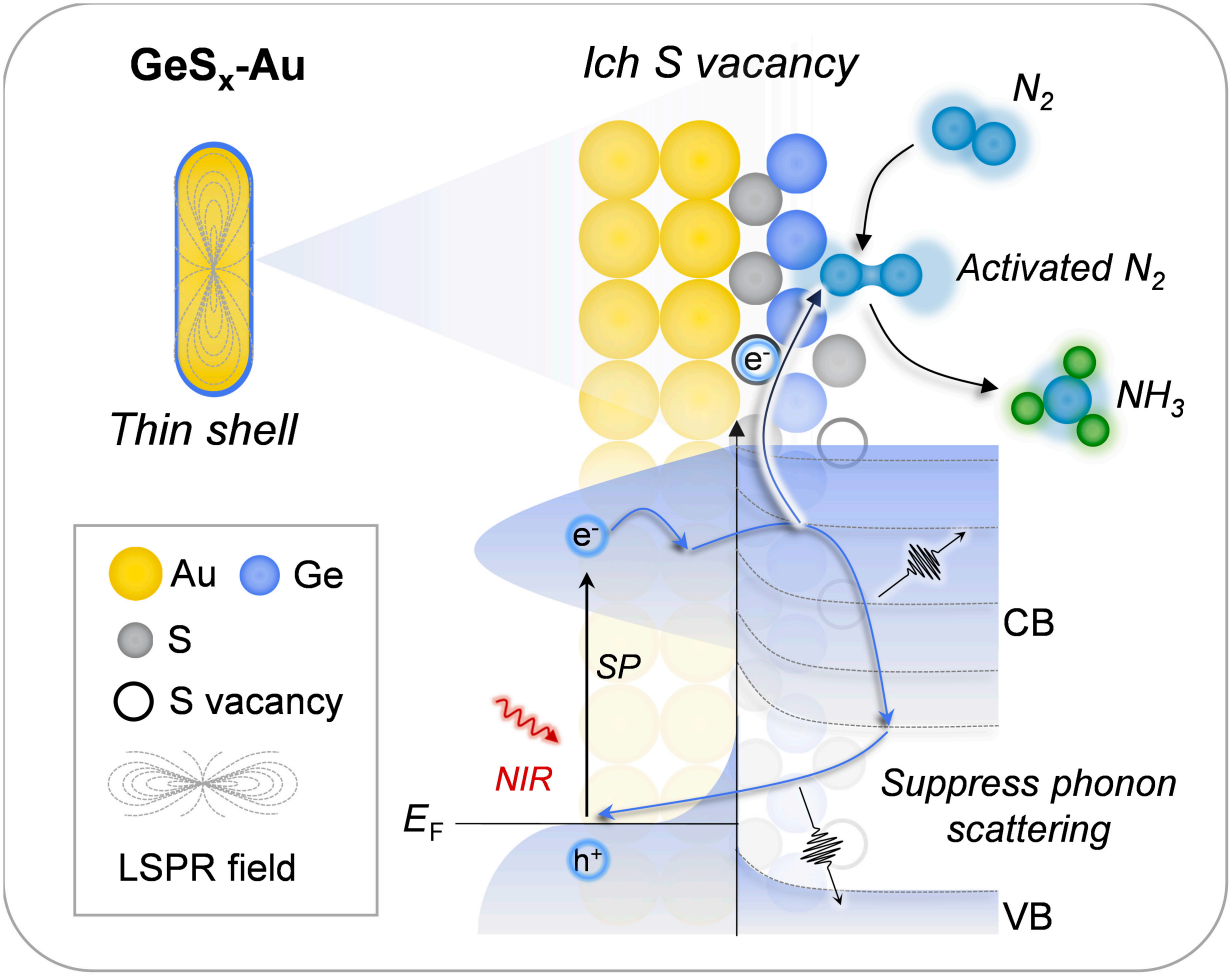
GeS_x_-Au promotes nitrogen activation through a synergistic sulfur vacancy and phonon scattering suppression mechanism.

In this study, we have demonstrated a rational materials design strategy that tackles the core issue of energy loss in plasmonic photocatalysis. By constructing a GeS_x_-Au heterostructure with an atomic-scale shell and engineered sulfur vacancies, we achieved a simultaneous suppression of phonon scattering and creation of highly active sites for N_2_ activation. This synergy, definitively proven by transient absorption spectroscopy, enables record-breaking efficiency for NIR-driven nitrogen fixation. As demonstrated in Fig. 7, our findings reveal a synergistic catalytic cycle on GeS_x_-Au: (a) The atomically thin shell engineers the energy dissipation pathway by suppressing phonon scattering (TA evidence), thereby prolonging the lifetime of plasmonic hot electrons. (b) Concurrently, the sulfur vacancies engineer the catalytic interface by creating electron-deficient Ge sites (XPS/XAFS evidence) that serve as preferential centers for N_2_ adsorption (in situ XPS evidence) and activation. (c) These long-lived, energetic electrons are then efficiently channeled to the adsorbed N_2_, driving its stepwise hydrogenation via a distal associative pathway (in situ FT-IR evidence) with reduced kinetic barriers. Our findings establish a general materials design principle for plasmonic photocatalysis: the strategic integration of an atomically thin, vacancy-rich semiconductor shell enables simultaneous control over both energy dissipation pathways and molecular activation sites. This dual-control strategy addresses the fundamental challenge in plasmonics—the mismatch between ultrafast hot-carrier generation and slow catalytic turnover—by creating a synergistic interface where electronic, vibrational, and catalytic processes are optimally coupled. This model of concurrent energy- and matter-pathway engineering provides a new design principle for overcoming fundamental limitations in plasmon-mediated catalysis. Our approach—using molecular clusters to precisely engineer metal-semiconductor interfaces—opens a general pathway for designing advanced photocatalysts that can fully exploit the low-energy photons that constitute the majority of the solar spectrum, with potential applications in other challenging photocatalytic reactions such as CO_2_ reduction and methane conversion.

## Materials and Methods

### Preparation of T2-Ge crystals

T2-Ge was synthesized via a solvothermal reaction involving 100 mg of GeO_2_, 100 mg of sulfur powder, and 2.5 ml of piperidine (PR) at 190 °C for 6 days. Colorless single crystals of [Ge_4_S_10_]·(H^+^-PR)_4_ (T2-Ge) were obtained with a yield of ~95% (based on GeO_2_) [22].

### Preparation of P2 crystals

P2 crystals with a molecule format of {[Cu_5.88_In_11.9_Sn_8.22_S_44_]·(template)} were synthesized according to the literature. CuI (40 mg, 0.21 mmol), In powder (114 mg, 1 mmol), Sn powder (60 mg, 0.5 mmol), and S powder (224 mg, 7 mmol) were added to a mixed solution of H_2_O, (R)-(+)-2-amino-1-butanol (AB), and 1,5-diazabicyclo[4.3.0]non-5-ene (DBN) in a 1:1:3 volume ratio (5 ml). After stirring at room temperature for half an hour, the mixture was heated at 170 °C for 7 days. The resulting mixture was then cooled to room temperature. Black–red crystals of P2 were obtained (80% yield, based on In), washed with anhydrous ethanol, and dried under vacuum [36].

### Preparation of P1-Zn crystals

A mixture of S powder (180.0 mg, 5.625 mmol), K_2_S_x_ powder (90.0 mg), Zn(Ac)_2_·2H_2_O (60.0 mg, 0.27 mmol), GeO_2_ (50.0 mg, 0.48 mmol), DBN (2.0 ml), and H_2_O (1.0 ml) was placed in a sealed autoclave and stirred for 30 min. Then, the vessel was heated at 200 °C for 8 days. After cooling to room temperature, a large amount of light pink cubic crystals of K_6_[Ge_4_Zn_4_S_13_(OH)_4_](C_7_H_12_N_2_)0.5(H_2_O)_x_ was obtained (55% yield, based on Zn) [35].

### Preparation of P1-Mn crystals

A mixture of S powder (180.0 mg, 5.63 mmol), K_2_S_x_ powder (150.0 mg), GeO_2_ (50.0 mg, 0.48 mmol), Zn(Ac)_2_·2H_2_O (35.0 mg, 0.16 mmol), Mn(Ac)_2_·4H_2_O (27.0 mg, 0.11 mmol), DBN (2.0 ml), and H_2_O (1.0 ml) was placed in a sealed autoclave and stirred for 30 min. The vessel was heated at 200 °C for 8 days. A large amount of laurel-green cubic crystals of K_6_[Ge_4_Mn_1.2_Zn_2.8_S_13_(OH)_4_](C_7_H_12_N_2_)0.5(H_2_O)_x_ was obtained (36% yield, based on Mn) [35].

### Preparation of AuNRs

AuNR dispersion was synthesized according to the literature protocol. Solutions of CTAB and sodium oleate (200 mM) were pre-prepared and heated to 70 °C with stirring until complete solute dissolution, and then cooled to 30 °C before use. In a well-washed vial, 2.4 ml of CTAB, 0.625 ml of sodium oleate, and 1.925 ml of deionized water were added and stirred at 500 rpm to mix thoroughly. Subsequently, 5 ml of HAuCl_4_ solution (2 mM), 240 μl of AgNO_3_ solution (4 mM), 50 μl of HCl (11.8 M), and 75 μl of ascorbic acid solution (85.8 mM) were sequentially added. After stirring at 1,200 rpm for 5 min, 7.5 μl of freshly prepared ice-cold NaBH_4_ aqueous solution (10 mM) was rapidly injected into the mixture, and stirring was immediately stopped. The mixture was then kept at 30 °C for 4 h. The mixture was centrifuged at 9,000 rpm for 30 min to separate the components. The supernatant was discarded, and the precipitate was resuspended in 10 ml of water to obtain the AuNR dispersion [23].

### Preparation of GeS_x_-Au

T2-Ge clusters (11 mg) were dispersed in 10 ml of deionized water and stirred for 1 min to obtain a clear and transparent solution. Five milliliters of the as-prepared AuNRs dispersion was then concentrated to 0.25 ml, and 0.7 ml of T2-Ge aqueous solution was added. The mixture was stirred for 4 h, followed by centrifugation at 12,000 rpm for 5 min. The supernatant was removed, and the remaining solid was dried in a vacuum oven at 30 °C for 8 h to obtain approximately 0.63 mg of GeS_x_-Au (black powder) (Au:Ge:S ≈ 2,000:1:1). A similar synthesis scheme was used for the other ratios, and only the concentration of the T2 precursor dispersion was adjusted to 0.45, 0.65, and 0.75 mg/ml, respectively.

## Data Availability

The data that support the findings of this study are available within the paper and the Supplementary Materials or are available from the corresponding authors upon request.
