# Supplementary material for: Atomic-Shell Engineering of GeSx-Au Nanorods Synergistically Suppresses Phonon Scattering and Activates N2 for Near-Infrared-Driven Ammonia Synthesis
Source: Research (Wash D C). 2026 Feb 25;9:1169. doi: 10.34133/research.1169 (PMC12932937; doi:10.34133/research.1169)
Supplement: Supplementary 1 — Supplementary Text Figs. S1 to S40 Tables S1 to S11 [file research.1169.f1.docx]

Supporting Information

**Atomic-Shell Engineering of GeS_x_-Au Nanorods Synergistically Suppresses Phonon Scattering and Activates N_2_ for Near-Infrared-Driven Ammonia Synthesis**

*Jia-Xing Liu, Jin-Guan Liang, Xu-Hang Zhong, Bingzhe Wang*, Shang-Fu Yuan*, Zhiling Yu, Yu Han, Tao Wu* and Dan Li*

**Contents**

**1.** Physical measurements

**2.** General catalytic procedure

**3.** Details of the synchrotron fit

**4.** Computational details

**5.** Complementary characterization

**6.** References

**1. Physical measurements**

Powder X-ray diffraction (PXRD) was performed on a Rigaku Ultima IV diffractometer with experiment parameters of 40 kV, 30 mA using Cu-Kα radiation (λ = 1.5418 Å) with a scan speed of 0.5 °/min and a step size of 0.02°. The morphology and elemental distribution were studied by C_S_-corrected STEM (Spectra 300) equipped with a focused ion beam electron beam microscope (FEI Helios Nanolab G3 CX). Gas chromatography mass spectrometer (GC-MS, Agilent 7890B) was used to detect the mass-to-charge ratio information of the products. Liquid-phase ^1^H and ^13^C NMR spectra were collected on a Bruker Biospin Advance spectrometer (400 MHz). UV-vis diffuse reflection spectra were obtained on Agilent Cary 4000 with BaSO_4_ as the reference. Ultraviolet photoelectron spectroscopy (UPS) was collected on a Thermo ESCALAB 250XI system. The experiments of femtosecond transient absorption (fs-TA) spectra were achieved on the Ti/sapphire laser system (Coherent, Astrella-Tunable-F-1k) and the fs-TA system (Ultrafast Systems, Helios Fire). The repetition rate of the amplifier is 1 kHz. The output power of 800 nm, which has a pulse width of <100 fs, is about 7.04 W. The broadband probe pulses (500-1300 nm) were generated by focusing a small portion of the fundamental 800 nm laser pulses into an Al_2_O_3_ plate. Mott-Schottky curves were tested on an electrochemical workstation. Electron paramagnetic resonance (EPR) was performed using a Bruker A300, testing the S vacancy, with excitation light at 808 nm, under a nitrogen atmosphere, and an illumination time of 10 min. Finite-difference time domain (FDTD) method was applied with perfectly matched layer conditions in the x, y, and z directions. The simulation domain was discretized into 0.5 nm mesh grids. Importantly, a total field scattered field plane wave with a wavelength of 780 nm is used as an excitation light source incident perpendicularly to the nanorods along the z-direction. Cs-corrected STEM measurements were performed on a Thermo Scientific Spectra 300 (S)TEM at an accelerating voltage of 300 kV.

**2.** **General catalytic procedure**

**2.1 Standard curve fitting (Indophenol blue colorimetric method)**

A 50 mL aqueous solution of 0.625 M NaOH was prepared. 2.49 g salicylic acid and 2.2 g sodium citrate were dissolved in the solution under vigorous stirring to form Colorimetric Reagent A. Meanwhile, a 10 mg/mL aqueous solution of sodium nitroprusside (Reagent B) was prepared and stored in a light-protected container. For calibration, five ammonium sulfate ((NH_4_)_2_SO_4_) standard solutions (0.02, 0.04, 0.06, 0.08, and 0.1 M, each containing 2 mg/L Na_2_SO_4_) were prepared in 2 mL aliquots. To each solution, 75 μL of 0.1 mol/L NaClO standard solution, 1.5 mL of Reagent A, and 0.5 mL of Reagent B were added sequentially. The mixtures were thoroughly stirred and then incubated in the dark for 2 h. After incubation, the absorbance of the solutions was measured at 658 nm using UV-vis spectrophotometry. A linear standard calibration curve was constructed by analyzing the absorbance-concentration relationship, ensuring an R² ≥ 0.995.

**2.2 Standard curve fitting (Nessler reagent method)**

A 500 g/L aqueous solution of sodium potassium tartrate was prepared and boiled for 30 min to eliminate potential NH_3_ impurities, serving as the metal ion masking agent. Subsequently, 7.013 g of KOH and 3.639 g of potassium tetraiodomercurate (II) (K_2_HgI_4_) were ultrasonically dissolved in 100 mL of ultrapure water to formulate the color-developing agent. Five standard (NH_4_)_2_SO_4_ aqueous solutions were prepared with concentrations of 0.02, 0.04, 0.06, 0.08, and 0.10 M in 2 mL aliquots. To each solution, 0.5 mL of the masking agent and 0.5 mL of the color-developing agent were added. After thorough mixing, the mixtures were allowed to react in darkness for 10 min, followed by UV-vis spectrophotometric measurement of the absorbance at 420 nm to establish a linear calibration curve (R² ≥ 0.995).

**2.3 Standard curve fitting (Ultraviolet colorimetry)**

A colorimetric reagent was prepared by dissolving 1 g of p-dimethyl-aminobenzaldehyde in a mixture of 6 mL concentrated HCl and 50 mL ethanol under vigorous stirring. Four standard hydrazine (N_2_H_4_) aqueous solutions (4, 5, 8, and 10 mg/L) were prepared in 2 mL aliquots. To each solution, 2 mL of the colorimetric reagent was added. The mixtures were allowed to react in the dark at ambient temperature for 30 min. The absorbance of the solutions at 458 nm was measured using UV-vis spectrophotometry to establish a linear calibration curve (R² ≥ 0.995).

**2.4 Procedure A for photocatalytic nitrogen fixation**

A 20 mL reaction vessel was charged with 0.6 mg of dried **GeSₓ-Au** catalyst, 6 mL mixed solvent (H_2_O/acetonitrile = 5:7 v/v), 1 mL methanol, and 14 mg Na_2_SO_4_. The mixture was stirred for 1 min to ensure redispersion of the catalyst. Three freeze-pump-thaw cycles were performed under N_2_ atmosphere to purge dissolved oxygen, with temperature maintained at 25 °C using a recirculating chiller. The system was irradiated for 4 h using an Xe lamp (spectral range: 600-2200 nm). After the reaction, gas-phase products (1 mL) were sampled and analyzed using gas chromatography (GC). The solution was centrifuged at 12,000 rpm for 30 min to pellet the catalyst. 6 ml of supernatant was filtered through a 0.22 µm membrane and rotary evaporated to dryness. The residue was reconstituted in 2 mL deionized water, and mixed with indophenol blue reagent for a 2 h dark reaction. Absorbance was measured at λ = 625 nm (or 420 nm for Nessler reagent method) and the NH_4_^+^ concentration was calculated using the pre-established calibration curve. The preliminary yield was derived via Equation (1), and the mean of triplicate measurements was reported as the final yield.

$$PR_{{NH}_{3}}(\mu mol g^{-1} h^{-1})=\frac{C_{{NH}_{4}^{+}}\times V}{m_{cat.}\times t} (1)$$

Following the same procedure, the supernatant (6 mL) was collected for N_2_H_4_ detection. The supernatant was dried, reconstituted in 2 mL deionized water, and treated with a hydrazine-specific chromogenic reagent for 30 min in the dark. Absorbance was measured at λ = 458 nm and the N_2_H_4_ concentration was calculated using the pre-established calibration curve. The yield of N_2_H_4_ was calculated via Equation (2), with results averaged from triplicate measurements.

$${PR}_{{N_{2}H}_{4}}(\mu mol g^{-1} h^{-1})=\frac{C_{N_{2}H_{4}}\times V}{m_{cat.}\times t} (2)$$

**2.5 Procedure B: Nitrogen fixation kinetics experiment**

Kinetic studies were conducted following catalytic Program A, with sampling at 1, 2, 3, and 4 h intervals. At each time point, 0.2 mL of reaction solution was filtered through a 0.22 µm membrane and dried via rotary evaporation. The residue was reconstituted in 2 mL deionized water. NH_4_^+^ and N_2_H_4_ concentrations were quantified using the previously described indophenol blue (λ = 625 nm) or hydrazine-specific (λ = 458 nm) chromogenic protocols, respectively. Nitrogen fixation yields were calculated via Equation (3), incorporating dilution factors and reaction volumes for normalization. Time-dependent yield profiles were plotted to derive kinetic parameters. All measurements were performed in triplicate, with mean values reported as final data points.

$$P_{{NH}_{3}}(\mu mol h^{-1} g^{-1})=\frac{C_{{NH}_{4}^{+}}\times V}{m_{cat.}} (3)$$

**2.6 Procedure C: Light on-off experiment for nitrogen fixation**

Cyclic light-switching experiments were performed following catalytic Program A. The system was irradiated with an Xe lamp (600-2200 nm) for 1 h, followed by 1 hour of reaction in the dark with stirring. This 1-hour light → 1-hour dark cycle was repeated four times, making a total of 8 hours. Samples were collected at the transition between each light and dark phase. A 0.1 mL sample was filtered through a 0.22 µm membrane, immediately processed by drying via rotary evaporation, reconstituted in 2 mL deionized water, and analyzed for NH_4_^+^ concentration (λ = 625 nm). Nitrogen fixation yields were calculated using Equation (3), normalized to the sampling volume. Yield-time profiles were plotted, with all procedures executed in triplicate under temperature-controlled (25 °C) conditions.

**2.7 Procedure D: Cycle experiment for nitrogen fixation**

The cyclic experiment followed catalytic Program A. After analyzing the supernatant, the residual liquid was decanted. The residue was washed with deionized water, and vacuum-dried at 30 °C for 8 h to recover the catalyst. The regenerated system was then reused and analyzed in the same way to assess catalytic performance. Yields from each cycle were compared to evaluate catalyst recyclability.

**2.8 Photocatalytic quantum yield measurement**

The wavelength-dependent photopower test followed catalytic Program A, using a sealed quartz reactor on a photocatalytic platform. Reactions were carried out under monochromatic LED irradiation at 365, 420, 485, 520, 620, 760, and 880 nm. A suspension was prepared by adding 0.6 mg catalyst, 6 mL H_2_O/CH_3_CN (5:7, v/v), 1 mL methanol, and 14 mg Na_2_SO_4_, then stirring for 1 min. The system was purged with N_2_ for 30 min to remove oxygen. Before irradiation, photopower density (mW/cm²) was measured at the sample port using a calibrated photopower meter. The reactor was illuminated for 1 h, and photopower was measured again to calculate pre-/post-reaction averages. Post-reaction, nitrogen fixation yields were quantified via indophenol blue method at λ = 655 nm, and apparent quantum yield (AQY) was calculated using Equation (4).

$$AQY\left( \% \right)=\frac{6\times n\times N_{A}}{{W\times A\times t}/{h\times v}} (4)$$

*n*: the molar number of generated NH_3_, *W*: the total input power, *A*: the irradiation area, *t*: reaction time, *v*: the incident light frequency, *N_A_* and *h* are the Avogadro constant and Planck constant^1-3^.

**2.9** **Isotope tracking experiment**

Catalytic tests were conducted under pure ^14^N_2_ or ^15^N_2_ atmospheres according to Program A. After the reaction, the solutions were processed individually: centrifuged at 12,000 rpm for 30 minutes to remove the catalyst, and 6 mL of the supernatant from each gas condition was collected. The solution was filtered through a 0.22 μm membrane and acidified to pH < 2 with 0.5 M HCl. The acidified samples were then concentrated to 1.0 mL via vacuum rotary evaporation. For ^1^H NMR analysis, 0.8 mL of the concentrate from each isotopic condition was mixed with 0.4 mL D_2_O (99.9% deuterium). For quantitative NMR experiments, 1 mL of the reaction supernatant is taken for calibration. The remaining test methods are the same as described above. In addition, 5 mg of maleic acid needs to be added as an internal standard. Finally, the ammonia yield is calculated according to formula (5).^7^

$$m_{{NH}_{4}^{+}}=M_{{NH}_{4}^{+}}\times\frac{m_{std}}{M_{std}}\times\frac{A_{{NH}_{4}^{+}}}{A_{std}}\times\frac{n_{std, H}}{n_{{NH}_{4}^{+},H}} （5）$$

**3. Details of the synchrotron fit**

Data reduction, data analysis, and EXAFS fitting were performed using the Athena and Artemis programs of the Demeter data analysis packages^4^, which employ the FEFF6 program^5^ to fit the EXAFS data. The energy calibration of the sample was conducted through a standard Fe foil, which was simultaneously measured as a reference. A linear function was subtracted from the pre-edge region, then the edge jump was normalized using Athena software. The χ(k) data were isolated by subtracting a smooth, third-order polynomial approximating the absorption background of an isolated atom. The k^2^-weighted χ(k) data were Fourier transformed after applying a Hanning window function (Δk = 1.0).

**3.1 Data analysis for Au**

For EXAFS modeling, the global parameters—coordination number (CN), bond distance (R), Debye-Waller factor (σ^2^), and energy shift (ΔE_0_)—were determined by nonlinear fitting through least-squares refinement of the EXAFS equation to the Fourier-transformed data in R-space using Artemis software. EXAFS of the Au foil was fitted and the obtained amplitude reduction factor S_0_^2^ value (0.870) was applied in the EXAFS analysis to determine the coordination numbers (CNs) for the scattering path in sample.

**3.2 Data analysis for Ge**

For EXAFS modeling, the global parameters (CN, R, σ^2^ and ΔE_0_) were obtained by nonlinear fitting and least-squares refinement of the EXAFS equation applied to the Fourier-transformed data in R-space, using Artemis software. EXAFS of the Ge foil was fitted and the obtained amplitude reduction factor S_0_^2^ value (1.0) was used in the EXAFS analysis to determine the CNs for the scattering path in sample.

**4. Computational details**

All calculations were carried out with CP2K package (version 7.1) in the framework of the density functional theory (DFT), based on the hybrid Gaussian and plan-wave scheme. Molecular orbitals of the valence electrons were expanded using the DZVP-MOLOPT-SR-GTH basis sets,^6^ and the exchange-correlation between the electrons was treated with the Predew-Burke-Ernzerhof (PBE) functional^8^, supplemented with the Grimmes D3 dispersion correction. The interaction between the valence electrons and atomic cores was described by the norm-conserving Goedecker-Teter-Hutter (GTH) pseudopotentials^8^. A plane-wave density cutoff of 500 Ry was adopted. All the structures were fully relaxed by CP2K with BFGS scheme, and the force convergence criterion was set to 4.5$\times$10^-4^ hartree/bhor.

**5. Complementary characterization**

**
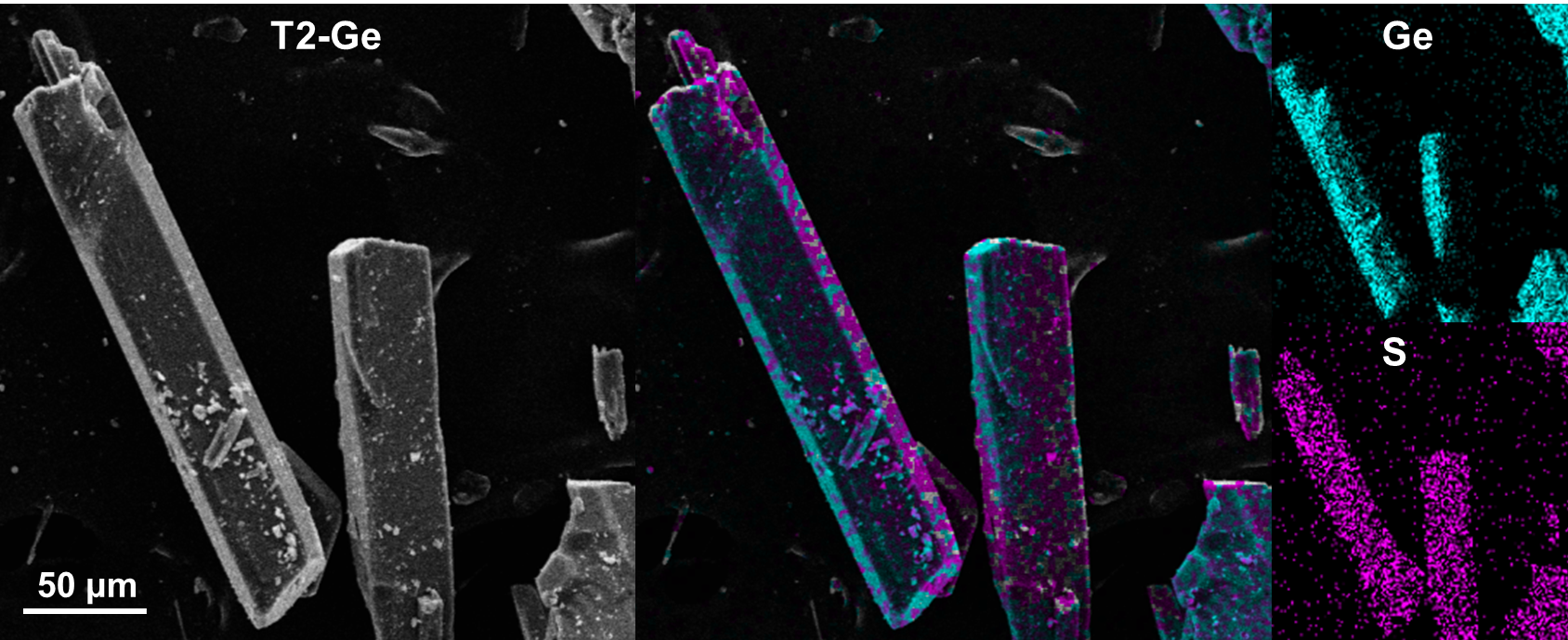
**

**Figure S1.** SEM image and EDS mapping of T2-Ge crystals.


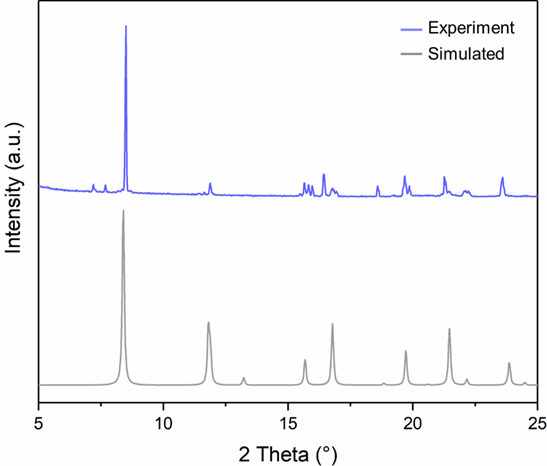


**Figure S2.** PXRD patterns of T2-Ge.

**
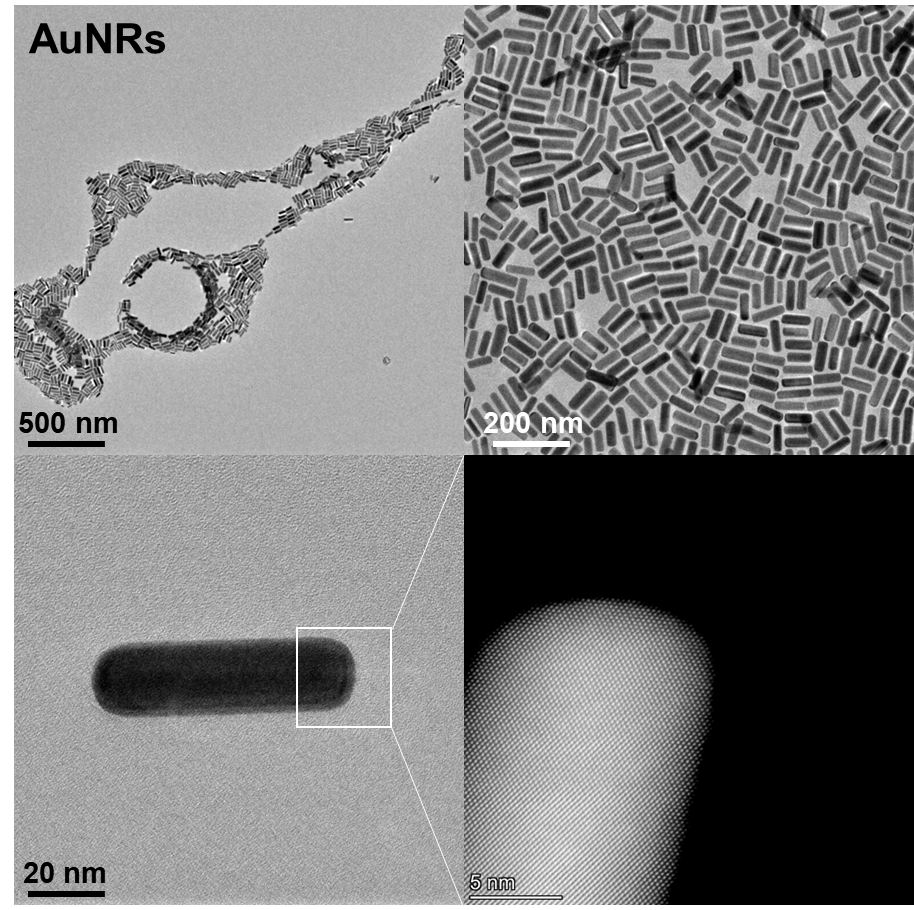
**

**Figure S3.** HAADF-STEM image of **AuNRs**.

**
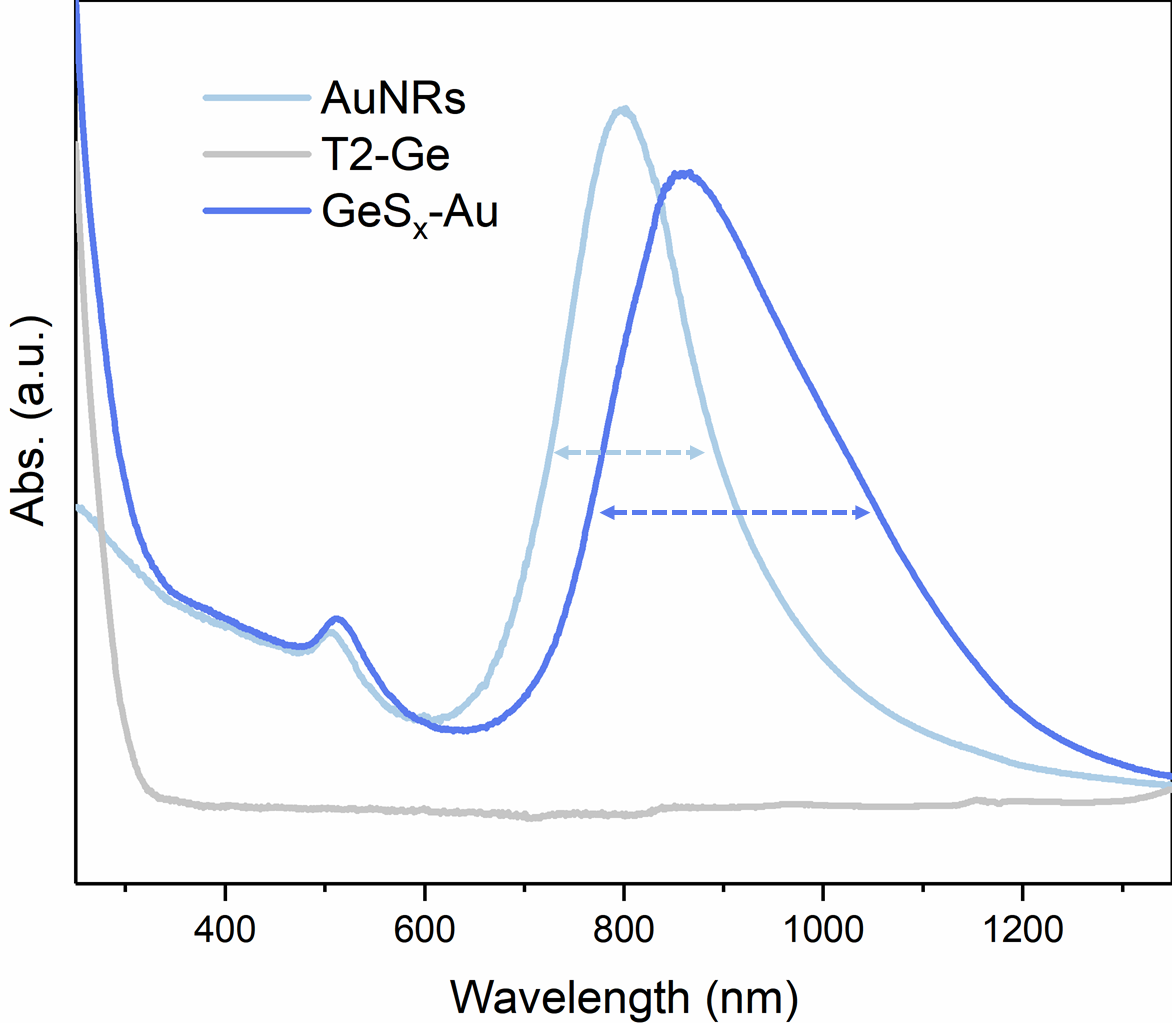
**

**Figure S4.** Comparison of UV-vis-NIR absorption spectra of **AuNRs**, T2-Ge and **GeS_x_-Au**.

**
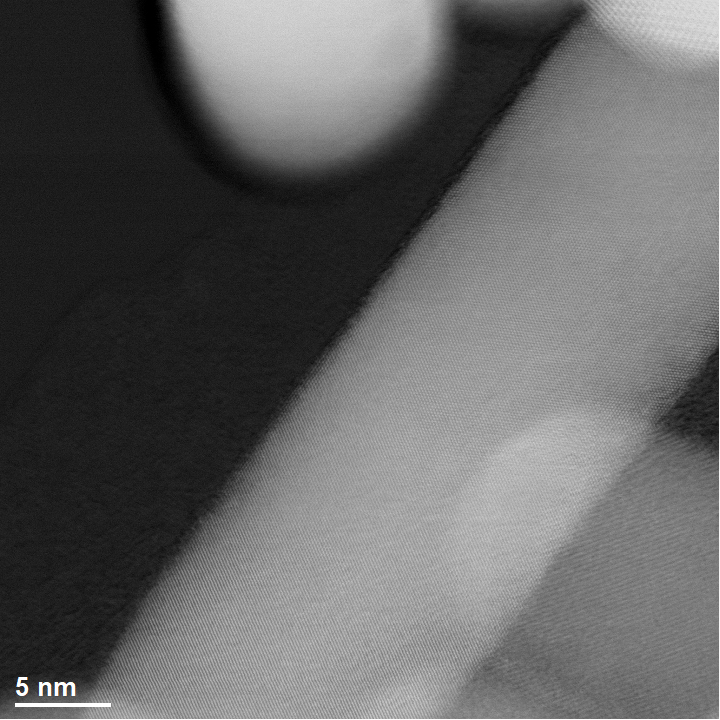
**

**Figure S5.** Cs-corrected HAADF-STEM image of **GeS_x_-Au** (T2 concentration of 0.45 mg/mL).

**
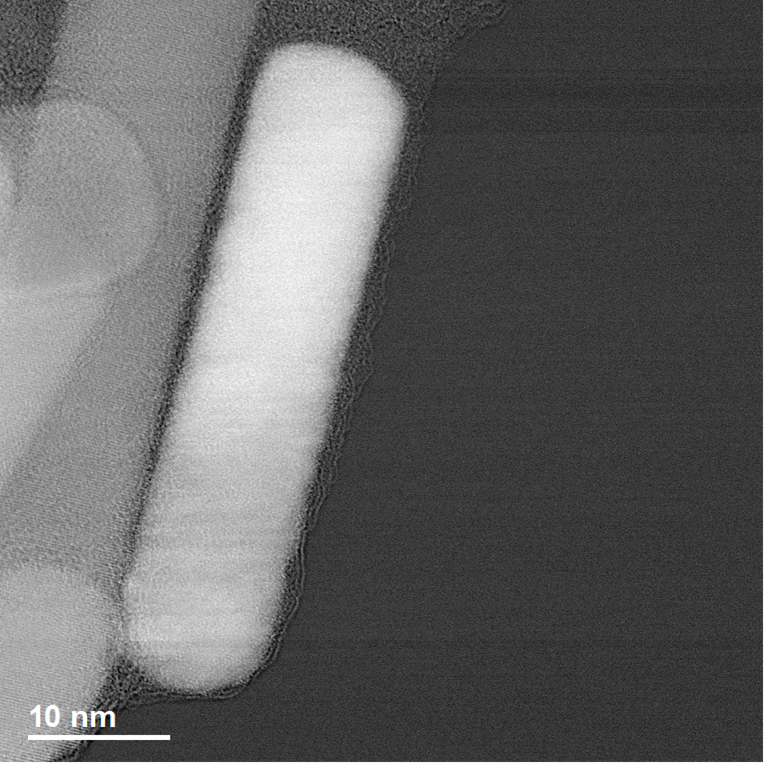
**

**Figure S6.** Cs-corrected iDPC-STEM image of **GeS_x_-Au** (T2 concentration of 0.65 mg/mL).

**
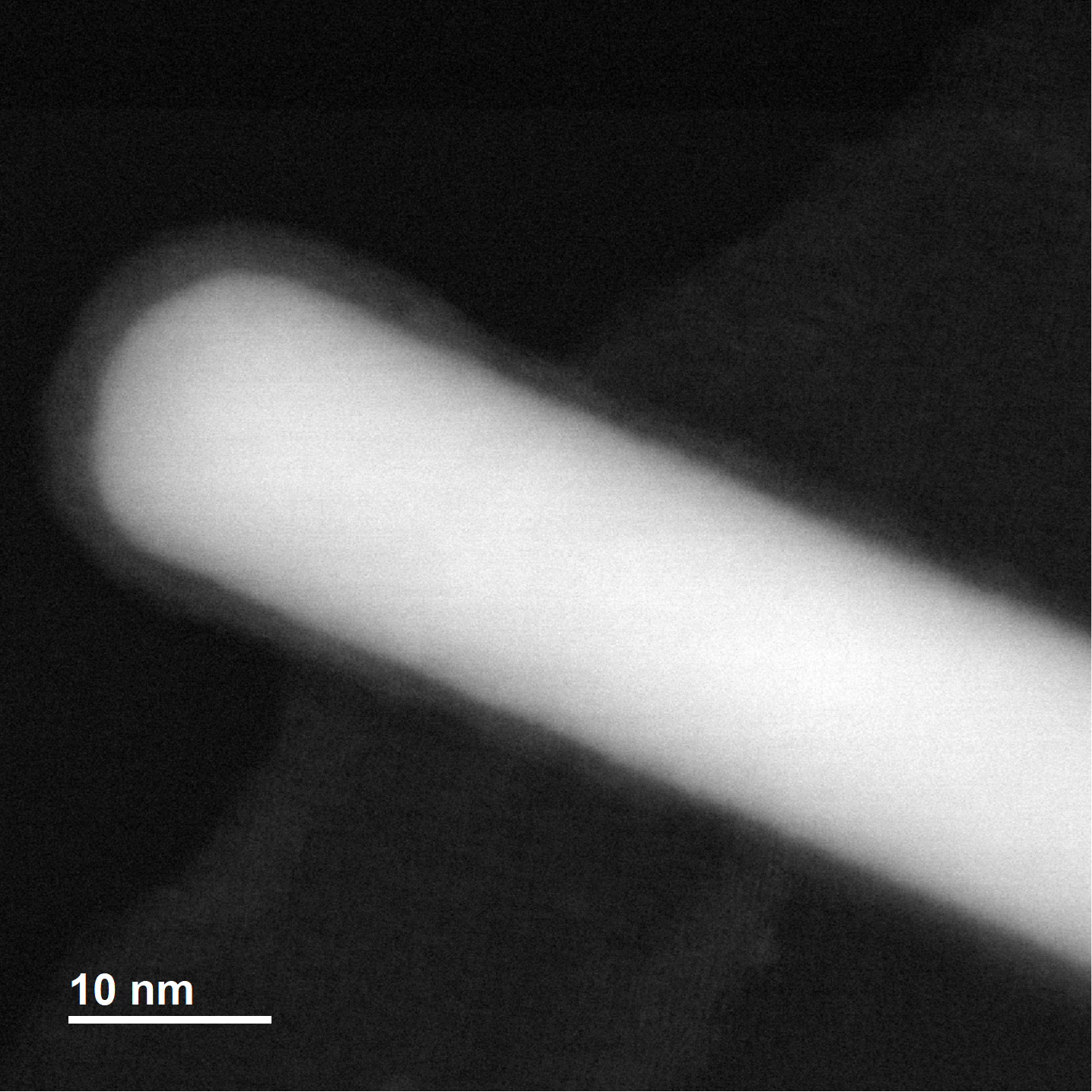
**

**Figure S7.** This is a Cs-corrected HAADF-STEM image of **GeS_x_-Au** (T2 concentration of 0.75 mg/mL).

**
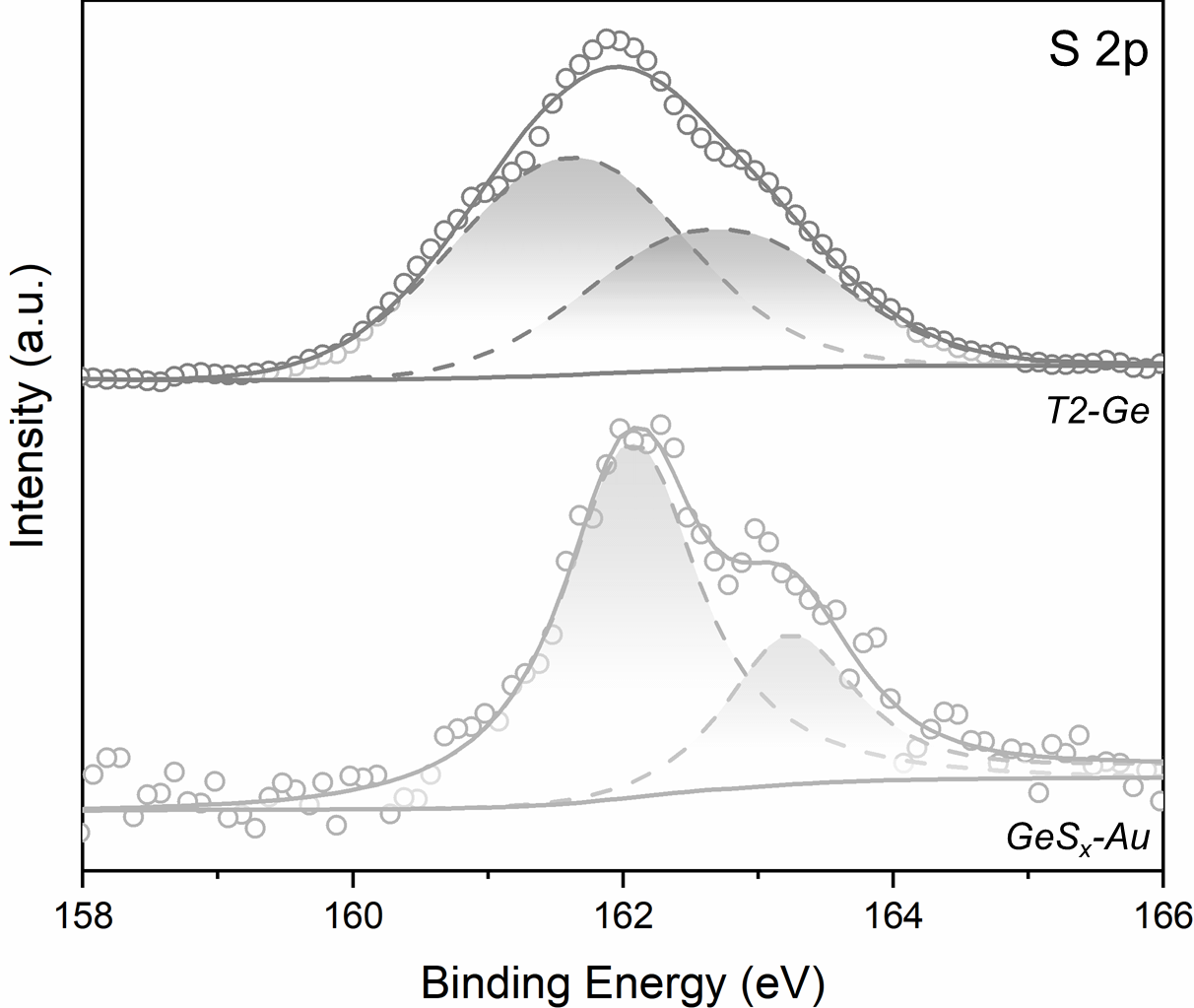
**

**Figure S8.** S 2p XPS spectra before and after compositing.


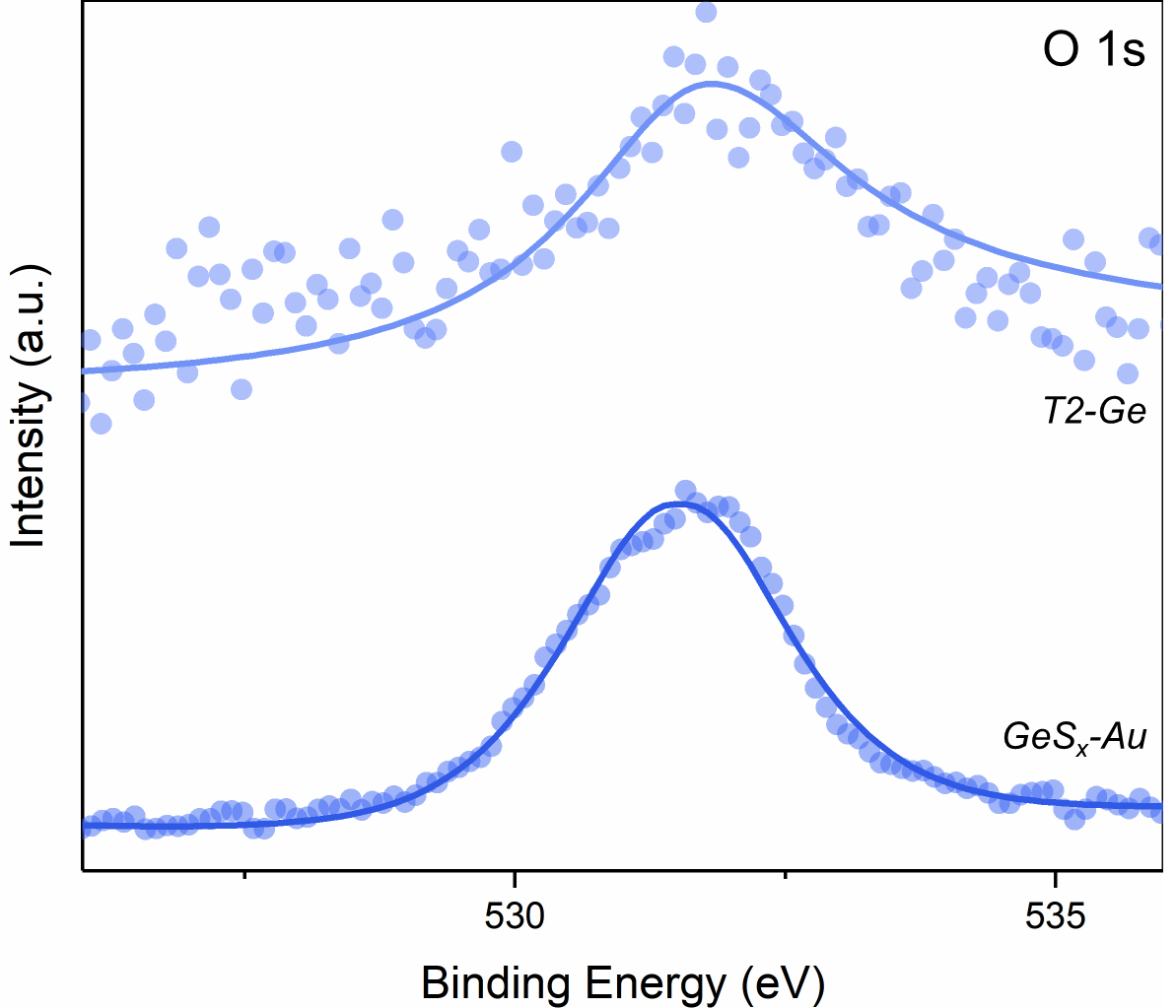


**Figure S9.** O 1s XPS spectra before and after compositing.


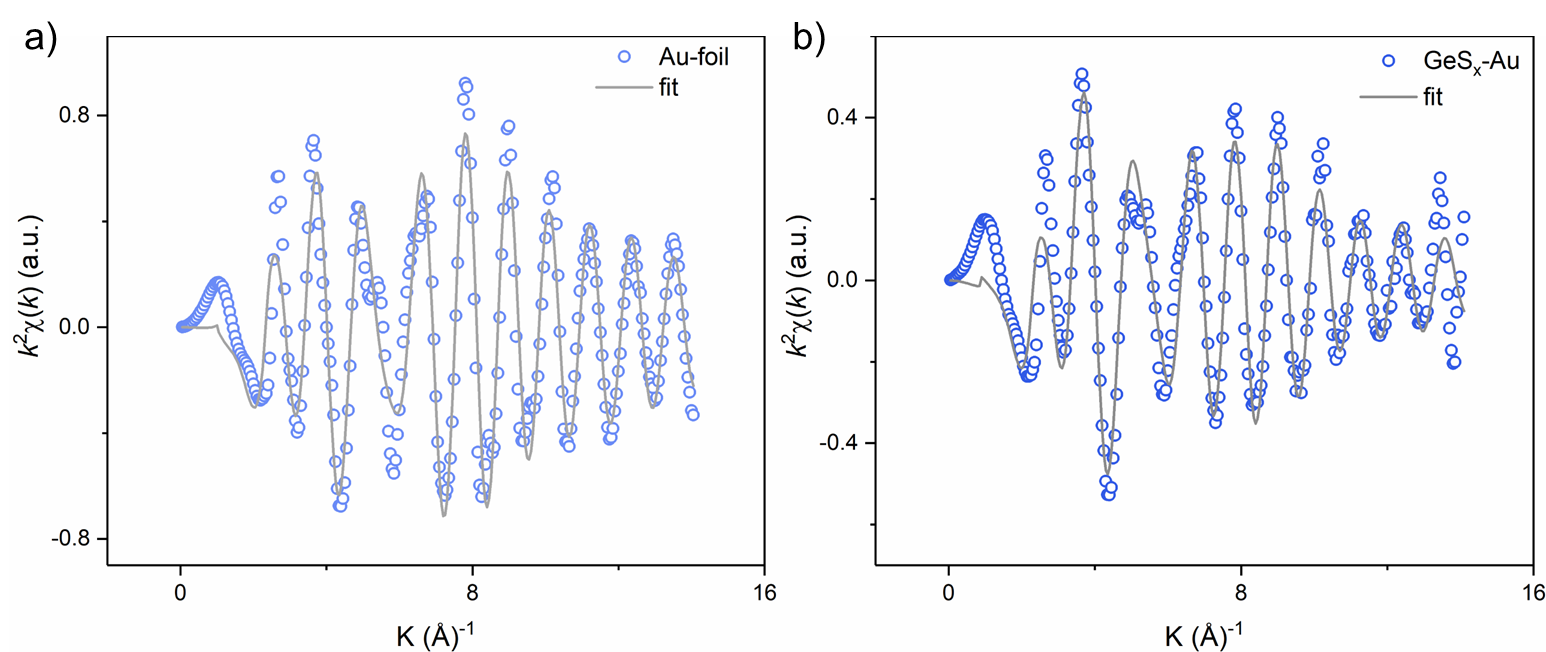


**Figure S10.** *K*-space fitting of (a) Au-foil and (b) **GeS_x_-Au**.


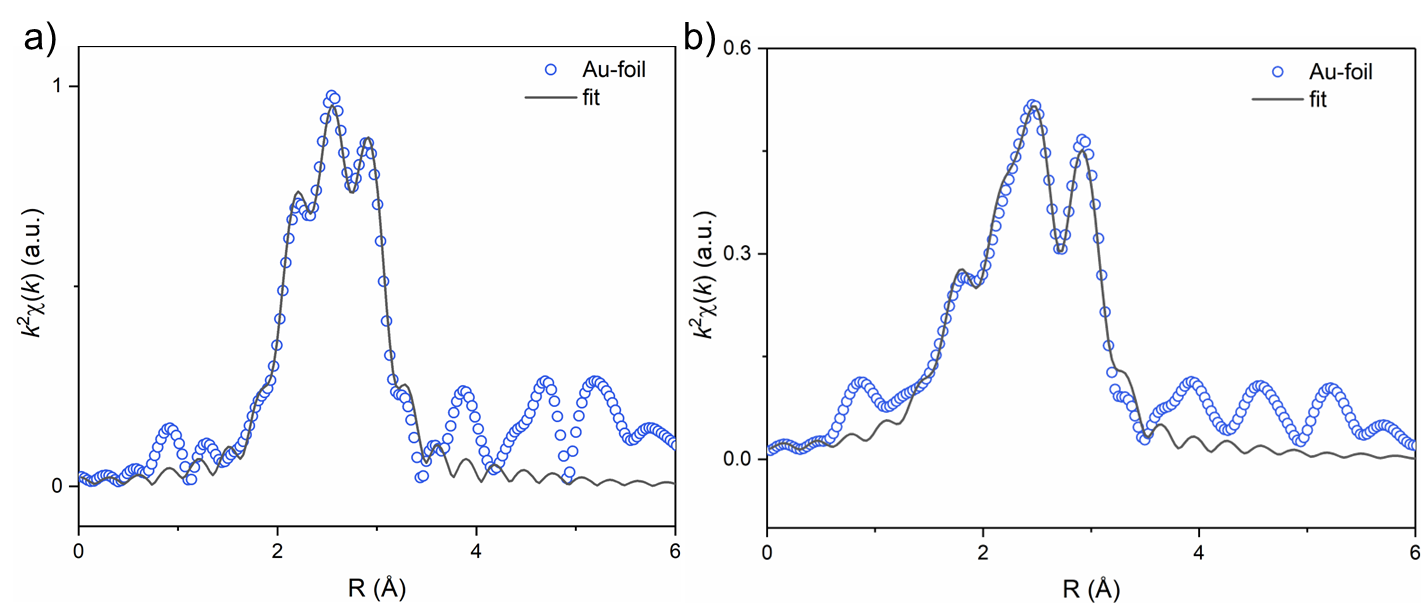


**Figure** **S11.** *R*-space fitting of (a) Au-foil and (b) **GeS_x_-Au**.


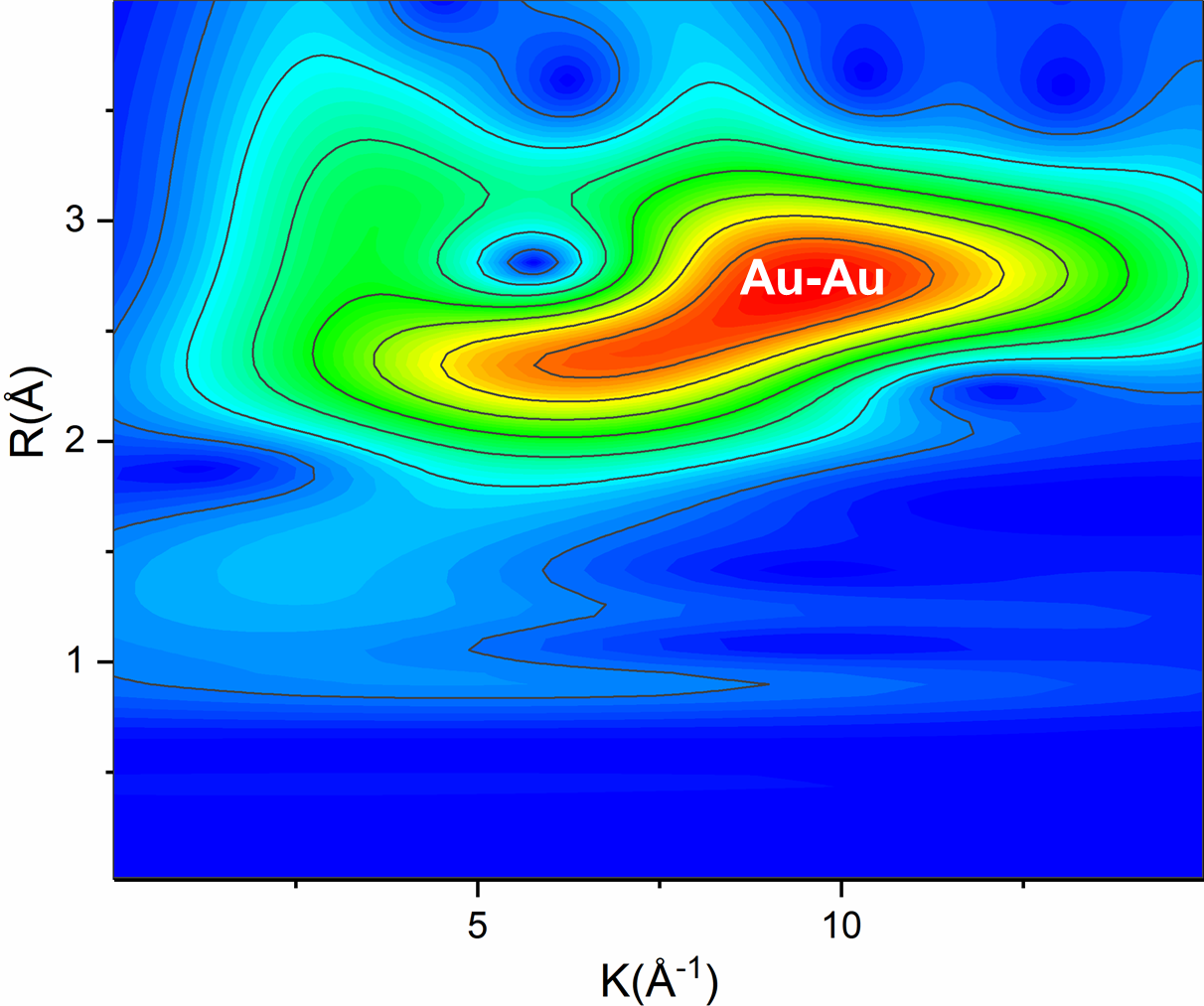


**Figure S12.** Wavelet transform analysis of the Au L3 edge for Au-foil.


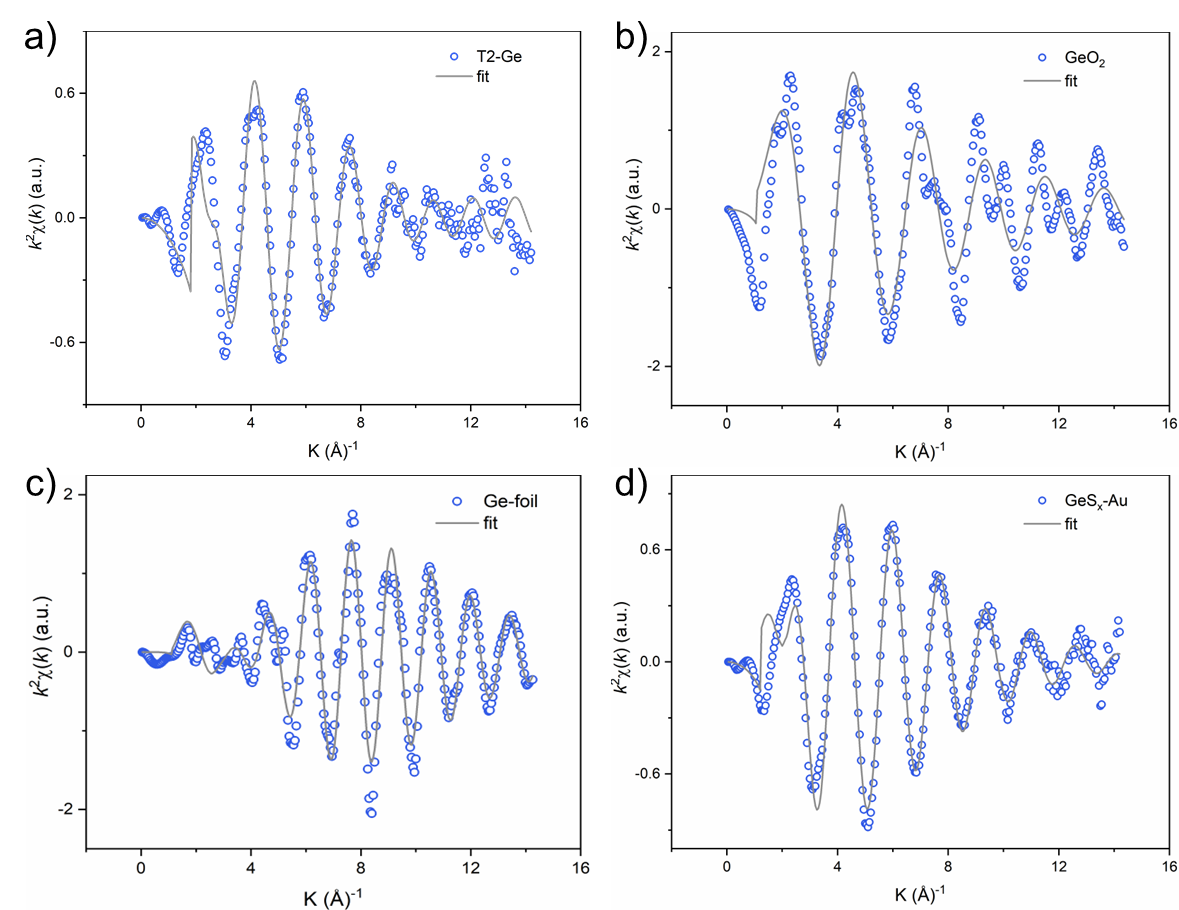


**Figure S13.** *K*-space fitting of (a) T2-Ge, (b) GeO_2_, (c) Ge-foil, and (d) **GeS_x_-Au**.


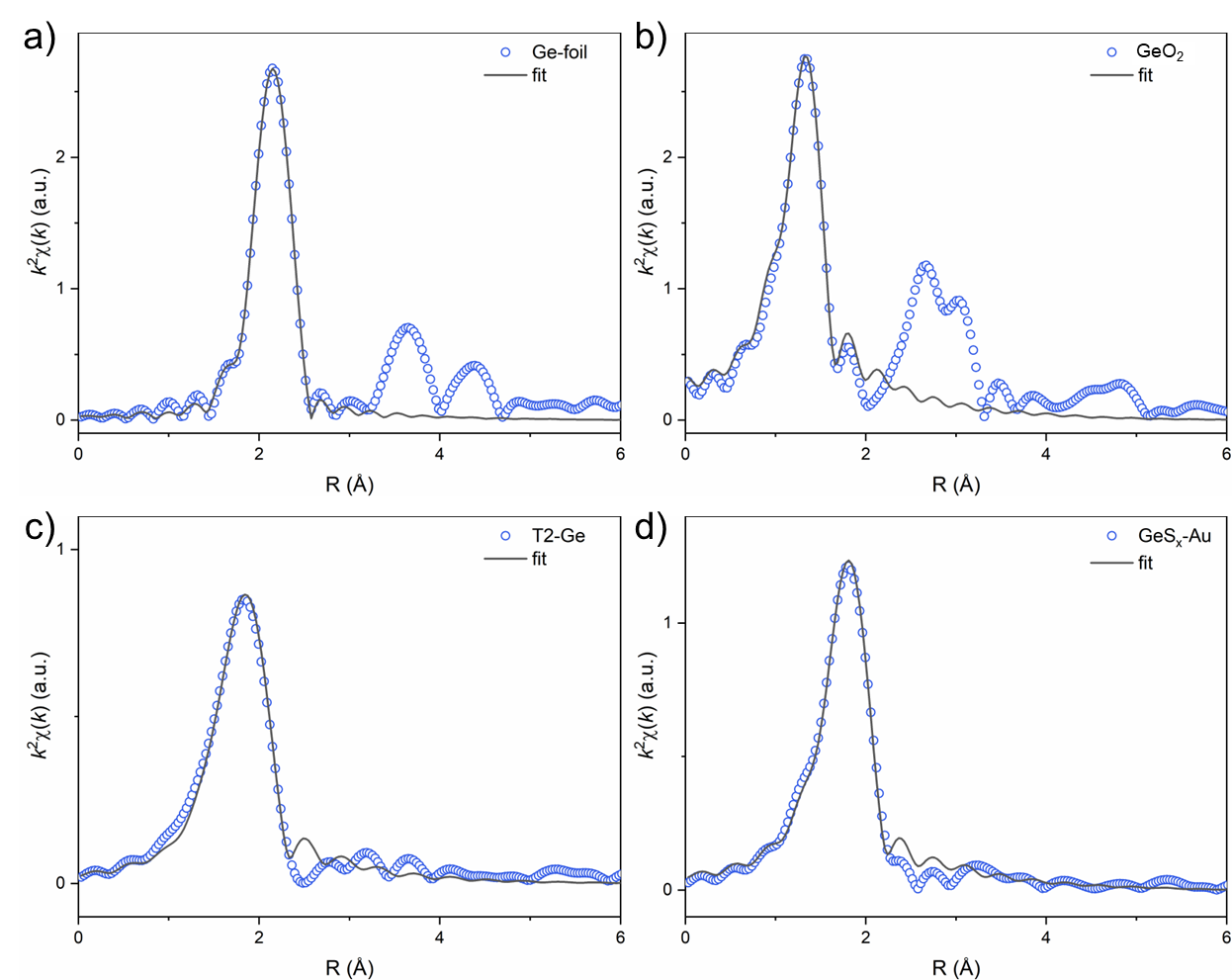


**Figure S14.** *R*-space fitting of (a) Ge-foil, (b) GeO_2_, (c) T2-Ge and (d) **GeS_x_-Au**.


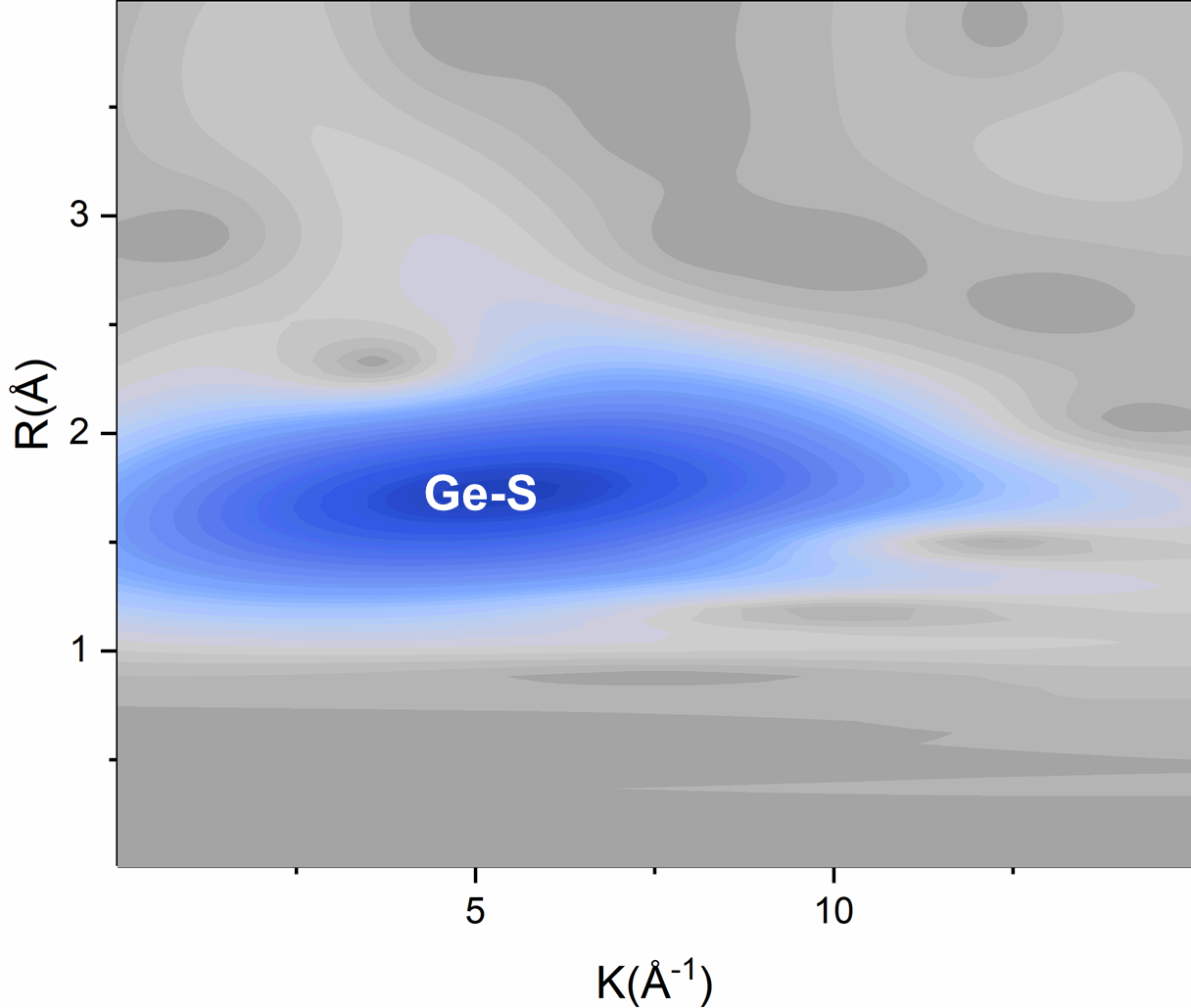


**Figure S15.** Wavelet transform analysis of Ge K edge for T2-Ge.


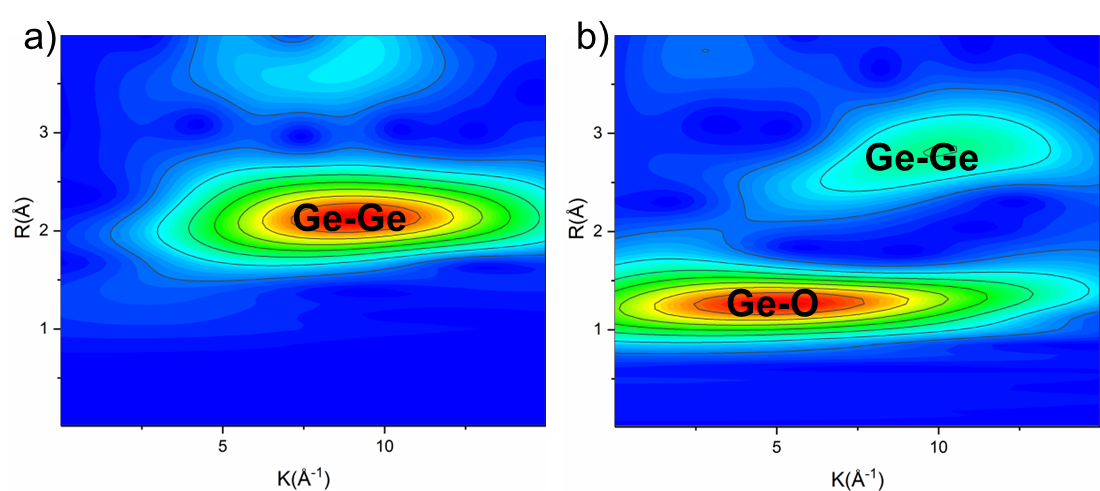


**Figure S16.** Wavelet transform of Ge K edge for (a) Ge-foil and (b) GeO_2_.

**
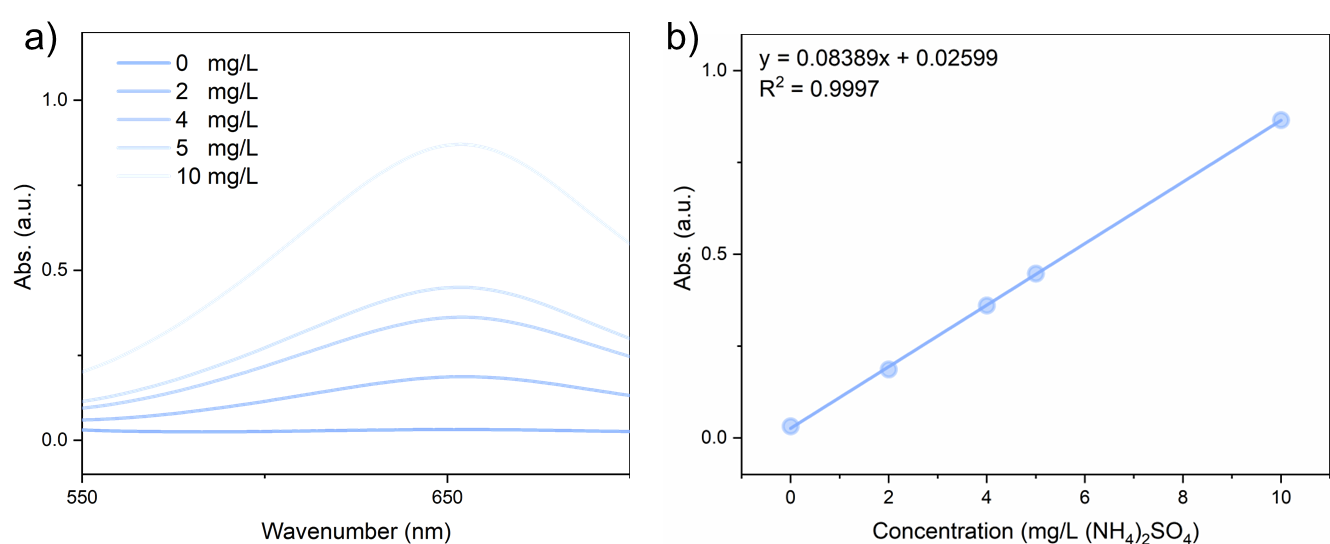
**

**Figure** **S17.** (a) UV-vis absorption spectra of 0, 2, 4, 5, and 10 mg/L ammonium sulfate solutions using the indophenol blue colorimetric method and (b) corresponding standard curve based on absorbance and (NH_4_)_2_SO_4_ concentration.

**
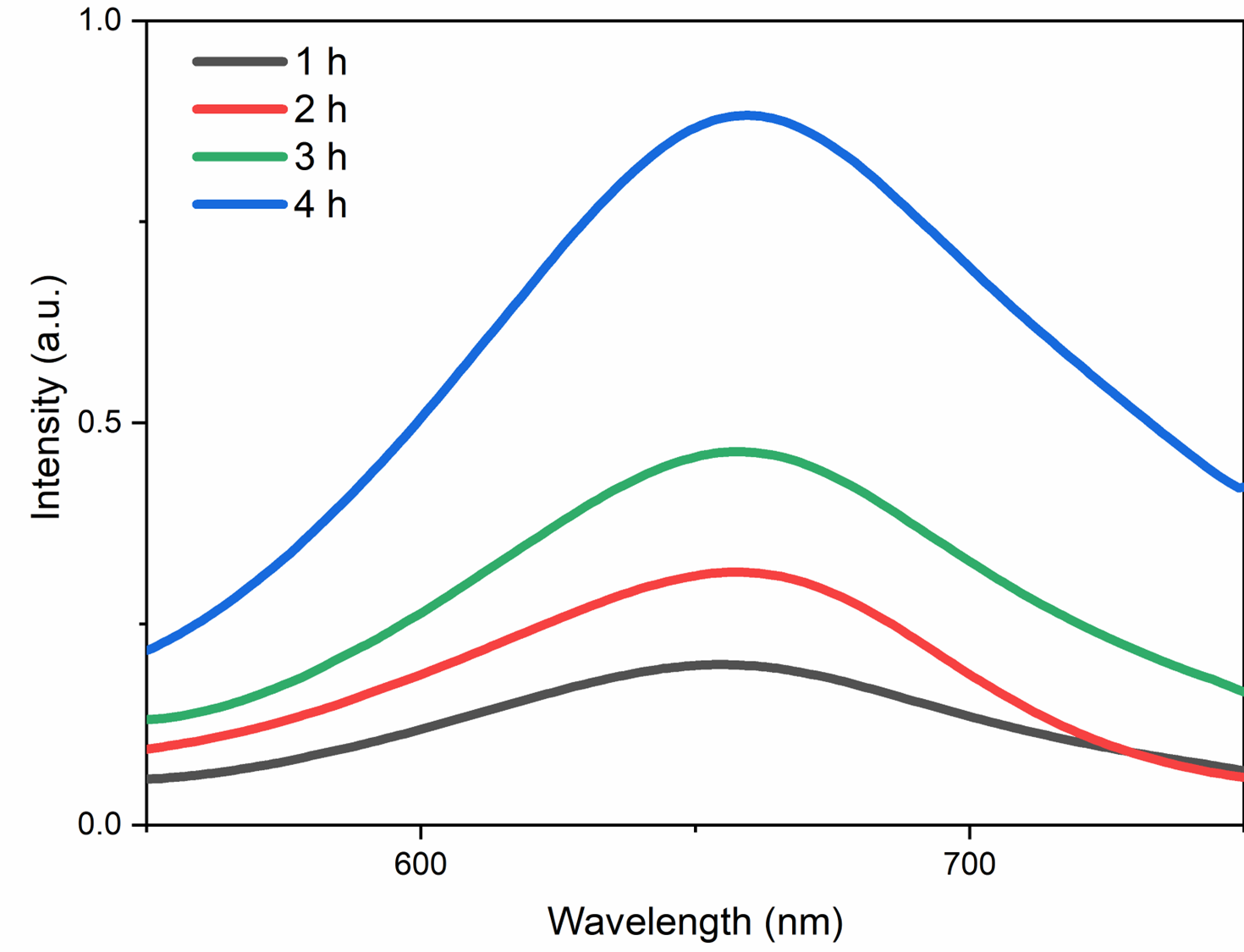
**

**Figure S18.** Detection of NH_4_^+^ concentration in the **GeS_x_-Au** system after photocatalytic nitrogen fixation reactions for 1, 2, 3, and 4 h using indophenol blue colorimetric assay.


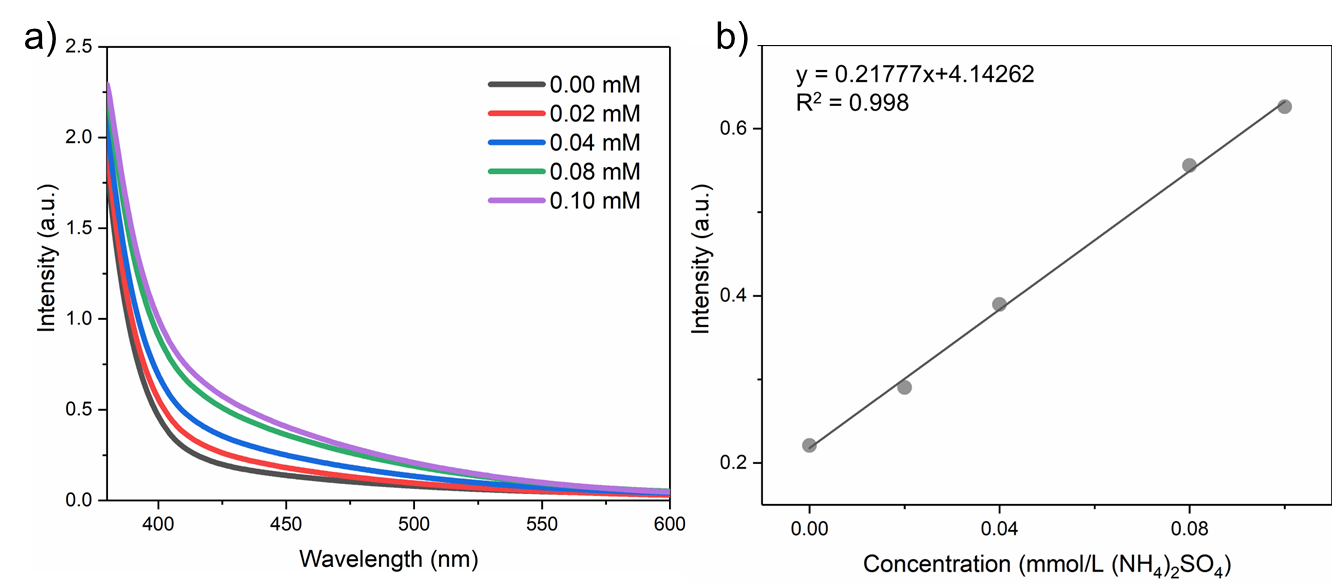


**Figure S19.** (a) UV-vis absorption spectra of 0, 2, 4, 5, and 10 mg/mL ammonium sulfate solutions using the Nessler reagent method and (b) corresponding standard curve based on absorbance and (NH_4_)_2_SO_4_ concentration.


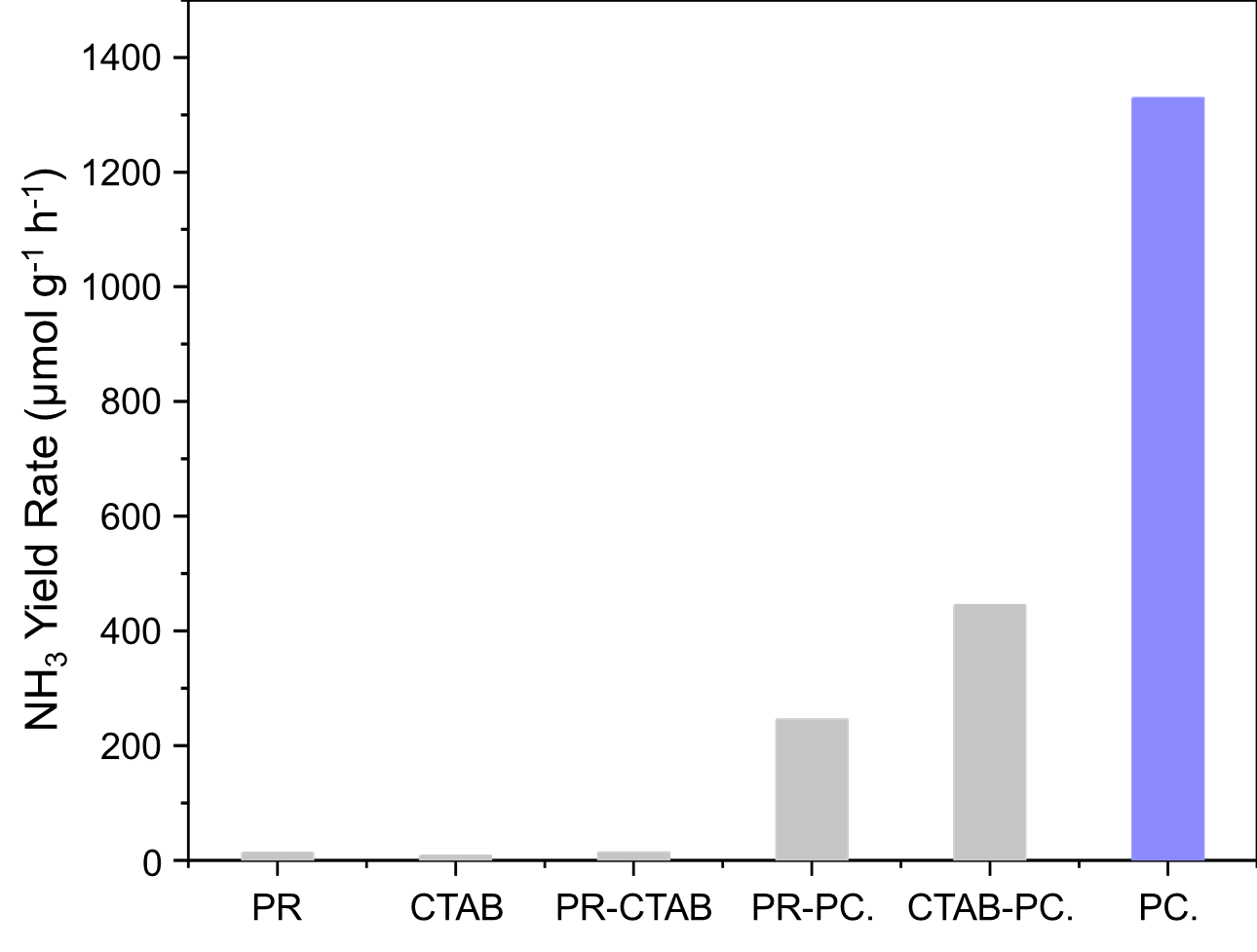

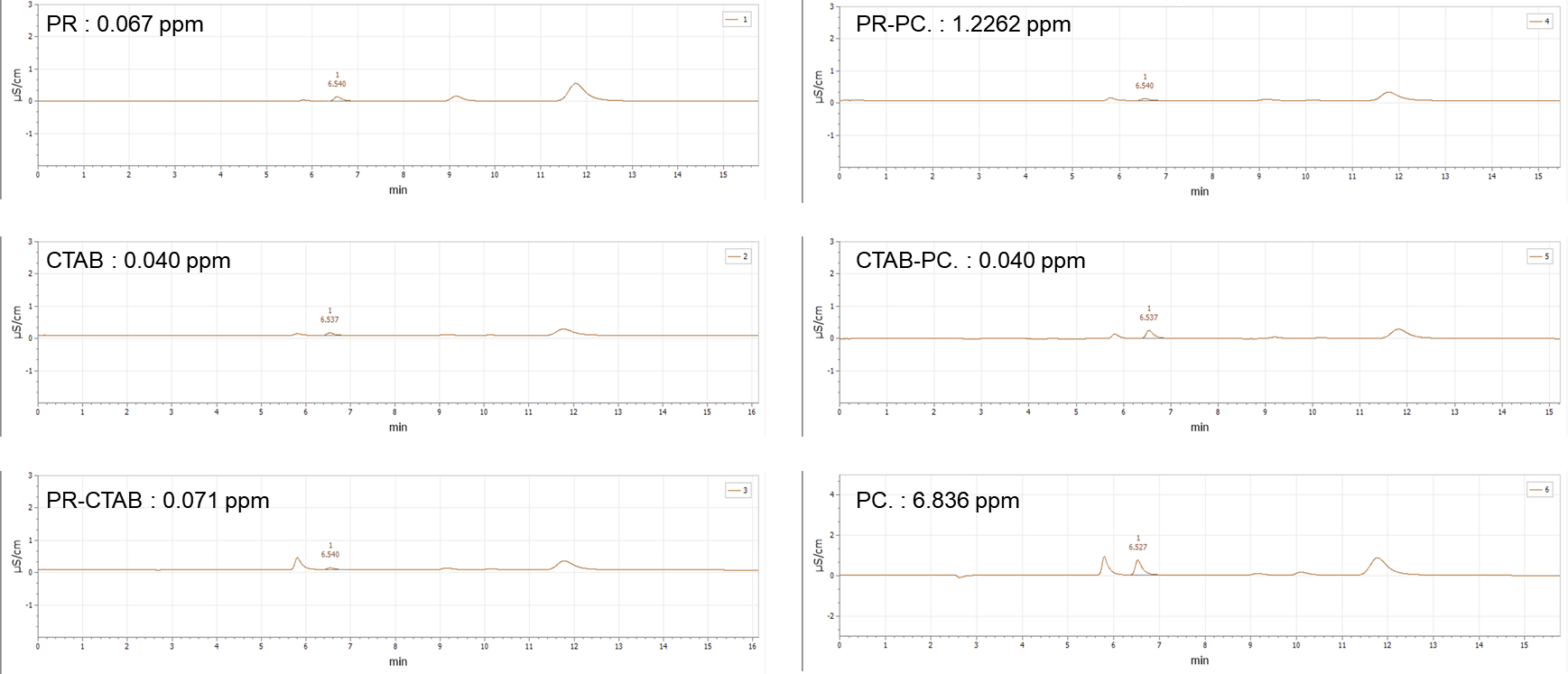


**Figure S20.** Ion chromatography test results for different additives.


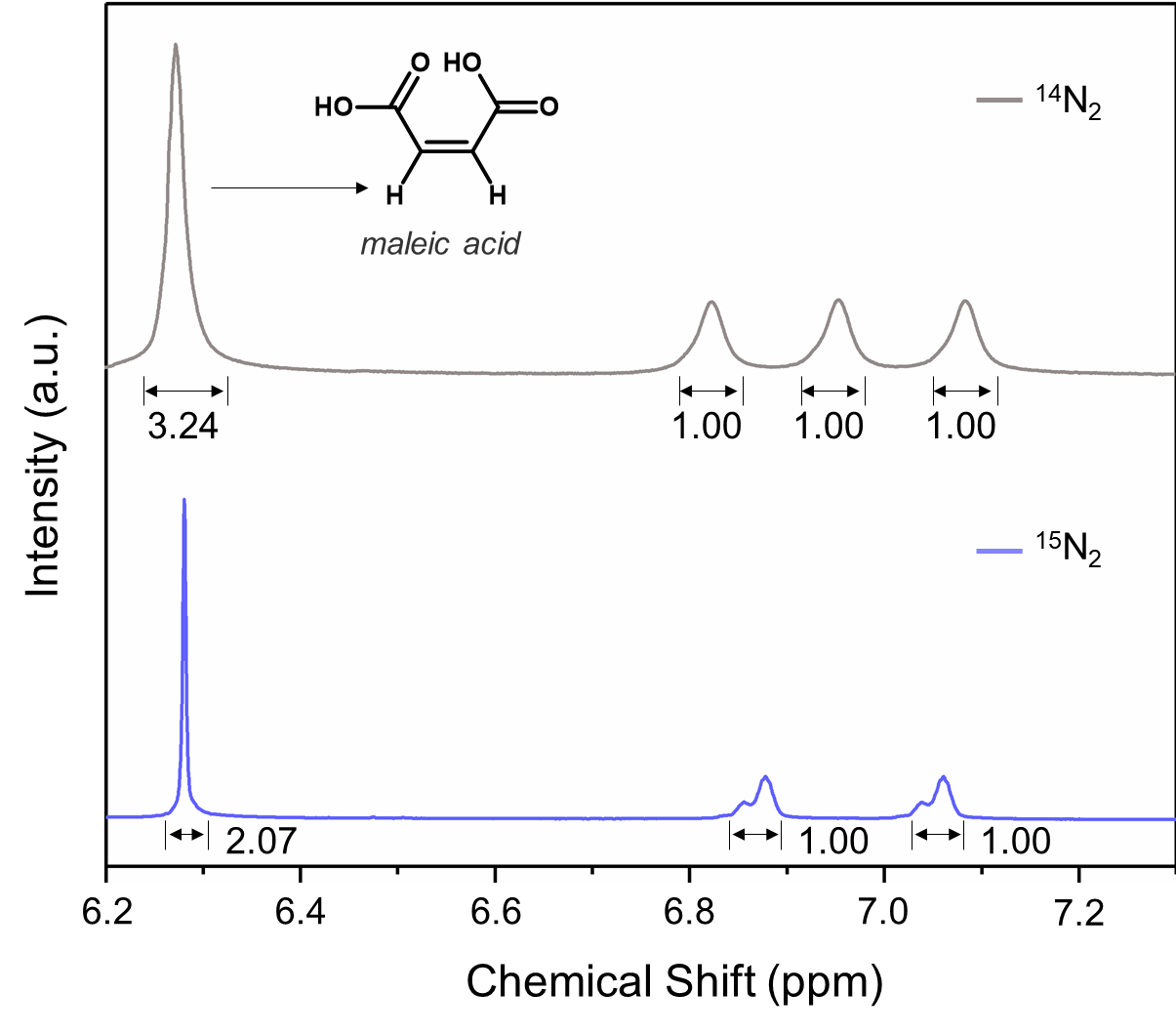


**Figure S21.** Using maleic acid as an internal standard, ^14^N_2_ and ^15^N_2_ were used as substrates for ^1^H NMR quantitative calibration.


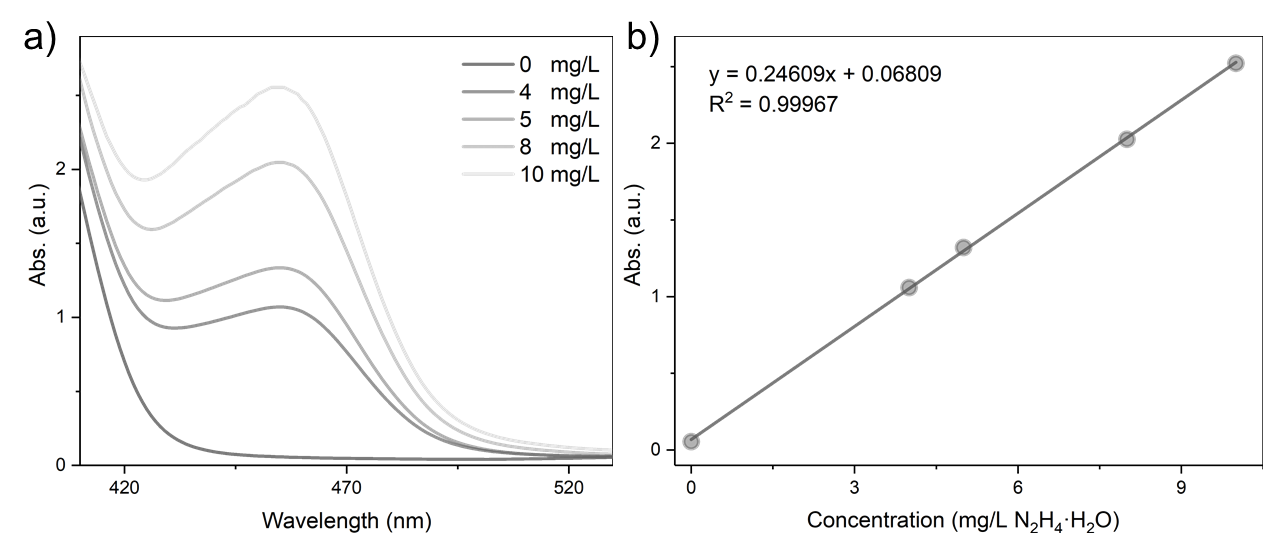


**Figure S22.** (a) UV-vis absorption spectra of 0, 2, 4, 5, and 10 mg/mL ammonium sulfate solutions using ultraviolet colorimetry and (b) corresponding standard curve based on absorbance and N_2_H_4_ concentration.


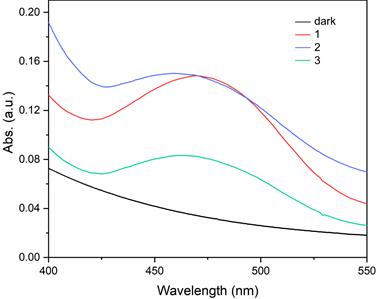


**Figure S23.** Colorimetric test results obtained from parallel triple tests.


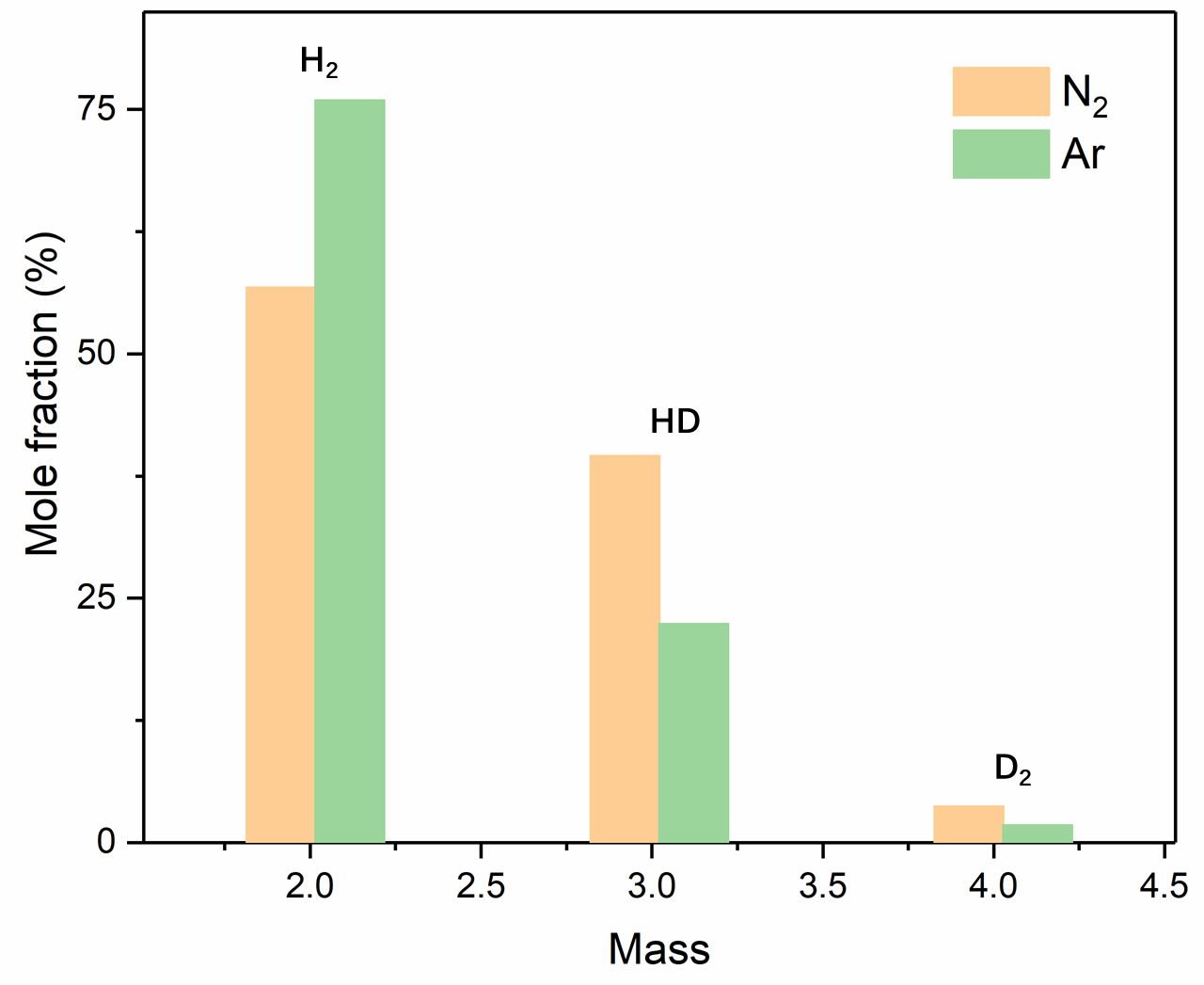


**Figure S24**. H-D gas isotope analysis results under N_2_ and Ar atmosphere.


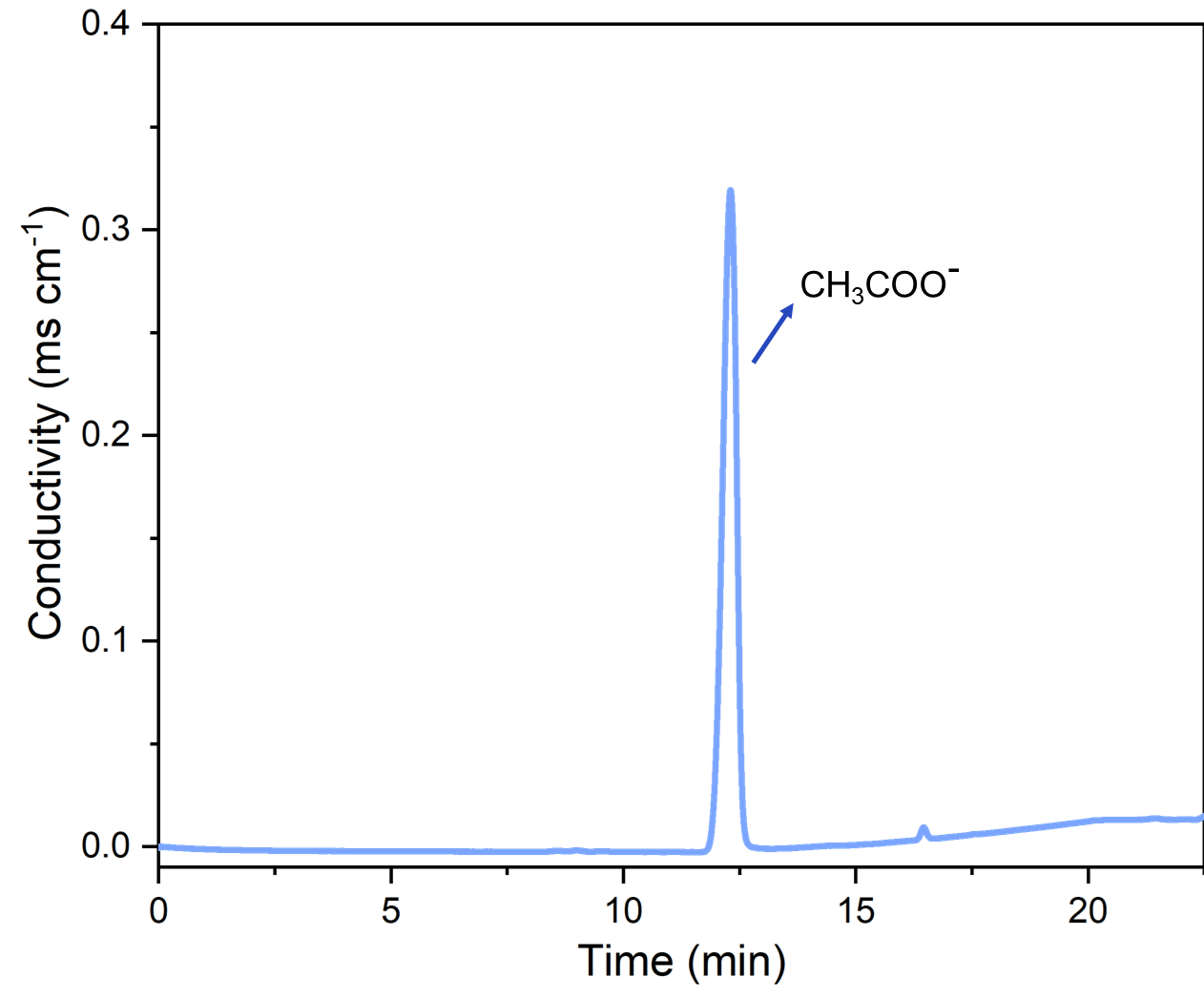


**Figure S25.** Ion chromatographic detection of liquid-phase products after catalysis by **GeS_x_-Au**.


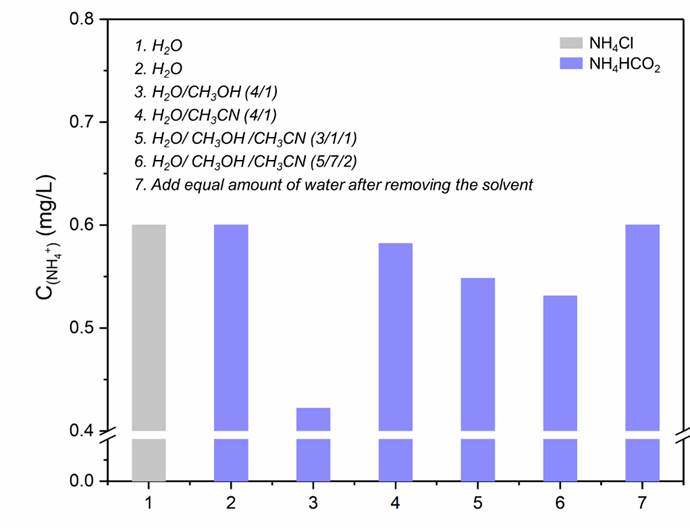


**Figure S26.** Results of indigo carmine colorimetric detection in different solvents.


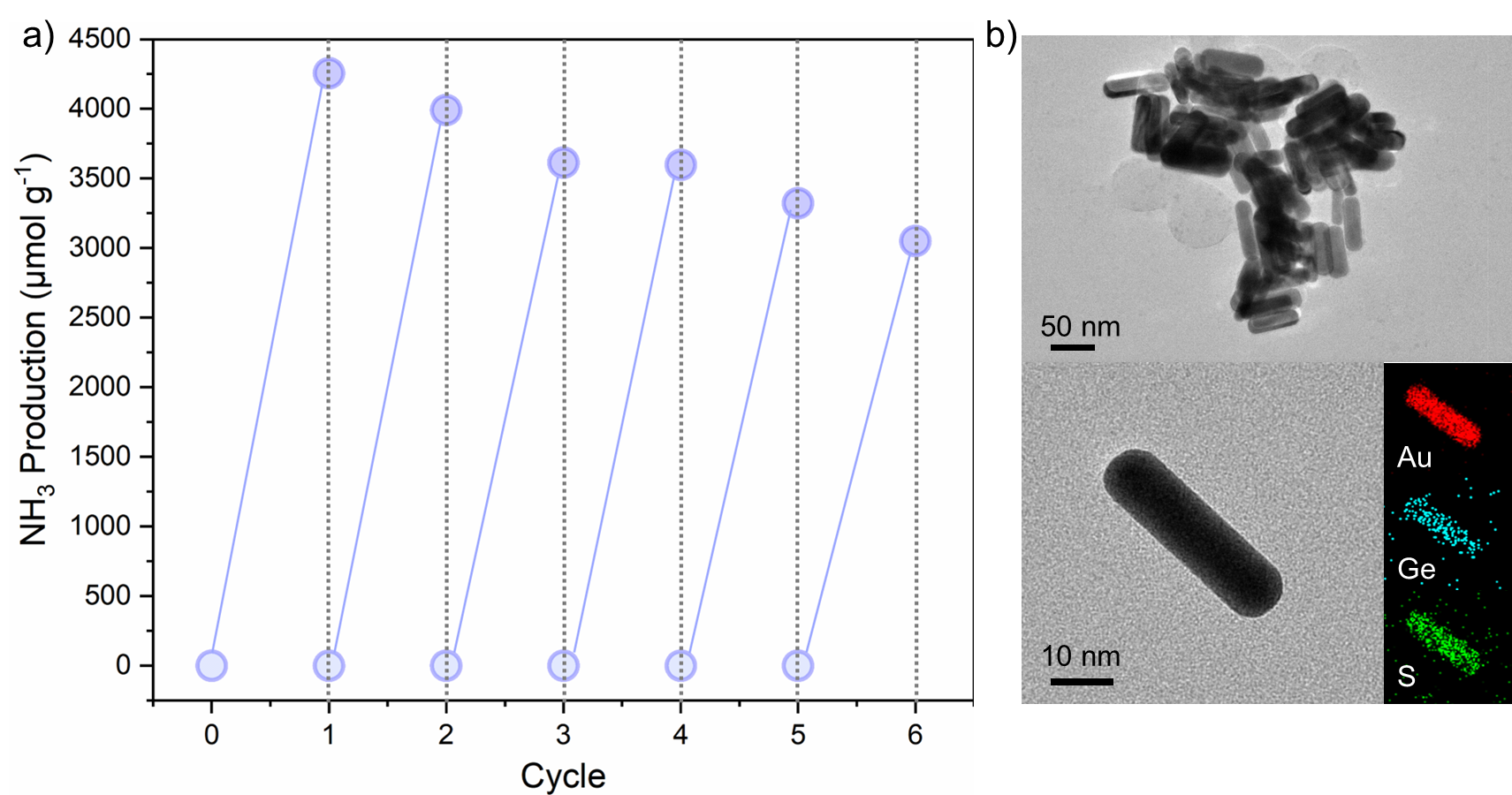


**Figure S27.** (a) Recyclability of the photocatalytic nitrogen fixation reaction by **GeS_x_-Au** over three cycles; (b) After 6 cycles, TEM-mapping results of **GeS_x_-Au**.


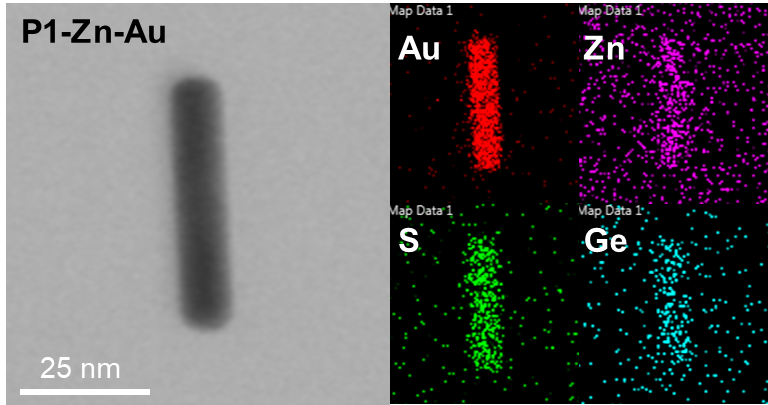


**Figure S28.** TEM image and EDS mapping results of P1-Zn-Au.


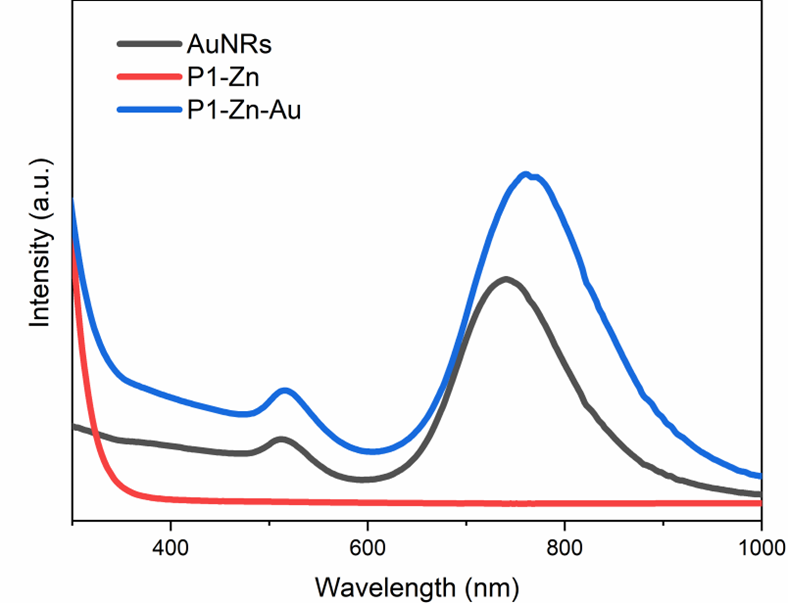


**Figure S29.** UV-vis-NIR absorption spectra of P1-Zn, **AuNRs**, and P1-Zn-Au.


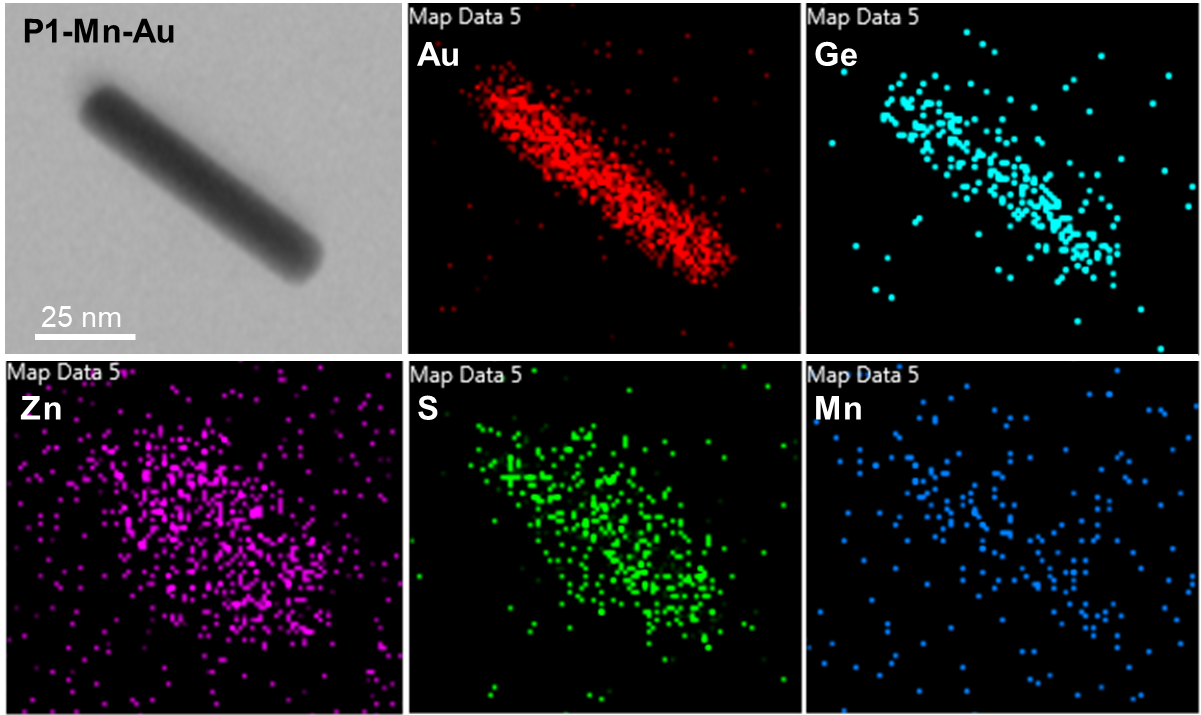


**Figure S30.** TEM image and mapping results of P1-Mn-Au.


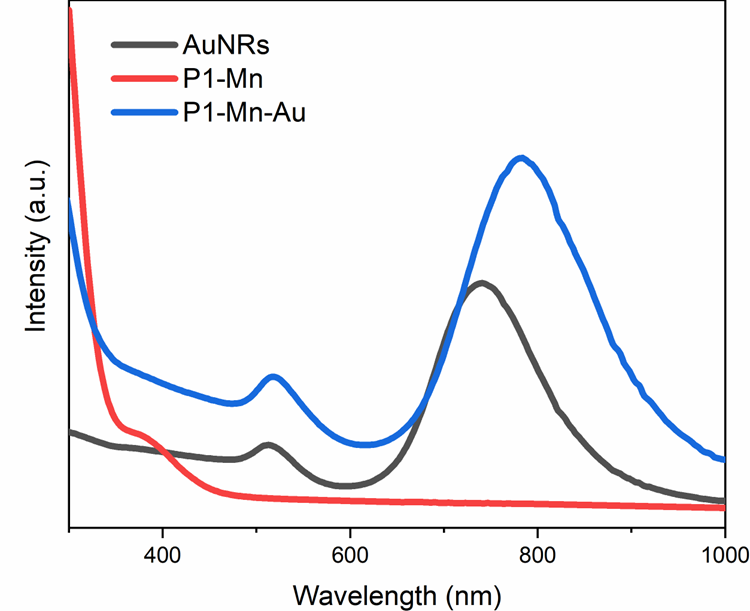


**Figure S31.** UV-vis-NIR absorption spectra of P1-Mn, **AuNRs**, and P1-Mn-Au.


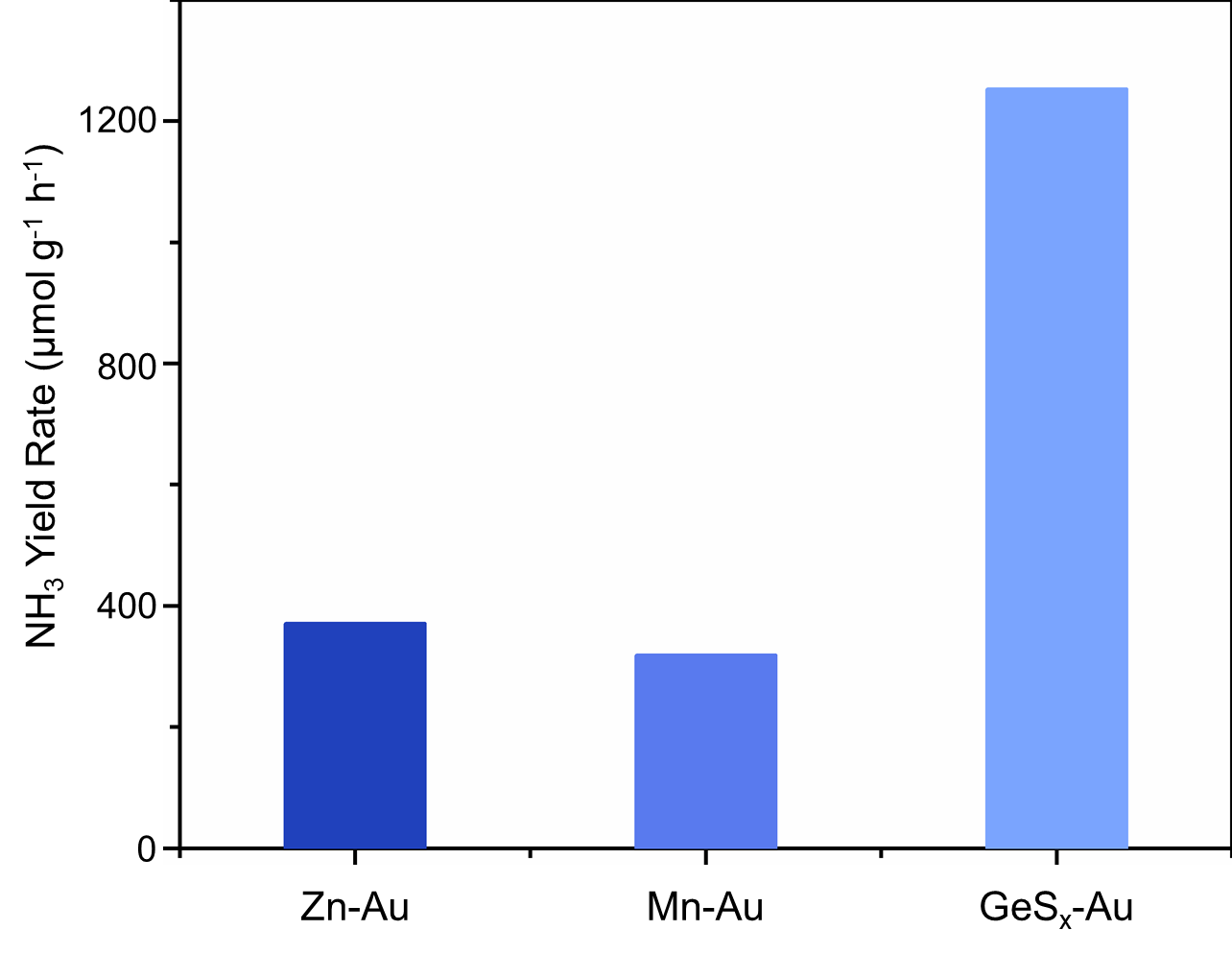


**Figure S32.** Comparison of NH_3_ production rates among various materials.

**
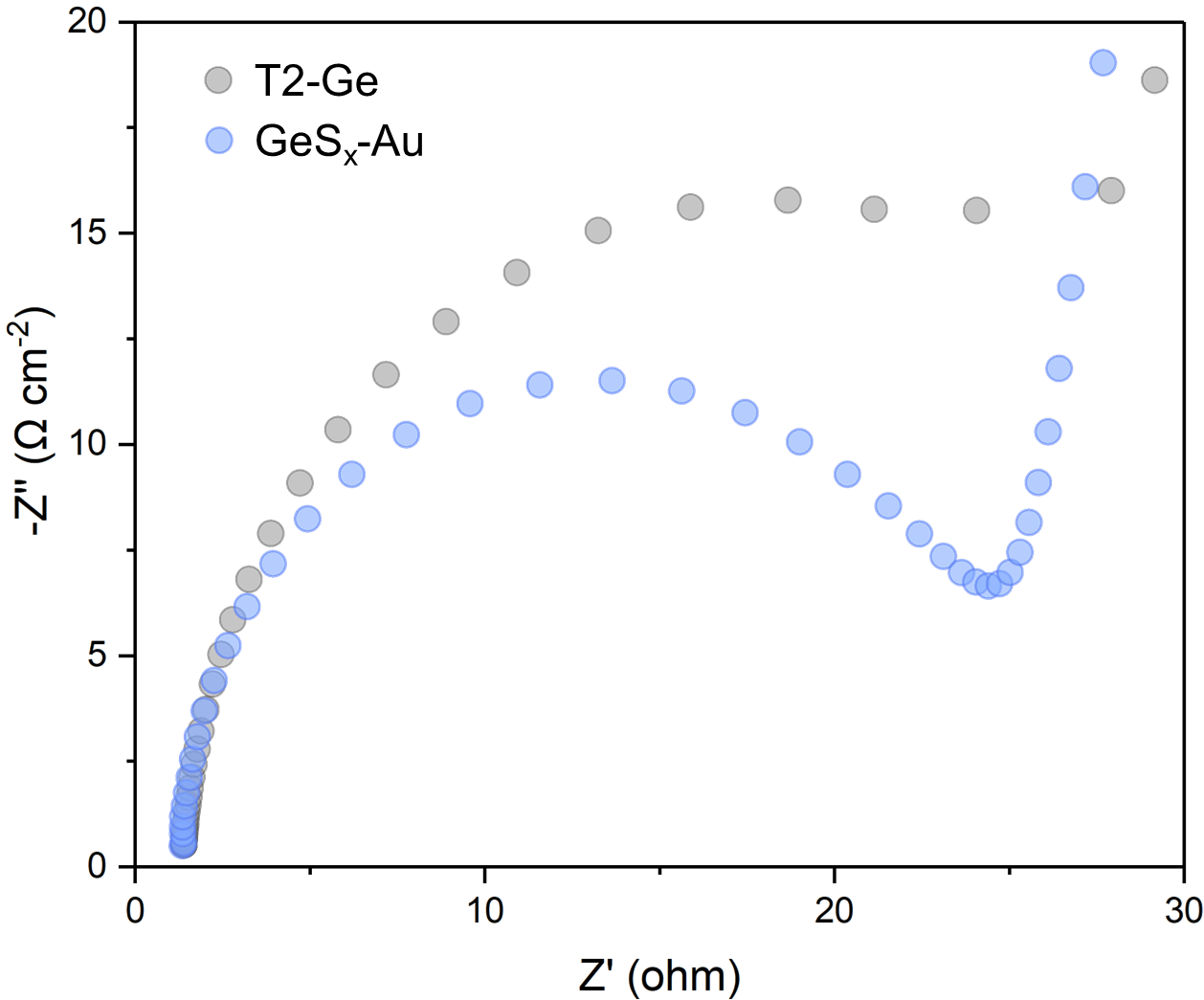
**

**Figure S33.** Electrochemical impedance diagram of **GeS_x_-Au** and **T2-Ge**.

*
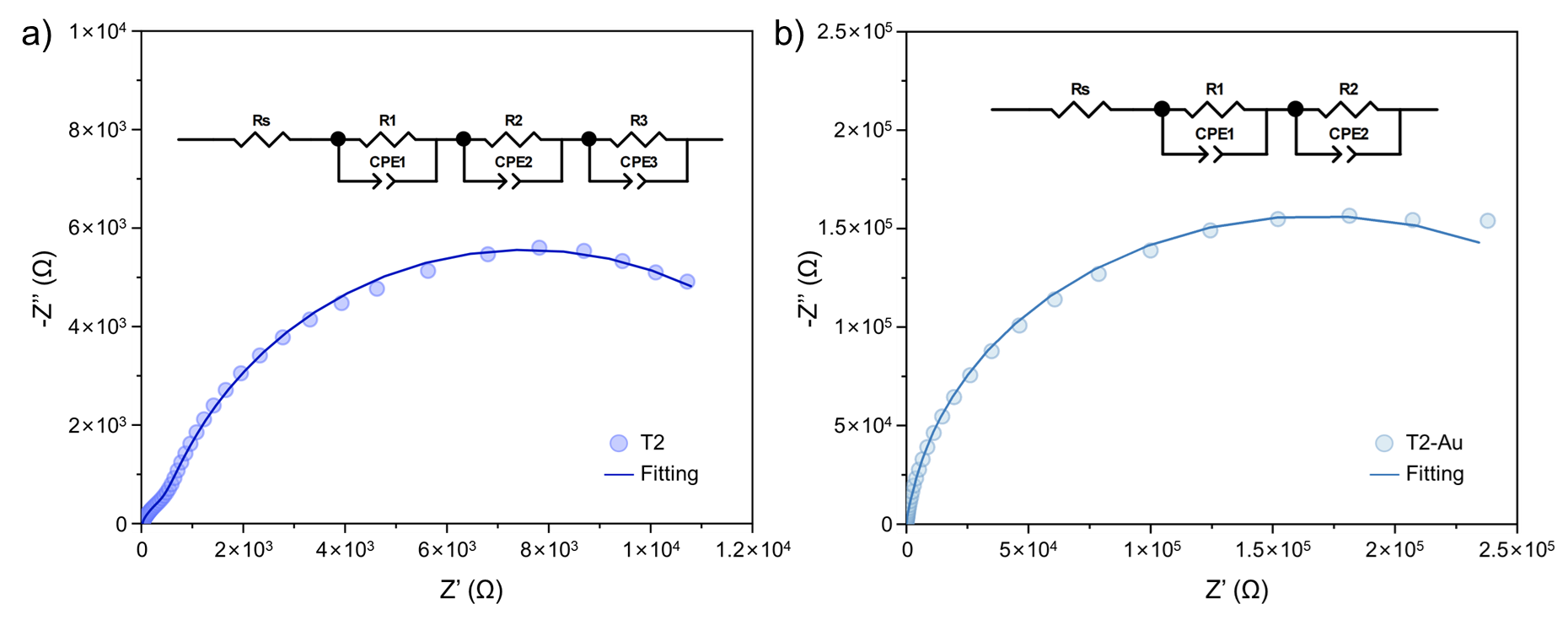
*

**Figure S34.** Equivalent Circuit Fitting of (a) T2 and (b) **GeS_x_-Au**.

**
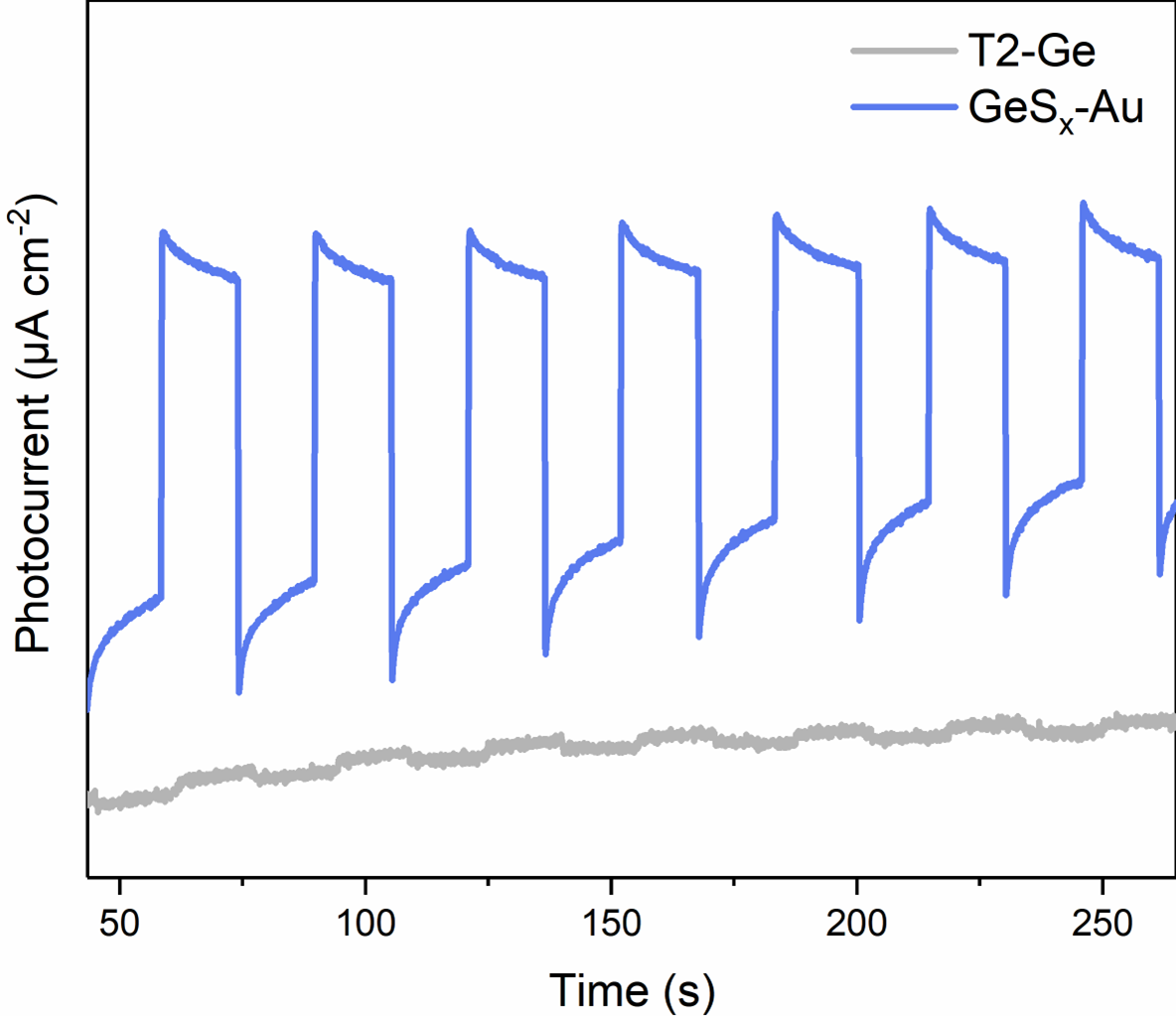
**

**Figure S35**. Photocurrent response performance for **GeS_x_-Au** and **T2-Ge**.


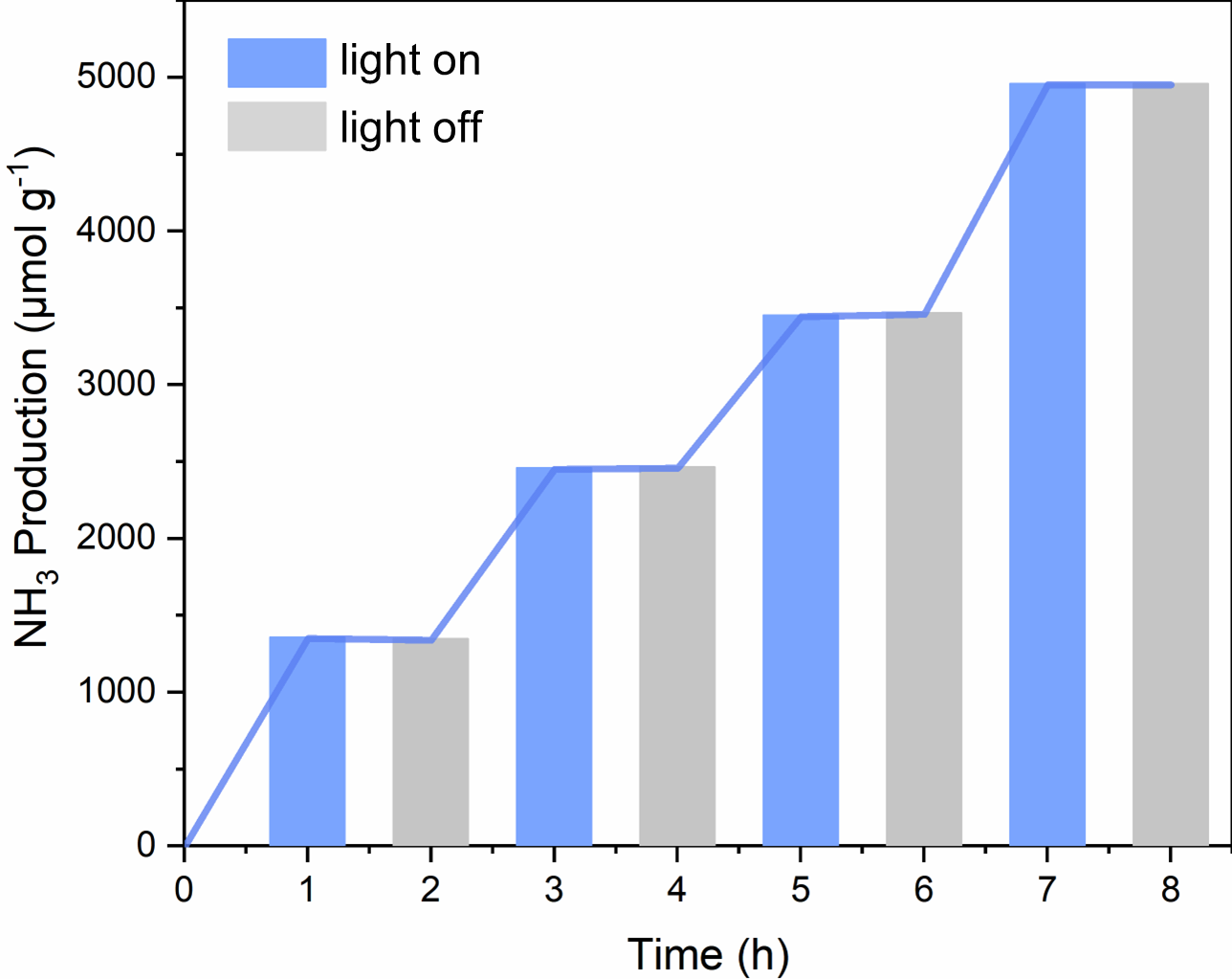


**Figure S36.** Catalytic nitrogen fixation experiments using **GeS_x_-Au** in an on-off light cycle.


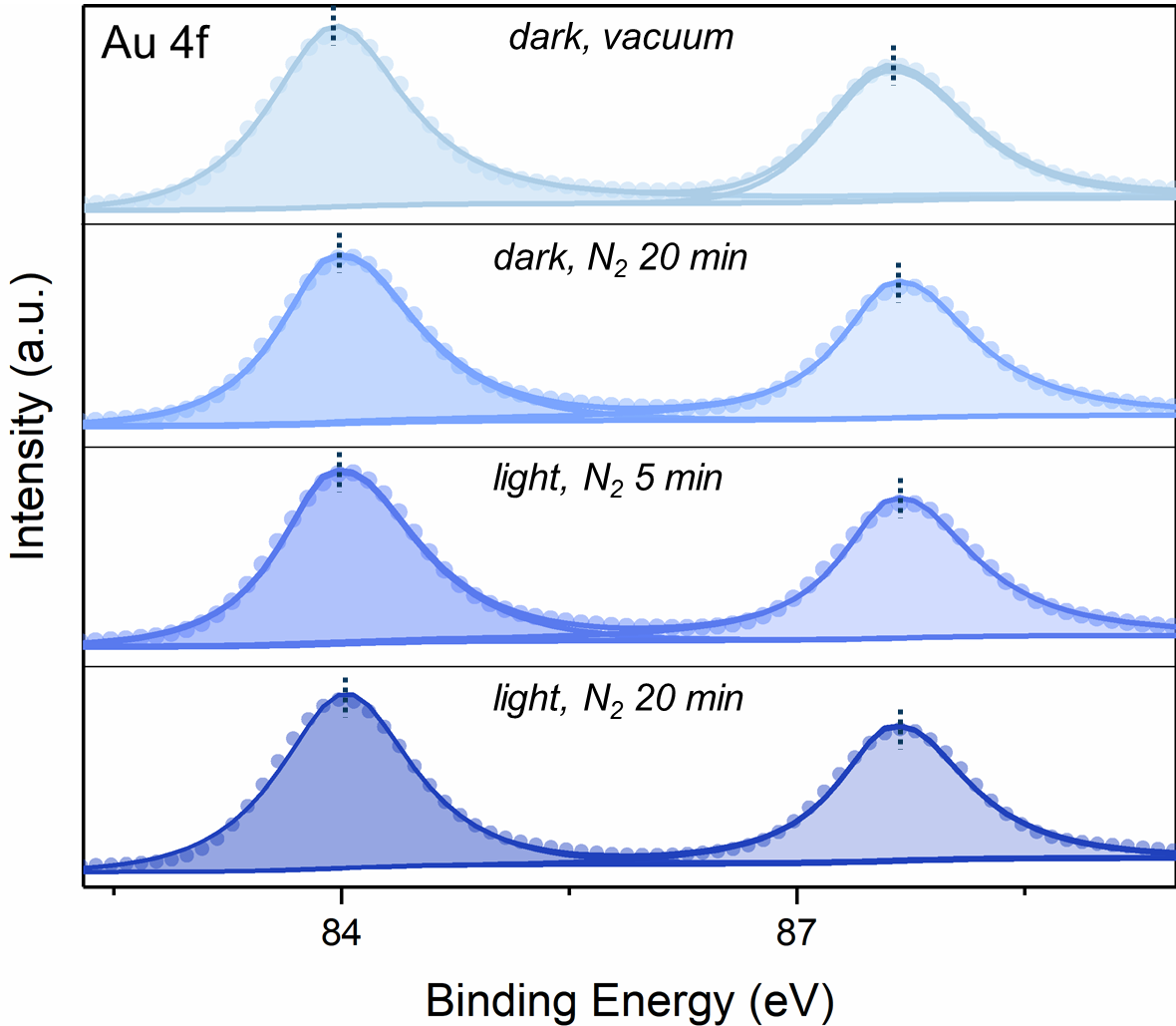


**Figure S37.** In situ XPS changes of Au 4f in **GeS_x_-Au** under N_2_ atmosphere, dark and light conditions.


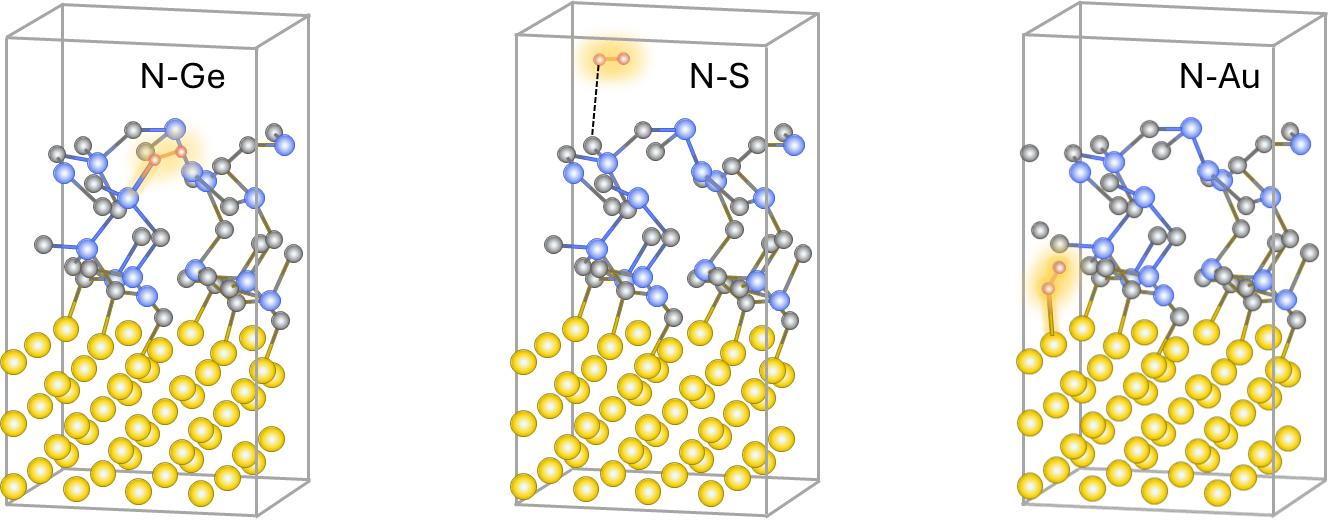


**Figure S38.** Adsorption energy calculation models for N_2_ molecules on Ge, S and Au sites in **GeS_x_-Au**, respectively.


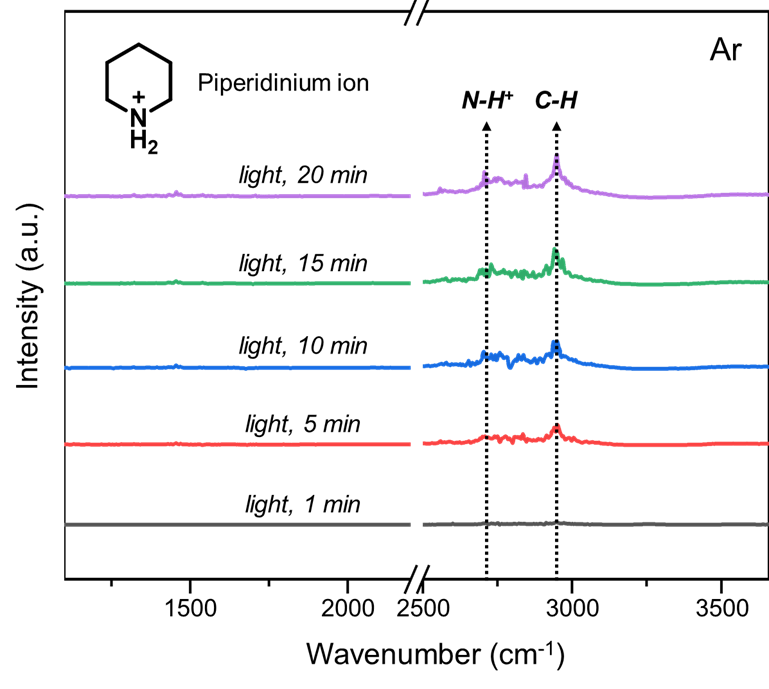


**Figure S39.** In situ IR spectra of **GeS_x_-Au** with or without NIR light with Ar atmosphere.


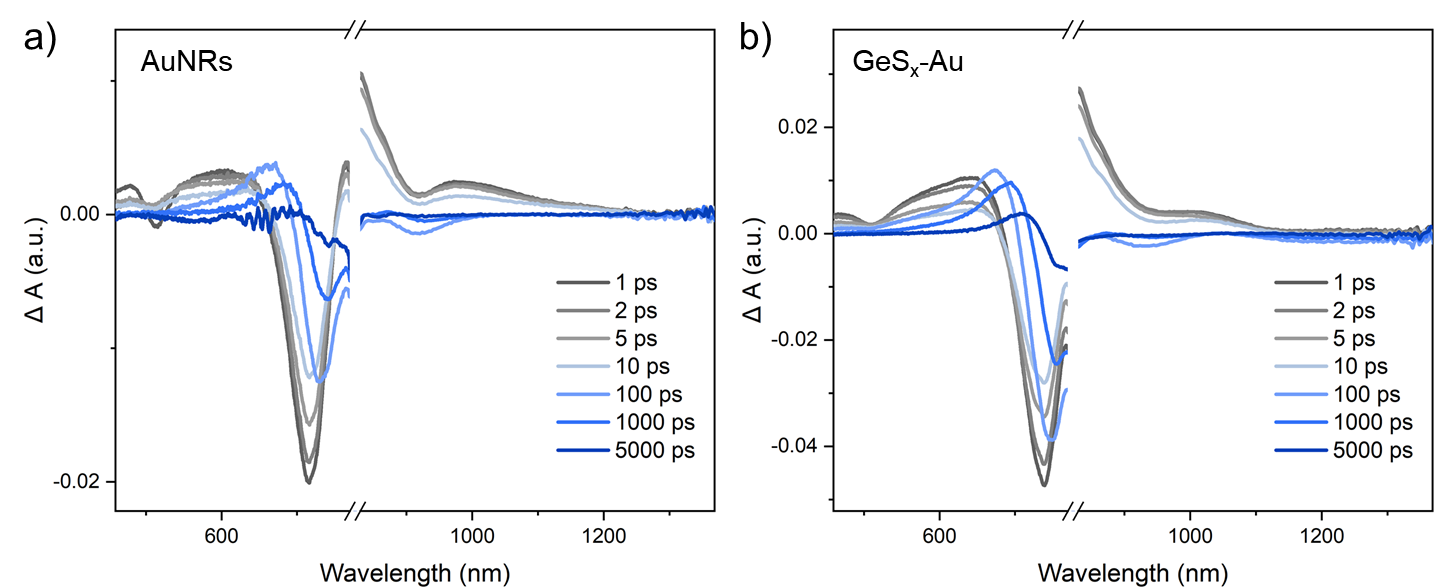


**Figure S40.** Transient spectra of (a) **AuNRs** and (b) **GeS_x_-Au** at different time delay from 1 to 5000 ps obtained upon 800 nm excitation.

**Table S1.** EXAFS fitting parameters at the Co *K*-edge for various samples (*Ѕ*_0_^2^ = 0.1).

| **Sample** | **Shell** | ***CN^a^*** | ***R*(Å)*^b^*** | ***σ*^2^(Å^2^)*^c^*** | **Δ*E*_0_(eV)*^d^*** | ***R* factor** |
| --- | --- | --- | --- | --- | --- | --- |
| Ge foil | Ge-Ge | 4* | 2.45±0.01 | 0.0043 | 5.0±0.7 | 0.0025 |
| T2-Ge | Ge-S | 2.4±0.1 | 2.25±0.01 | 0.0063 | 5.6±0.9 | 0.0068 |
| **GeS_x_-Au** | Ge-S | 1.2±0.2 | 2.32±0.02 | 0.0036 | 12.5±3.1 | 0.0066 |
| GeO_2_ | Au-Au | 8.0±0.2 | 2.84±0.01 | 0.0099 | 4.3±1.1 | 0.0042 |

*^a^CN*, coordination number; *^b^R*, the distance to the neighboring atom; *^c^σ*^2^, the mean square relative displacement (MSRD); *^d^ΔE*_0_, inner potential correction; *R* factor indicates the goodness of the fit. *S*_0_^2^ was fixed to 1, according to the experimental EXAFS fit of Ge foil by fixing *CN* as the known crystallographic value. *This value was fixed during EXAFS fitting, based on the known structure of Ge. Fitting range: 3.0 ≤ *k* (/Å) ≤ 13.9 and 1.0 ≤ *R* (Å) ≤ 3.0 (Ge foil); 3.0 ≤ *k* (/Å) ≤ 11.0 and 1.0 ≤ *R* (Å) ≤ 2.4 (T2-Ge); 3.0 ≤ *k* (/Å) ≤ 11.6 and 1.0 ≤ *R* (Å) ≤ 2.4 (**GeS_x_-Au**); 3.0 ≤ *k* (/Å) ≤ 13.1 and 1.0 ≤ *R* (Å) ≤ 2 (GeO_2_). A reasonable range of EXAFS fitting parameters: 0.700 < *Ѕ*_0_^2^ < 1.000; *CN >* 0; *σ*^2^ > 0 Å^2^; |Δ*E*_0_| < 10 eV; *R* factor < 0.02.

**Table S2.** EXAFS fitting parameters at the Au L3-edge for various samples (*Ѕ*_0_^2^ = 0.87).

| **Sample** | **Shell** | ***CN^a^*** | ***R*(Å)*^b^*** | ***σ*^2^(Å^2^)*^c^*** | **Δ*E*_0_(eV)*^d^*** | ***R* factor** |
| --- | --- | --- | --- | --- | --- | --- |
| Au foil | Au-Au | 12* | 2.86±0.01 | 0.0082 | 4.0±0.5 | 0.0058 |
| **GeS_x_-Au** | Au-S | 0.5±0.1 | 2.28±0.01 | 0.0063 | 2.8±0.5 | 0.0054 |
|  | Au-Au | 8.0±0.2 | 2.84±0.01 | 0.0099 |  |  |

*^a^CN*, coordination number; *^b^R*, the distance to the neighboring atom; *^c^σ*^2^, the mean square relative displacement (MSRD); *^d^ΔE*_0_, inner potential correction; *R* factor indicates the goodness of the fit. *S*_0_^2^ was fixed to 0.87, according to the experimental EXAFS fit of Au foil by fixing *CN* as the known crystallographic value. *This value was fixed during EXAFS fitting, based on the known structure of Au. Fitting range: 3.0 ≤ *k* (/Å) ≤ 13.9 and 1.0 ≤ *R* (Å) ≤ 3.0 (Au foil); 3.0 ≤ *k* (/Å) ≤ 12.2 and 1.0 ≤ *R* (Å) ≤ 3.0 (**GeS_x_-Au**). A reasonable range of EXAFS fitting parameters: 0.700 < *Ѕ*_0_^2^ < 1.000; *CN >* 0; *σ*^2^ > 0 Å^2^; |Δ*E*_0_| < 10 eV; *R* factor < 0.02.

**Table S3.** ICP results of **GeS_x_-Au**.

| **Sample** | **Concentration (mg/L)** |
| --- | --- |
| Au | 863.12 |
| Ge | 0.44 |
| S | 0.42 |

**Table S4** Equivalent circuit fitting results.

|  | **T2** | **GeS_x_-Au** |
| --- | --- | --- |
| Rs | 1.329 | 32.1 |
| C1 | 3.245E-07 | 0.0002098 |
| N1 | 0.9937 | 1 |
| R1 | 22.8 | 1458 |
| C2 | 0.00008854 | 0.00002262 |
| N2 | 0.9286 | 0.9583 |
| R2 | 307.1 | 333900 |
| C3 | 0.0001465 |  |
| N3 | 0.838 |  |
| R3 | 14370 |  |

**Table S5.** Reaction optimization for photocatalytic nitrogen fixation.

| Entry | Deviation | NH_4_^+^ Production Rate  (μmol g^-1^ h^-1^) | N_2_H_4_ Production Rate  (μmol g^-1^ h^-1^) |
| --- | --- | --- | --- |
| 1 | none | 1259.2 | —— |
| 2 | **AuNRs** as PC | 226.7 | —— |
| 3 | T2 as PC | —— | —— |
| 4 | without PC | —— | —— |
| 5 | without MeOH | —— | —— |
| 6 | r.t. in dark | —— | —— |
| 7 | 50 °C, in dark | 20.7 | —— |
| 8 | Ar instead of N_2_ | —— | —— |
| 9 | visible light | 473.3 | 103.3 |
| 10 | full-spectrum light | 505.4 | 163.1 |
| 11 | H_2_O/CH_3_CN = 3/5 | 458.8 | —— |
| 12 | H_2_O/CH_3_CN = 5/5 | 853.2 | —— |
| 13 | H_2_O/CH_3_CN = 5/9 | 1010.9 | —— |
| 14 | 4-methoxybenzenethiol instead of MeOH | 115.7 | —— |
| 15 | EtOH instead of MeOH | —— | —— |
| 16 | Na_2_SO_3_ instead of MeOH | 163.5 | —— |
| 17 | MeOH 0.5 mL | 1014.9 | —— |
| 18 | MeOH 1.5 mL | 1217.4 | —— |
| 19 | P2-In-Au as PC | 521.1 | —— |
| 20 | P1-Zn-Au as PC | 319.1 | —— |
| 21 | P1-Mn-Au as PC | 371.6 | —— |
| 22 | T2-Ge/AuNRs = 0.68/1 | 400.8 | —— |
| 23 | T2-Ge/AuNRs = 1.29/1 | 836.4 | —— |
| 24 | T2-Ge/AuNRs = 1.60/1 | 704.8 | —— |
| 25 | solvent volume in 5 mL | 756.2 | —— |
| 26 | solvent volume in 8 mL | 804.1 | —— |

**Table S6.** Photocatalytic hydrogen evolution experiments in different atmospheres in isotopic solvents.

| **Sample** | **Atmosphere** | **H_2_ Production Rate**  **(μmol g^-1^ h^-1^)** |
| --- | --- | --- |
| 1 | N_2_ | <0.1 |
| 2 | Ar | <0.1 |

**Table S7.** Photocatalytic hydrogen evolution experiments under different atmospheres and light conditions.

| **Sample** | **Atmosphere** | **Light** | **H_2_ Production Rate**  **(μmol g^-1^ h^-1^)** |
| --- | --- | --- | --- |
| 1 | N_2_ | full spectrum | 0 |
| 2 | N_2_ | 780-2200 nm | <0.1 |
| 3 | Ar | full spectrum | <0.1 |
| 4 | Ar | 780-2200 nm | <0.1 |

**Table S8.** Comparisons of N_2_ fixation performance for **GeS_x_-Au** and recently reported photocatalysts.

| Photocatalyst | Light (nm) | NH_4_^+^ Production Rate  (μmol g^-1^ h^-1^) | Sacrificial reagent | Ref. |
| --- | --- | --- | --- | --- |
| GeS_x_-Au | 600-2500 | 1252.9 | CH_3_OH | This work |
| ZnAl-LDH | 200-800 | 110 | —— | 9 |
| Bi/BSO | artificial sunlight | 284.5 | —— | 10 |
| CoS_x_/ZnS | artificial sunlight | 1175.37 | —— | 11 |
| GDY@FeB | ≥ 400 | 1762.35 | —— | 12 |
| ZnCoS_x_@Fe_3_S_4_HS | artificial sunlight | 2523.4 | —— | 13 |
| Au NBP/Rh SSs | > 420 | 138.2 | CH_3_OH | 14 |
| MoO_3-x_ | artificial sunlight | 435.57 | CH_3_OH | 15 |
| Au/HCNs-NV | artificial sunlight | 783.4 | CH_3_OH | 16 |
| Bi_2_MoO_6_ | > 420 | 95.5 | —— | 17 |
| Bi_2_Sn_2_O_7_ | artificial sunlight | 231 | —— | 18 |
| Bi_2_Ti_2_O_7_ | artificial sunlight | 332.07 | —— | 19 |
| COF1-Au | artificial sunlight | 427.9 | K_2_SO_3_ | 20 |
| Cu-Bi_24_O_31_Br_10_ | artificial sunlight | 291.1 | —— | 21 |
| DPPCOOH-COF | > 420 | 521.37 | —— | 22 |
| MIL-125(Ti) | artificial sunlight | 156.9 | —— | 23 |
| MIL-125-NH_2_@CoHHHTP | artificial sunlight | 2100 | —— | 24 |
| P-Fe/W_18_O_49_ | artificial sunlight | 187.6 | Na_2_SO_3_ | 25 |
| Ru@F-TiO_2-x_ | 200-1500 | 58.8 | —— | 2 |
| SiP NSs | > 420 | 35 | EtOH | 36 |
| ZNCoS_x_@Fe_3_S_4_HS | ≥ 800 | 457.3 | —— | 13 |
| Bi_2_S_3_ | 700-1100 | 33.37 | —— | 27 |
| CeF_3_:Yb^3+^, Er^3+^/Fe-ATP | ≥ 780 | 40.3 | —— | 28 |
| MoO_3-x_ Nanosheets | > 580 | 251 | —— | 29 |
| V_O_-S-rich TiO_2−x_S_y_ | ≥ 800 | 86.2 | —— | 30 |

**Table S9.** Comparisons for nitrogen fixation AQY of the **GeS_x_-Au** and recently reported photocatalysts.

| Photocatalyst | Light (nm) | Solvent | Sacrificial reagent | AQY (%) | Ref. |
| --- | --- | --- | --- | --- | --- |
| GeS_x_-Au | 880 | CH_3_CN/H_2_O | CH_3_OH | 1.23 | This work |
| MoO_3-x_ Nanosheets | 808 | H_2_O | — | 0.31 | 33 |
| MoO_3-x_ Nanosheets | 905 | H_2_O | — | 0.22 | 33 |
| MoO_3-x_ | 808 | H_2_O | CH_3_OH | 1.24 | 19 |
| MoO_3-x_ | 1064 | H_2_O | CH_3_OH | 1.12 | 19 |
| NYF/NV-CNNTs | 980 | H_2_O | EtOH | 0.5 | 35 |
| DTiO_2_ | 780 | — | — | 0.07 | 36 |
| Ti_3_C_2_T_x_/TiO_2_ | 740 | — | — | 0.07 | 37 |
| Au/HCNS-NV | 650 | H_2_O | CH_3_OH | 0.18 | 20 |
| Au/HCNS-NV | 700 | H_2_O | CH_3_OH | 0.014 | 20 |
| Ru@F-TiO_2-x_ | 800 | — | — | 0.08 | 6 |
| Ru@F-TiO_2-x_ | 600 | — | — | 0.22 | 6 |
| Bi_2_MoO_6_ | 550 | H_2_O | — | 0.02 | 21 |
| Bi_2_MoO_6_ | 450 | H_2_O | — | 0.172 | 21 |
| Bi_2_Ti_2_O_7_ | 550 | H_2_O | — | 0.12 | 23 |
| Bi_2_Ti_2_O_7_ | 490 | H_2_O | — | 0.36 | 23 |
| COF1-Au | 455 | H_2_O | K_2_SO_3_ | 0.29 | 24 |
| COF1-Au | 420 | H_2_O | K_2_SO_3_ | 0.22 | 24 |
| DPPCOOH-COF | 450 | H_2_O | — | 0.54 | 26 |

**Table S10.** In situ XPS of **GeS_x_-Au** for Au 4f and Ge 2p regions.

| **Entry** | **Conditions** | **Au 4f (eV)** | | **Ge 2p (eV)** | |
| --- | --- | --- | --- | --- | --- |
| 1 | dark, vacuum | 83.9 | 87.6 | 1219.0 | 1249.9 |
| 2 | dark, N_2_, 20 min | 84.0 | 87.7 | 1218.2 | 1249.5 |
| 3 | light, N_2_, 5 min | 83.9 | 87.7 | 1218.1 | 1249.2 |
| 4 | light, N_2_, 20 min | 83.9 | 87.7 | 1218.1 | 1249.2 |

**Table S11.** Lifetimes of **AuNRs** and **GeS_x_-Au** obtained from global analysis.

| **Sample** | **SAS1** | **SAS2** | **SAS3** |
| --- | --- | --- | --- |
| **AuNRs** | τ = 2.17 ps | τ = 27.04 ps | τ = 768.46 ps |
| **GeS_x_-Au** | τ = 4.83 ps | τ = 97.03 ps | τ = 1461.03 ps |

Global analysis was used for the analysis of the data, which involves the R-package TIMP and the open-source graphical user interface GloTarAn. It is based on an excited-state deactivation kinetic model, which helps to obtain the species-associated spectra and the population dynamics of the deconvoluted components.^34^

**6. References**

(1) H. Bai, S. H. Lam, J. Yang, X. Cheng, S. Li, R. Jiang, L. Shao, J. Wang, *Adv. Mater.* *34*, 2104226.

(2) R. Fu, Z. Wu, Z. Pan, Z. Gao, Z. Li, X. Kong, L. Li, *Angew. Chem. Int. Ed.* **2021**, *60*, 11173-11179. *Angew. Chem*. **2021**, *133*,11097.

(3) J. Li, C. Zhang, T. Bao, Y. Xi, L. Yuan, Y. Zou, Y. Bi, C. Liu, C. Yu, *Adv. Mater.* **2024**, *37*, 2416210.

(4) H. Funke, M. Chukalina, A. Rossberg, *Phys. Scr.* **2005***, 232.*

(5) S. I. Zabinsky, J. J. Rehr, A. Ankudinov, R. C. Albers, M. J. Eller, *Phys. Rev. B* **1995**, *52*, 2995–3009.

(6) J. VandeVondele, J. Hutter, *J. Chem. Phys.* **2007***,* *127*, 114105.

(7) J. P. Perdew, K. Burke, M. Ernzerhof, *Phys. Rev. Lett.* **1997***,* *78*, 1396.

(8) S. Goedecker, M. Teter, J. Hutter, *Phys. Rev. B* **1996**, *54*, 1703–1710.

(9) S. Zhang, Y. Zhao, R. Shi, C. Zhou, G. I. N. Waterhouse, L. Wu, C. Tung, T. Zhang, *Adv. Energy Mater.* **2020***, 10,* 1901973.

(10) R. Wu, S. Gao, C. Jones, M. Sun, M. Guo, R. Tai, S. Chen, Q. Wang, *Adv. Funct. Mater.***2024***, 34,* 2314051*.*

(11) T. Bao, Y. Xi, C. Zhang, P. Du, Y. Xiang, J. Li, L. Yuan, C. Yu, C. Liu, *Natl. Sci. Rev.***2024**, *11*, nwae093.

(12) Y. Fang, Y. Xue, L. Hui, H. Yu, Y. Li, *Angew. Chem. Int. Ed.***2021***, 60,* 3170.

(13) J. Li, C. Zhang, T. Bao, Y. Xi, L. Yuan, Y. Zou, Y. Bi, C. Liu, C. Yu, *Adv. Mater.***2024***, 37，*2416210.

(14) Y. Yang, H. Jia, N. Hu, M. Zhao, J. Li, W. Ni, C. Zhang, *J. Am. Chem. Soc.***2024***, 146, 11,* 7734–7742.

(15) H. Bai, S. H. Lam, J. Yang, X. Cheng, S. Li, R. Jiang, L. Shao, J. Wang,*Adv. Mater.***2022***, 34,* 2104226*.*

(16) Y. Guo, J. Yang, D. Wu, H. Bai, Z. Yang, J. Wang, B. Yang, *J. Mater. Chem. A* **2020**, *8*, 16218–16231.

(17) C. Yang, Y. Zhang, F. Yue, R. Du, T. Ma, Y. Bian, R. Li, L. Guo, D. Wang, F. Fu, *Appl. Catal. B Environ.* **2023**, *338*, 123057.

(18) S. Gao, R. Wu, M. Sun, M. Guo, D. B. DuBois, S. Chen, H. Ji, C. Wang, Q. Wang, *Appl. Catal. B Environ.* **2023**, *324*, 122260.

(19) P. Li, R. Wu, P. Li, S. Gao, Z. Qin, X. Song, W. Sun, Z. Hua, Q. Wang, S. Chen, *Adv. Sci*. **2024**，*11*, 2408829.

(20) T. He, Z. Zhao, R. Liu, X. Liu, B. Ni, Y. Wei, Y. Wu, W. Yuan, H. Peng, Z. Jiang, Y. Zhao, *J. Am. Chem. Soc.* ***2023****, 11,* 6057–6066.

(21) J. Di, C. Chen, Y. Wu, Y. Zhao, C. Zhu, Y. Zhang, C. Wang, H. Chen, J. Xiong, M. Xu, J. Xia, J. Zhou, Y. Weng, L. Song, S. Li, W. Jiang, Z. Liu, *Adv. Mater.* **2022**, *34*, 2204959.

(22) X. Zhang, Y. Liu, S. Feng, X. Gu, M. Zhou, H. Wang, J. Hua, *Appl. Catal. B Environ.* **2025**, *366*, 125013.

(23) Y. Sun, H. Ji, Y. Sun, G. Zhang, H. Zhou, S. Cao, S. Liu, L. Zhang, W. Li, X. Zhu, H. Pang, *Angew. Chem. Int. Ed.* **2024***, 63,* e202316973. *Angew. Chem.* **2024**, *136*, e202319142.

(24) L. Yuan, C. Tang, P. Du, J. Li, C. Zhang, Y. Xi, Y. Bi, T. Bao, A. Du, C. Liu, C. Yu, *Angew. Chem. Int. Ed.* **2024***, 63,* e202412340. *Angew. Chem.* **2024**, *136*, e202421733.

(25) G. Dong, X. Huang, Y. Bi, *Angew. Chem. Int. Ed.* **2022**, *61*, e202204271. *Angew. Chem.* **2022**, *134*, e202207941

(26) Y.-J. Yuan, N. Lu, L. Bao, R. Tang, F.-G. Zhang, J. Guan, H.-D. Wang, Q.-Y. Liu, Q. Cheng, Z.-T. Yu, Z. Zou, *ACS Nano* **2022**, *16*, 12174–12184.

(27) M. Lan, Y. Wang, X. Dong, F. Yang, N. Zheng, Y. Wang, H. Ma, X. Zhang, *Appl. Surf. Sci.* **2022**, *591*, 153205.

(28) S. Zuo, H. Zhang, X. Li, C. Han, C. Yao, C. Ni, *ACS Sustainable Chem. Eng.* **2022***, 10,* 1440–1450.

(29) H. Wu, X. Li, Y. Cheng, Y. Xiao, R. Li, Q. Wu, H. Lin, J. Xu, G. Wang, C. Lin, X. Chen, Y. Wang, *J. Mater. Chem. A* **2020**, *8*, 2827–2835.

(30) X. Xue, H. Chen, Y. Xiong, R. Chen, M. Jiang, G. Fu, Z. Xi, X. L. Zhang, J. Ma, W. Fang, Z. Jin, *ACS Appl. Mater. Interfaces* **2021***, 13,* 4975–4983.

(31) Y. Zhu, X. Zheng, W. Zhang, A. Kheradmand, S. Gu, M. Kobielusz, W. Macyk, H. Li, J. Huang, Y. Jiang, *ACS Appl. Mater. Interfaces* **2021***, 13,* 32937–32947.

(32) Y. Zhang, X. Chen, S. Zhang, L. Yin, Y. Yang, *Chem. Eng. J.* **2020**, *401*, 126033.

(33) T. Hou, Q. Li, Y. Zhang, W. Zhu, K. Yu, S. Wang, Q. Xu, S. Liang, L. Wang, *Appl. Catal. B Environ.* **2020**, *273*, 119072.

(34) J. J. Snellenburg, S. Laptenok, R. Seger, K. M. Mullen, H. M. Stokkum, *J. Stat. Softw.* **2012**, *49*, 1-22.
